# Metal bioaccumulation alleviates the negative effects of herbivory on plant growth

**DOI:** 10.1038/s41598-021-98483-x

**Published:** 2021-09-24

**Authors:** Grazieli F. Dueli, Og DeSouza, Servio P. Ribeiro

**Affiliations:** 1 Programa de Pós-Graduação em Ecologia, Departamento de Biologia Geral, Universidade Federal de Viçosa, Viçosa, Brazil; 2 Laboratório de Termitologia, Departamento de Entomologia, Universidade Federal de Viçosa, Viçosa, Brazil; 3 Laboratório de Ecologia do Adoecimento e Florestas, Núcleo de Pesquisas em Ciências Biológicas, Instituto de Ciências Biológicas e Exatas, Universidade Federal de Ouro Preto, Ouro Prêto, Brazil

**Keywords:** Ecology, Ecology

## Abstract

Metalliferous soils can selectively shape plant species’ physiology towards tolerance of high metal concentrations that are usually toxic to organisms. Some adapted plant species tolerate and accumulate metal in their tissues. These metals can serve as an elemental defence but can also decrease growth. Our investigation explored the capacity of natural metal accumulation in a tropical tree species, *Eremanthus erythropappus* (Asteraceae) and the effects of such bioaccumulation on plant responses to herbivory. Seedlings of *E. erythropappus* were grown in a glasshouse on soils that represented a metal concentration gradient (Al, Cu, Fe, Mn and Zn), and then the exposed plants were fed to the herbivores in a natural habitat. The effect of herbivory on plant growth was significantly mediated by foliar metal ion concentrations. The results suggest that herbivory effects on these plants change from negative to positive depending on soil metal concentration. Hence, these results provide quantitative evidence for a previously unsuspected interaction between herbivory and metal bioaccumulation on plant growth.

## Introduction

Metalliferous soils are selective forces shaping plant species’ physiology towards tolerance to high metal concentration, usually toxic to living organisms^1^. These adapted plant species, called metallophytes^2^, are adjusted to exclude, tolerate or accumulate metal ions in their tissues during their lives^3–5^. Often, the same plant may be accumulating certain metals and tolerating/excluding others or changing its strategy according to seasonal characteristics^6^.

Metallophytes that accumulate high concentrations of metal in their tissues are called hyperaccumulators^7^. Plants that do not fit hyperaccumulation patterns have been named only accumulators, and the tissue metal concentrations are between those of non-accumulators and hyperaccumulators^8^. These criteria are not absolute but may serve as a guide to characterise metal ions accumulation until physiological processes are better explained^9^. Finally, these values may vary according to the geographic region of the plant, as demonstrated by Metali et al.^10^.

The drivers for the evolution of metal accumulation are still subject of debate^11^. Several hypotheses have been proposed to explain this strategy, such as: (i) plant protection against pathogens and herbivores or “elemental defence hypothesis”^12^; (ii) a competitive advantage over non-accumulating plants by allelopathy^5,13,14^, which occurs when hyperaccumulating plants enrich the surrounding soil with metallic leaves, making the environment toxic to non-tolerant plants^15^; (iii) the remediation of metabolic defects, as demonstrated by Freeman et al.^16^ in which the Ni hyperaccumulator *Thlaspi* (Brassicaceae) reversed the hypersensitivity to pathogens; (iv) a drought tolerance mechanism^17,18^ and (v) an accidental consequence for more effective nutrient absorption^17^. From these hypotheses, “elemental defence” is the most investigated one, based on both the death of herbivores that ingest the hyperaccumulating plant tissues^12^ and on the deterrent action of metals that prevent the ingestion of plant tissues by herbivores^18^. A synergistic action of herbivory and metal bioaccumulation on plant growth, however, has never been reported, to the best of our knowledge.

As herbivory is one of the causes of decreased plant fitness^19,20^, accumulating metals without affecting plant physiology^21^ can be an effective and a low-cost defence strategy. Metals, unlike organic compounds, cannot be degraded by herbivores^12^. Noret et al.^11^ showed that some accumulators synthesised fewer secondary compounds than non-accumulating plants, reducing their energy expenditure in defence against herbivores. The costs of adapting to metalliferous soils are associated with the energy expended by the plant to detoxify the metal ions and might reflect in reduced growth rates^22–25^ as well as limited energy expenditure in development and reproduction^24,26^. Thus, minimising herbivory through metal accumulation could be an evolutionary compensation for the physiological costs of metal accumulation^27^.

Various studies corroborate the hypothesis of elemental defence in hyperaccumulating plants^18,28–31^. However, recent studies^27,32,33^ have also shown that low levels of accumulated metals (Cd, Mn, Ni, Pb and Zn, for example) in plants may likewise reduce herbivory. Coleman et al.^32^ believe that the elemental defence hypothesis should be more widespread among plants than has been demonstrated. According to Boyd^34^, many elements still need to be further tested (e.g., Al, B, Fe).

Soils rich in metals are abundant in the tropics and very typical of the Ferriferous Quadrangle (FQ) region, in the central portion of the State of Minas Gerais, Brazil. In the Canga (ironstone crops) of the FQ, studies have documented different woody species that hyperaccumulate metals in their leaves^35,36^, but studies on the ecological effects of metal accumulation are still scarce for such plant species. Among the hyperaccumulating species of the FQ, *Eremanthus erythropappus* DC. MacLeish (Asteraceae) stands out. *Eremanthus erythropappus*, popularly known as candeia, is a deciduous, heliophytic and xerophytic tree. It can be found in Southeastern Brazil in high-montane or subtropical ombrophilous forests^37^. Candeia occurs in habitats harsh for non-adapted species to survive, such as shallow, nutrient-poor and rocky soils at altitudes between 900 and 1800 m^38^. Furthermore, candeia occurs on metal-rich soils, where they form monodominant forest stands called “candeial”. Adult individual trees in a candeial accumulate metal ions in their leaves, with considerable effects on herbivory levels^27^.

In the present study, *E. erythropappus* were grown in soils with different metal concentrations (Al, Cu, Fe, Mn and Zn) to test the hypothesis that bioaccumulation of metals in *E. erythropappus* represents a trade-off between two conflicting effects of metals: (i) their impairment of plant growth and (ii) their deterrence of herbivory. If this is so, we predict that physiological costs related to metal bioaccumulation would be compensated for by the herbivory inhibition effects on the growth of such plants. The results of our study showed how an environmental constraint, here represented by the excess of metals in the soil, changed the herbivore-plant interaction from negative to positive, positively affecting the growth of candeia.

## Results

**Figure 1:**
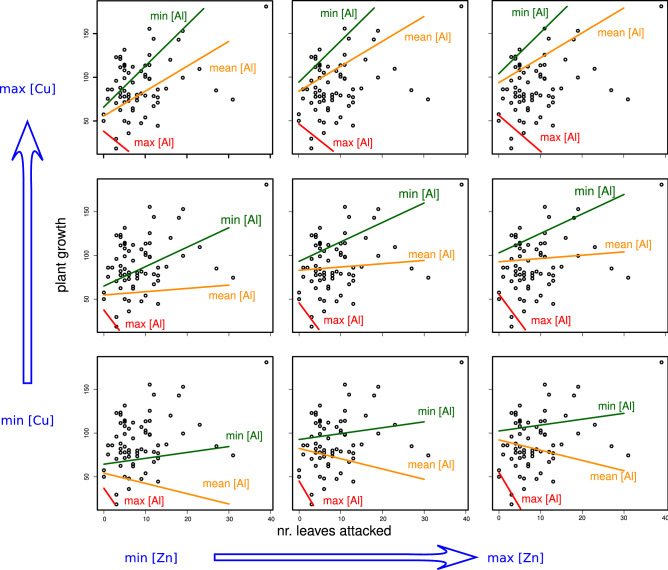
The effects of herbivory on the growth of candeia plants mediated by Al (aluminium), Cu (copper) and Zn (zinc) foliar content (mg kg^−1^). Leaf content in Zn and Cu increases from top left to bottom right. Curves represent the candeias’ growth in minimal (min [Al]—green lines in all panels), average (mean [Al]—yellow lines in all panels) and maximal (max [Al]—red lines in all panels) as detected in leaves. Some curves for maximum [Al] have been reallocated 20 units higher, to ease visualization.

Raw data are presented in Supplementary Tables S1 and S2 and Supplementary Figure S1 online. Under the effects of metal and in the absence of herbivory in the glasshouse, the mean seedling growth over 305 days (10 months) was 89 cm, ranging from 17 to 173.5 cm. The concentrations of Al^3+^, Cu^2+^, Fe^2+^, Mn^2+^ and Zn^2+^ in the candeias’ leaves did affect the candeias’ growth (Fig. 1, Table 1, Table S1 online). In the field experiment, 58 out of 68 plants used were attacked by herbivorous invertebrates (85.3%), but the mean percentage of leaf area lost (0.95%) was extremely low. The average number of attacked leaves was nine leaves per plant, ranging from 0 up to 39 leaves. Most importantly, the concentration of Al^3+^, Cu^2+^, Fe^2+^, Mn^2+^ and Zn^2+^ in the candeias’ leaves did affect herbivory (Table 2).

**Table 1:** Model selection table—model with substantial empirical evidence (∆ ≤ 2) predicting metal effects in candeias’ growth (growth_rate1) cultivated in soils with different metal concentrations in a glasshouse in the absence of herbivory.

Model	(Int)	Al	Cu	Fe	Mn	Zn	df	logLik	AICc	∆	Weight
1	89.04						2	− 331.43	667.0	0.00	0.21
2	71.51	0.09021					3	− 330.61	667.6	0.56	0.159
18	108.70	0.13380				− 0.43	4	− 329.68	668.0	0.95	0.131
26	108.10	0.14750			0.01	− 0.6	5	− 328.63	668.2	1.19	0.12
3	77.71		0.79330				3	− 331	668.4	1.32	0.11
9	79.48				0.01		3	− 331.06	668.5	1.45	0.1
17	110.40					− 0.2	3	− 331.19	668.8	1.71	0.09
5	83.17			0.01			3	− 331.23	668.8	1.79	0.086

Explanatory variables are metal concentrations (Al, Cu, Fe, Mn and Zn). Models are based on 68 independent observations (plants) and refer to a multiple regression with generalized linear models under Gaussian and identity-link function. Int = Intercept, df = degrees of freedom used by the model, Loglik = log-likelihood, AICc = Second-Order Akaike Information Criterion, ∆ = AICc difference between the model under concern and the best model, Weight = Akaike weight, that is, the likelihood of the present model being the best in the candidate set. Global model: growth_rate1 ~ (Al + Cu + Fe + Mn + Zn).

**Table 2:** Model selection table—model with substantial empirical evidence (∆ ≤ 2) predicting metal and herbivory effects in candeias’s growth (growth_rate).

Model	(Int)	Al	atc_lvs	Cu	Fe	Mn	Zn	Al:atc_lvs	Cu:atc_lvs	Zn:atc_lvs	df	logLik	AICc	∆	Weight
232	46.090	− 0.1354	2.380	0.07			0.58	− 0.02443	0.19		8	− 311.98	642.4	0.00	0.238
104	20.580	− 0.1318	5.838	1.66500			0.6	− 0.02740			7	− 313.44	642.7	0.35	0.200
1192	− 5.969	− 0.2250	8.780	− 0.83430			1.3010		0.28	− 0.10780	8	− 312.28	643.0	0.61	0.175
1256	− 4.480	− 0.1580	8.952	− 0.35770			1.1270	− 0.01832	0.23	− 0.07453	9	− 311.05	643.2	0.82	0.158
1200	− 6.675	− 0.1875	8.711	− 1.03800	− 0.03		1.3860		0.3	− 0.11080	9	− 311.26	643.6	1.22	0.129
248	49.150	− 0.1239	2.028	− 0.03668		0.01	0.5050	− 0.02482	0.22		9	− 311.52	644.1	1.74	0.100

Plants were initially cultivated in glasshouse on soils with different metal concentrations and then transplanted to the field. Explanatory variables include (1) metal concentrations (Al, Cu, Fe, Mn and Zn) and (2) number of leaves attacked by herbivores (atc_lvs). Models are based on 68 independent observations (plants) and refer to a multiple regression with generalized linear models under Gaussian and identity-link function. Int = Intercept, df = degrees of freedom used by the model, Loglik = log-likelihood, AICc = Second-Order Akaike Information Criterion, ∆ = AICc difference between the model under concern and the best model, Weight = Akaike weight, that is, the likelihood of the present model being the best in the candidate set. Global model: growth_rate ~ (Al + Cu + Fe + Mn + Zn)* atc_lvs.

**Figure 2:**
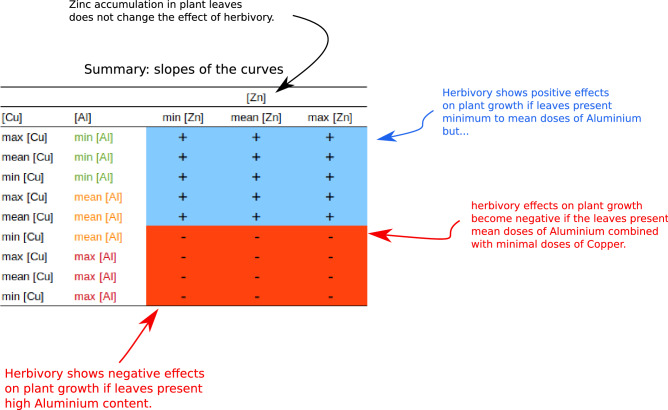
The positive and negative effects of herbivory on candeia plants growth, as determined by metal bioaccumulation in these plant leaves. Here we show a summarised view of the plots presented at this figure. Plus (+) and minus (−) signs represent the slope of the curves describing the effects herbivory on plants growth. Each cell in this table is a single curve in the plots of Fig. 1. Metal content on leaves is indicated as in Fig. 1.

Under the combined effects of metal and herbivory, the candeias’ growth reached a mean of 89.5 cm in 4 months, ranging from 18.5 cm up to 181.0 cm. The effect of herbivory on the candeias’ growth was significantly mediated by their Al^3+^, Cu^2+^ and Zn^2+^ foliar contents (Figs. 1 and 2, Tables 2 and 3), supporting our hypothesis. Under maximal Al concentration (469.6 mg kg^−1^), plant growth was negatively impacted by increasing herbivory (red lines in all panels of Fig. 1), but this negative effect was reversed under minimal Al concentration (18.65 mg kg^−1^, green lines in all panels of Fig. 1). However, under average Al concentration, herbivory effects on plant growth depended on leaf Cu concentration, starting from negative effects at minimal Cu concentration (6.2 mg kg^−1^) and reverting to positive effects at maximal Cu concentration (27.15 mg kg^−1^). Plants with the highest concentration of Cu, under minimal Al concentration, showed the strongest positive effects of herbivory on plant growth (green lines of Fig. 1). The Zn concentrations of leaves did not affect the slope of curves describing herbivory, even though it affected the ‘baseline’ value of plant growth (that is, the growth under zero herbivory or the intercept value for all curves of Fig. 1).

**Table 3:** Best model among all those with empirical evidence (model # 232 of Table 2) predicting metal and herbivory effects on candeias’ growth (y).

Estimate		Std. error	t value	Pr(> |z|)
**y = mean growth of *Eremanthus erythropappus***				
(Intercept)	46.09	32.25	1.43	0.1580
Al	− 0.14	0.08	− 1.75	0.0845
Cu	0.07	1.22	0.05	0.9571
Zn	0.58	0.27	2.19	0.0323*
atc_lvs	2.38	2.92	0.82	0.4185
Al:atc_lvs	− 0.02	0.01	− 2.14	0.0366*
Cu:atc_lvs	0.19	0.12	1.64	0.1067

Plants were initially cultivated in glasshouse on soils with different metal concentrations and then transplanted to the field. Codes for variables are the same as in Table 2. This was the model chosen to plot Fig. 1

*P < 0.05, **P < 0.01, ***P < 0.001.

As the Cu concentrations in leaves increase, so does the strength of the positive effects of herbivory on plant growth under mean and minimal Al concentrations, as shown by the increasing slope of green and yellow lines of Fig. 1. Copper concentration, however, did not affect herbivory under maximal Al concentration (red lines of Fig. 1). Conversely, the Zn concentration seems not markedly relevant for any reversion of the relationship between plants and herbivores. Its only observable effect was a mild increment of plant growth as Zn concentration increases for all titres of Al and especially under low Cu concentration and under zero herbivore attack (Figs. 1 and 2).

## Discussion

Candeia trees accumulated metal ions in their leaves in concentrations smaller than the values found in typical hyperaccumulator organisms^9^, despite growing in soils rich in metals. The concentrations of metal ion in the leaves of candeia trees did not reflect the concentrations of metals in the particular soil in which the plants developed within the 7-level gradient of our experiment (Table 4, Table S1 online), similar to that found in other studies^39,40^. This uncorrelated accumulation can be related to the physical–chemical characteristics of the soils^41^ and their organic matter contents, which interfere with the adsorption of metallic ions by the plants^6,42^.

**Table 4:** Percentage and volume of soil used in each combination from Itacolomi State Park (PEIT) and Padre Viegas (PV) to compond the 7 soil levels and metal concentration (mg kg^−1^) in the soils of each level at which the plants (*Eremanthus erythropappus*) were cultivated.

Soil levels	% soil		Soil volume (L)		Metal concentrations in each soil level (mg kg^−1^)				
	PV	PEIT	PV	PEIT	Al	Cu	Fe	Mn	Zn
1	100	0	8.0	0.0	15,522.00	11.77	42,505.00	92.16	6.95
2	85	15	6.8	1.2	33,402.00	33.73	78,667.25	260.37	42.72
3	70	30	5.6	2.4	44,130.00	46.92	100,364.60	356.50	64.17
4	55	45	4.4	3.6	51,282.00	55.71	114,829.50	422.58	78.48
5	40	60	3.2	4.8	58,434.00	64.47	129,294.40	488.66	92.78
6	25	75	2	6	69,162.00	77.68	146,782.28	587.79	114.65
7	0	100	0.0	8.0	87,042.00	99.65	187,154.00	753.00	150.00

The average level of herbivory found in this study was very low (0.95%) (Table S1 online) as compared to the one reported for adult trees of this plant species in natural conditions (2.8%)^43^ or to other sympatric species found in these forests but adapted to deeper and more nutrient-rich, swampy soils^44^. The herbivory was indeed very low at all metal ion concentrations tested, about 10 times lower than the global average^45,46^. Despite that, the accumulated metal ions affected both the growth of candeia seedlings and the herbivory on their leaves, in agreement with another study from the same region^27^. Even at low concentrations, metals may increase or potentiate their toxic effects on organisms when they are co-accumulated^27,47,48^. Co-accumulation seems to be advantageous because plants can benefit from elemental defences by accumulating lower concentrations of each metal in their tissues^47^^.^

Most importantly, though, the present experiment suggested that interactions classically considered negative, such as herbivory, might shift to positive depending on specific ecophysiological conditions. Namely, the negative effects of herbivory can be reversed depending on the environmental stress, here represented by Al, Cu and Zn (see Figs. 1 and 2, Table 3). Examples of reversed outcomes of species interactions were presented by Bronstein^49^ who showed that these interactions are not static. They can change from positive to negative (or vice versa) and fluctuate in their intensities according to the costs and benefits they provide to organisms^50^.

The reversion of the herbivore-plant interaction output is not at all novel, being well explained in the literature as a compensatory effect. The compensatory effect is the ability of plants to compensate for adverse effects caused by tissue loss, recover organic functionality and maintain normal growth after damage^22^. This has been demonstrated in plants suffering small amounts of damage by herbivores^22,51–53^. This effect may reflect better physiological responses, such as nutrient storage and higher photosynthetic rates, resulting in substantial plant growth regardless of trade-offs caused by a background of herbivory, when it is rather low^51,52^. The mechanisms behind the compensatory effect are complex and involve physiological, developmental and environmental effects^22,51,52,54,55^^.^

The deleterious effects of metals in the early stages of development for plants that are not adapted to metal-rich soils are known^56^. However, we showed in a metal-rich soil specialist, that metal ions accumulation might be beneficial if the concentrations of metal ions are more toxic to the herbivores than to the plants^57^. It is worth noting that among such bioaccumulated metal ions, there are two micronutrients, Cu and Zn, with important biochemical and physiological functions in plants^58^, and Al, which besides having unknown biological function^59–61^, can be toxic depending on its concentration. The maximum concentrations of Cu (27.15 mg kg^−1^) and Zn (123.8 mg kg^−1^) in the leaves were beneficial to the plants and this may be related to the role that these elements have as micronutrients. In contrast, Al at the maximum concentration in the leaves (469.6 mg kg^−1^) was detrimental to the plants.

One relevant evolutionary aspect is that Cu and Zn in excess can induce metallothionein production, which acts both as a regulator of oxidant stress and decontaminant of metals. However, this protein is known to act only on those elements at the fourth to the fifth period in the Periodic Table, which are micronutrients^62,63^. Aluminium is not a heavy metal and is far from the chemical characteristics of the former cited elements. In plants, the process of Al^3+^ uptake depends on soil pH and the chemical environment of the rhizosphere^64^. Although vascular plants have evolved strategies to detoxify Al by storing it in phytoliths^65,66^ or using exclusion mechanisms^67^, its toxicity may mitigate, often resulting in a decrease in plant growth^67–69^. To measure the impact of Al on plant growth, it is necessary to consider other variables such as metal concentration and particular characteristics of each genotype within the same species (growth conditions, physiological age and time of exposure to metal)^69^.

The response of plants to herbivory is quite complex and depends on the environment, genetics, developmental stages, induced responses and affected tissues^22,69,70^. The present investigation evidenced that leaf metal ions concentration may shift the herbivore-plant interaction, reducing its antagonism. Aluminium concentrations seems the most responsible for reversing herbivory effects in plants, irrespective of Zn and Cu concentrations. Copper concentration seems also relevant, being able to modulate the strength herbivore effects on plant growth, at least for leaves holding minimal to medium Al concentration (but not for plants under maximal Al content) (Figs. 1 and 2, Table 3). The present investigation brings about a new approach to herbivore-metallophyte interaction and suggests a new evolutionary possibility for the metal accumulations. The results corroborate the existence of an evolutionary mechanism of compensatory effect that acts on herbivore-plant interaction, modulated by metals. In this environment of little herbivory, there might even be the possibility of the herbivore-plant interaction contributing to faster plant growth by some mechanism that is not yet well understood.

Differences in the compensatory effect on herbivory levels might vary between life stages^55^, as studied by Massad^71^, who showed the negative effect of herbivory only on the growth of seedlings. We acknowledge that such metal-mediated compensatory effect worked for our seedling system, which suffered very little herbivory. Because the plants might be protected by high metal ion concentration in the leaves during the early establishment, this positive metal effect could have long survival consequences. We cannot assure that this mechanism prevails in adult trees that suffer greater and lifelong cumulative herbivory attacks. Studies with metal ion accumulator plants are still scarce^48^ and are necessary to better understand the strategies used to deal with excess metals in the soil. More studies are needed to assess the impacts of the accumulation of metals on candeias lifelong fitness.

The novelty in the present work is to quantitatively show that such a reversion of herbivore-plant interaction may be mediated by metal bioaccumulation, a factor usually regarded as a deterrent to plant growth. The results suggest that metal ions decreased herbivore attack capacity and, due to the low herbivory levels, there followed a compensatory effect on the costs of accumulating metals in the leaves, promoting candeia growth. In this sense, there was an herbivore-plant interaction that was modulated by the metal contents (Al^3+^, Cu^2+^ and Zn^2+^) in candeia leaves, stimulating the reversion of the negative effects of herbivory on plant growth.

## Material and methods

### Rationale

This study complies with the relevant regulations of Brazil, including permission from landowners to conduct the study on their site. Tacit approval from the Brazilian Government is implied by the hiring of authors as scientific researchers. No protected species were sampled. No genetic information was accessed. This research was registered in the Brazilian National System for the Management of Genetic Heritage (SISGEN) under number AC3EE6F.

**Figure 3:**
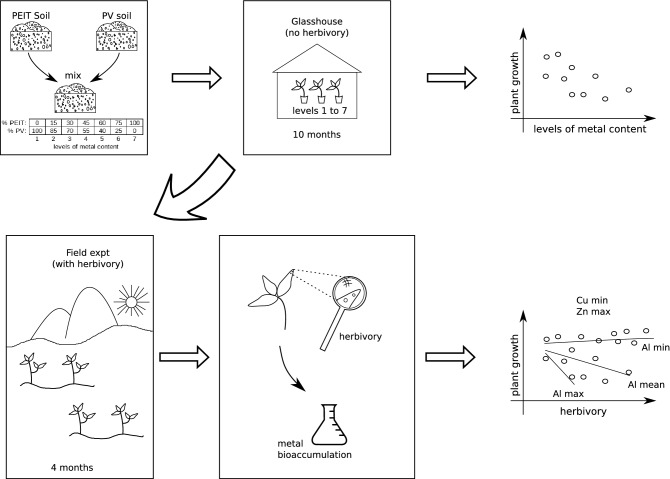
Schematic view of the experiments. We mixed two types of soil, one with low metal content (PV) and another with high metal content (PEIT) varying proportions to produce increasing levels of metal content in the resulting soil mix. Then, seedlings of candeia were grown on these soil mixes for 10 months in a glasshouse, free of herbivores. This has allowed us to access the effects of metal on plant growth independently of the effects of herbivory. After that, we took the plants to the field, where they have been exposed to herbivory for 4 months. We inspected leaves for signs of herbivory and measured the metals bioaccumulated in these leaves, so that to analyse the combined effects of metal and herbivory on plant growth.

Glasshouse and field bioassays were concatenated to investigate our hypothesis. The initial development of plants on soils arranged in a gradient of metal content and without herbivory, was monitored in a glasshouse. Then these plants were exposed to natural herbivory by transferring them to the field. Finally, herbivore attack and metal bioaccumulation in these plants’ leaves were measured, to verify how these affected plant growth. This experimental design allowed the separation of the deleterious effects of metal ions bioaccumulation per se, from the combined effect of metals and herbivory on plant growth (Fig. 3).

### Study sites and soil sampling

Soils were sampled in two areas with different metal concentrations in the Itacolomi State Park (PEIT) and Padre Viegas (PV) rural areas. Both are located in the FQ, the region with the largest iron ore production in the world.

PEIT is a park at a high altitude (900 to 1772 m above sea level), located in the Ouro Preto and Mariana municipalities (20°22′30″–20°30′00″ S and 43°32′30″–43°22′30″ W)^72^, at the southern portion of the Espinhaço Mountain^73^, near the south border of the São Francisco Craton^74^ in Minas Gerais State, Southeastern Brazil. It occupies an area of approximately 70 km^2^. Soils of the region evolved on lithological material, consisting mainly of schists, phylites, and quartzites. They are shallow (Litolics) to moderately deep (Cambisoils), containing easily weathered primary minerals. In these soils, high concentrations of metals such as Al, As, Ba, Cd, Co, Cr, Fe, Pb, Mn and Zn are found (see Table S2 online).

Padre Viegas is a rural area (700 m above sea level) in the Mariana municipality (20°23′44.9″ S, 43°20′55.5″ W), 9 km away from downtown and 50 km from PEIT. The region occupies about 178 km^2^ and is inserted in the lithological Nova Lima group, formed by mafic–ultramafic volcanic rocks, secondary volcanic felsic rocks, and sedimentary chemical and clastic rocks. These rocks contain the main gold deposits of the FQ^75^, which generated intense mineral exploration in the region in the eighteenth century. This region where collections were made has deep soils (Latosols) and lower concentrations of Fe, Mn, Al, Zn and Cu compared to the PEIT soils (see Table S2 online).

### Candeias’ development (glasshouse bioassay)

The candeias used in this experiment were acquired from the State Institute of Forestry’s seedling nursery, in tubes of 110 cm^3^. Seventy seedlings, initially 32 cm high on average and 6 months old, were transplanted into 8 L plastic pots filled with a soil combination from PEIT and PV. Soils were mixed together in different proportions, constituting a concentration gradient of metals ranging from 100% of PEIT soil to 100% of PV soil, arranged in seven levels: level 1 (0% PEIT and 100% PV), level 2 (15% PEIT and 85% PV), level 3 (30% PEIT and 70% PV), level 4 (45% PEIT and 55% PV), level 5 (60% PEIT and 40% PV), level 6 (75% PEIT and 25% PV), level 7 (100% PEIT and 0% PV) (Table 4). Because metal content is lower in PV as compared to PEIT, higher proportion of the latter would provide higher metal content levels. Each of the soil levels was divided into 10 individual planting pots, where one sole plant was transplanted and left to grow. Plants were kept in the ICEB´s glasshouse at the Federal University of Ouro Preto with irrigation automatically provided by a sprinkler system, ensuring relative air humidity of 65% and temperature of 24 °C (Fig. 3). The seedlings’ growth was monitored in the glasshouse, by monthly measurement of plant height over 10 months.

### Herbivory test and plant growth (field bioassay)

After 10 months in the glasshouse, plant pots were translocated to the PEIT, in an area dominated by a candeial. Within this habitat, five microhabitats (blocks) with different gradients of vegetation density were selected, ranging from open, sunny, vegetation to shadowed, relatively dense woody vegetation. Fourteen plants were placed in each block (2 of each soil level), totalling 70 plants. Plants were monitored every 20 days until the end of the field experiment, that is, for 4 months (Fig. 3). At each monitoring event, the number of leaves with any sign of chewing on each plant was counted, based on Ribeiro and Basset^76,77^. The chewed leaves were marked to avoid re-counting them in the next monitoring. During the experiment two plants died by unknown causes. After 4 months in the field, the candeias were taken back to the glasshouse and the height of each plant was measured again. All leaves were collected and saved in paper bags. To measure the lost leaf area, 20 leaves of each plant were randomly sorted and scanned (Fig. S1 online). From the images, the percentages of lost leaf area were calculated using Sigma Scan software. Approximately 5 g of leaves per plant were used to analyse metal concentrations (Al, Cu, Fe, Mn and Zn).

### Soil metals analyses

Soil samples were dried at 40 °C and passed through a fine-mesh sieve (2 mm). About 1.0 g of each sample was weighed in a 100 ml beaker. A small amount of Milli-Q water and 9.3 ml of aqua regia. (7.0 ml of HCl 37% w/w and 2.3 ml of HNO_3_ 65% w/w) were added. After stirring, the beaker was covered with a watch glass and kept at room temperature for 16 h for pre-digestion. After this, the covered beaker was placed on the hot plate (90–100 °C) for 2 h. After cooling, the mixture was filtered, using JP-41 filter paper with 9 cm Ø (Quanty). The residue was washed with Milli-Q water, collecting the filtrate in a 50 ml volumetric flask. The filtrate was analysed by an atomic emission spectrometer with inductively coupled plasma (ICP-OES, Spectro/Ciros CCD) at the Laboratory of Geochemistry from the Federal University of Ouro Preto, Minas Gerais state, Brazil.

### Metals concentration in leaves

Leaf samples were dried at between 68 and 72 °C for 72 h and ground using stainless steel knife mill. To quantify metal concentrations (Al, Cu, Fe, Mn and Zn), 0.5 g of ground leaves were transferred to digestion tubes. After this, the samples were placed in the fume hood and 10 ml of HNO_3_ + HClO_4_ (4:1) were added. The samples were heated using a hot plate at 80 °C and the temperature was gradually increased until 200 °C. As soon as the extract became crystalline, the samples were removed from the plate to cool and their volumes were completed with 25 ml of demineralised water. Metals were quantified using an optical emission spectrometer with inductively coupled plasma (ICP-OES; Perkin Elmer Model Optima 8300 DV), at the Laboratory of Plant Tissue Analysis, from the Federal University of Viçosa, Minas Gerais State, Brazil. The equipment was calibrated with a multi-element solution in the same matrix as the samples at different concentrations.

### Statistical analyses

Data were analysed through model selection as suggested by Burnham and Anderson^78^. The R^79^ package MuMIn helped to identify models sharing substantial empirical evidence, that is (i) the best model—the model presenting lower AIC—and (ii) all the other equally probable models, which are those whose AIC differs less than 2 units from the best model. When needed for illustrative purposes the best model was chosen to build graphs. The use of the Second-Order Information Criterion (AICc) improved penalty to complex models built on a limited dataset. To keep ourselves at an acceptable level of reasoning complexity, we used the following strategy of model interpretation: (i) when inspecting the biological importance of explanatory variables (herbivory and metal content in the leaves) in general, we refer to all models sharing substantial empirical evidence; (ii) when interpreting the biological effects of a given model term in particular, we restrained ourselves to the best model only.

To confirm whether or not that metal accumulations affected plant growth, the following global model was applied to the data: growth_rate1 ~ (Al + Cu + Fe + Mn + Zn), in which growth_rate1 is the plant growth affected only by metals (that is, the candeias’ growth rate from the beginning of the greenhouse bioassay until before they were taken to the field; see M&M and Fig. 3) and Al, Cu, Fe, Mn and Zn are these metal concentrations in candeias’ leaves.

To test the hypothesis that metal accumulations affected the percentage of leaf area lost by candeias as a consequence of herbivory, the following global model was applied: perc_cons_area ~ (Al + Cu + Fe + Mn + Zn), in which perc_cons_area is the percent of the total leaf area that was consumed by herbivores after 4 months of the field experiment.

To test the hypothesis that metal accumulation in plants tissue and herbivory can affect plant growth, the following global model was applied: growth_rate ~ (Al + Cu + Fe + Mn + Zn) * atc_lvs, in which growth_rate is the growth of plants affected by metals and herbivory (that is, the growth rate of the plants after they were grown on heavy metal soils in the greenhouse and then submitted to the herbivores in the field), Al, Cu, Fe, Mn, Zn are these metal concentrations in candeia’s leaves and atc_lvs is the number of leaves presenting any sign of herbivore attack after 4 months of the field experiment. The asterisk (*) indicates statistical interactions between metal concentrations and herbivory levels.

## Supplementary Information

Supplementary Information 1.

Supplementary Information 2.

Supplementary Information 3.

## Data Availability

The data generated and analysed during this study are included in this published article (and its Supplementary Information files). The images generated and analysed during the current study are available in the Zenodo repository, https://zenodo.org/record/4642115#.YF_ABRLQ-f8.
