# Supplementary material for: Metal bioaccumulation alleviates the negative effects of herbivory on plant growth
Source: Sci Rep. 2021 Sep 24;11:19062. doi: 10.1038/s41598-021-98483-x (PMC8463685; doi:10.1038/s41598-021-98483-x)
Supplement: Supplementary file 1 — Supplementary Information 1. [file 41598_2021_98483_MOESM1_ESM.pdf]

Article title: Metal bioaccumulation reverting from negative to positive the effects of herbivores on plant growth

Author names: Grazieli F Dueli, Og DeSouza, Servio P Ribeiro

Affiliation: Universidade Federal de Viçosa

E-mail: grazidueli@gmail.com

Figure S1: Scanned image of leaves from 68 Candeias, *Eremanthus erythropappus* DC. MacLeish (Asteraceae), that grown on soils with metal concentration gradient (Al, Cu, Fe, Mn, Zn). The plants were taken to the field, where they have been exposed to herbivory for four months. To measure the lost leaf area, 20 leaves of each plant were randomly sorted and scanned. From the images, the percentages of lost leaf area were calculated using Sigma Scan software.

The scale of the images 23, 49a, 49b, 53a, 5b is in centimeters. The scale of the other images is in inches.

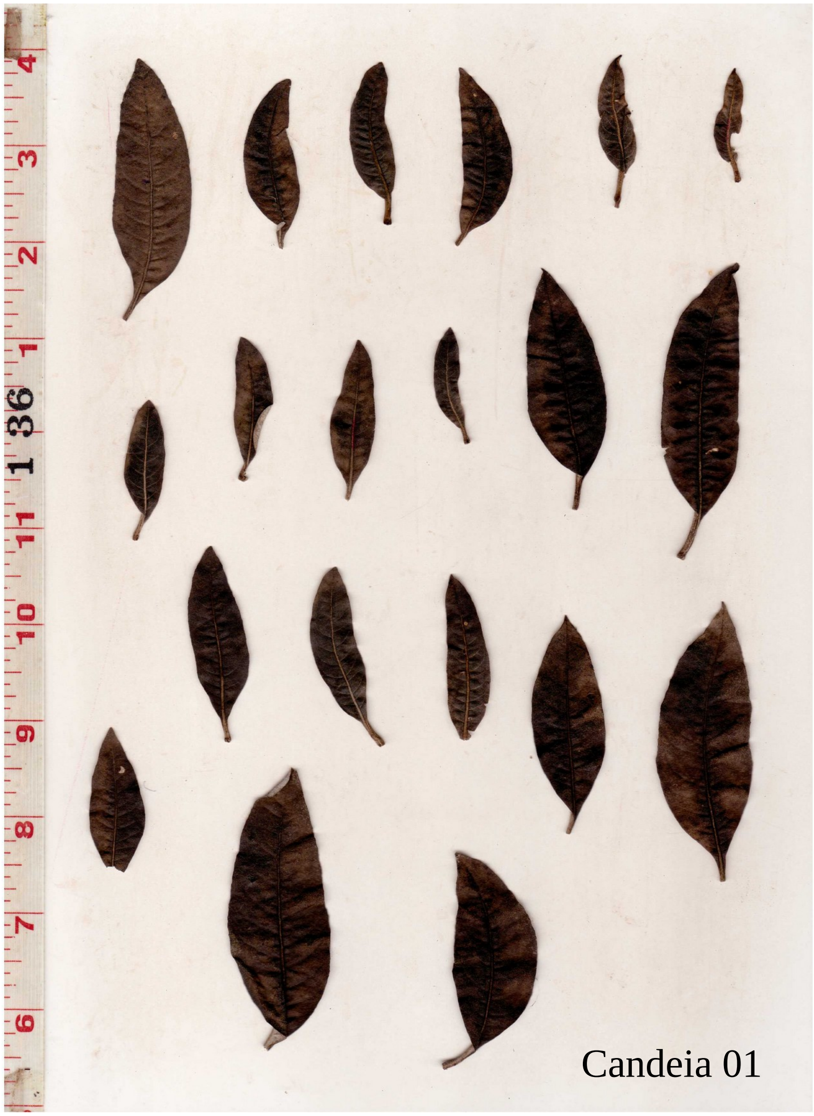

Candeia 01

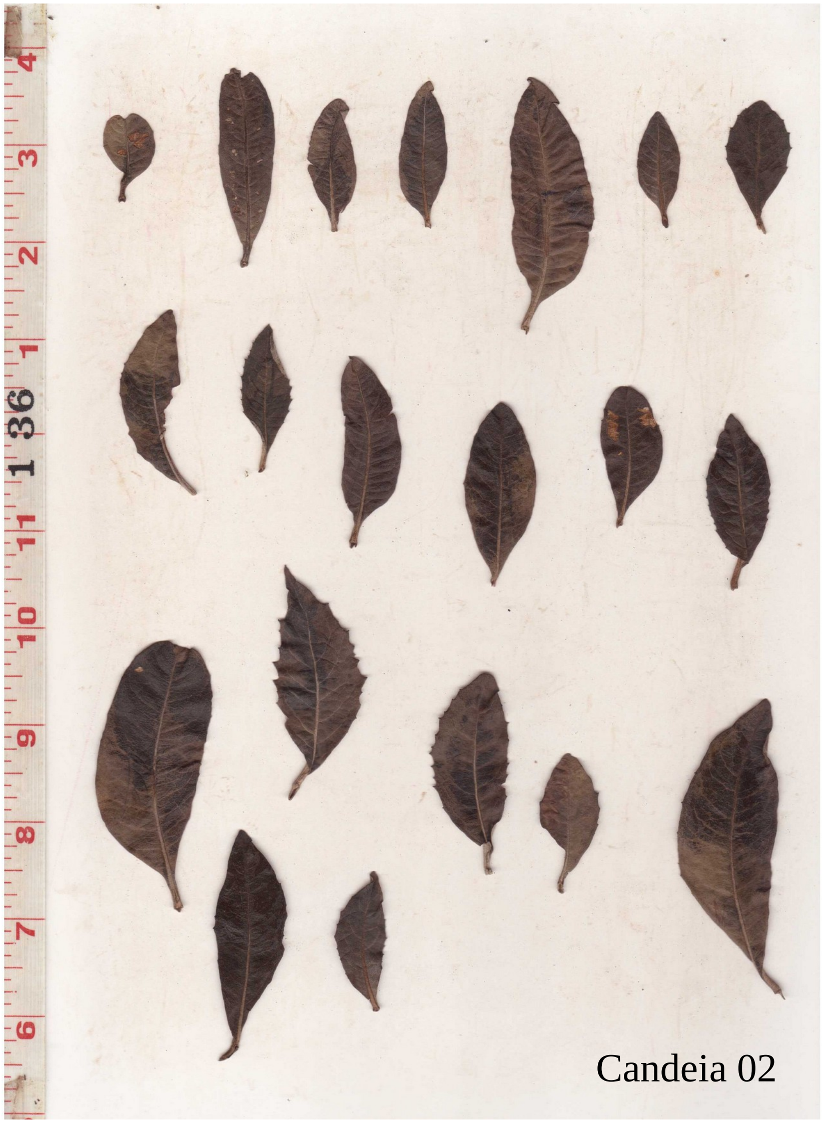

Candeia 02

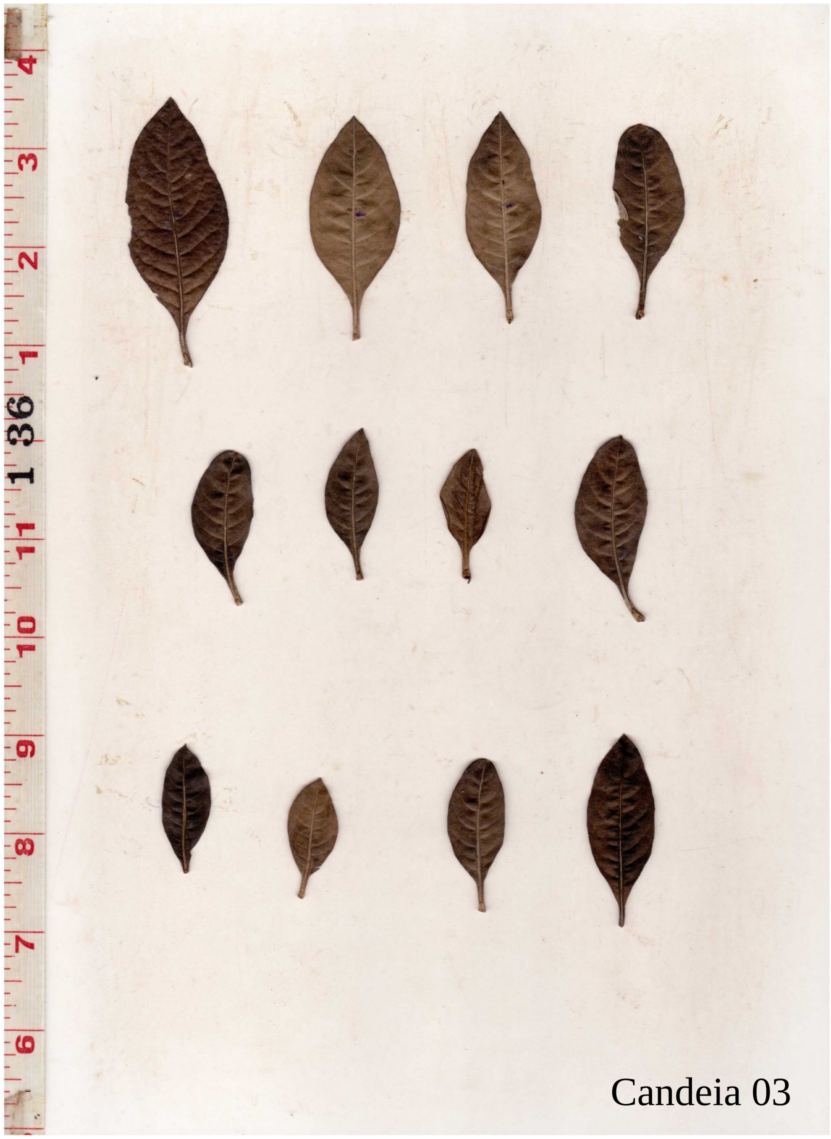

Candeia 03

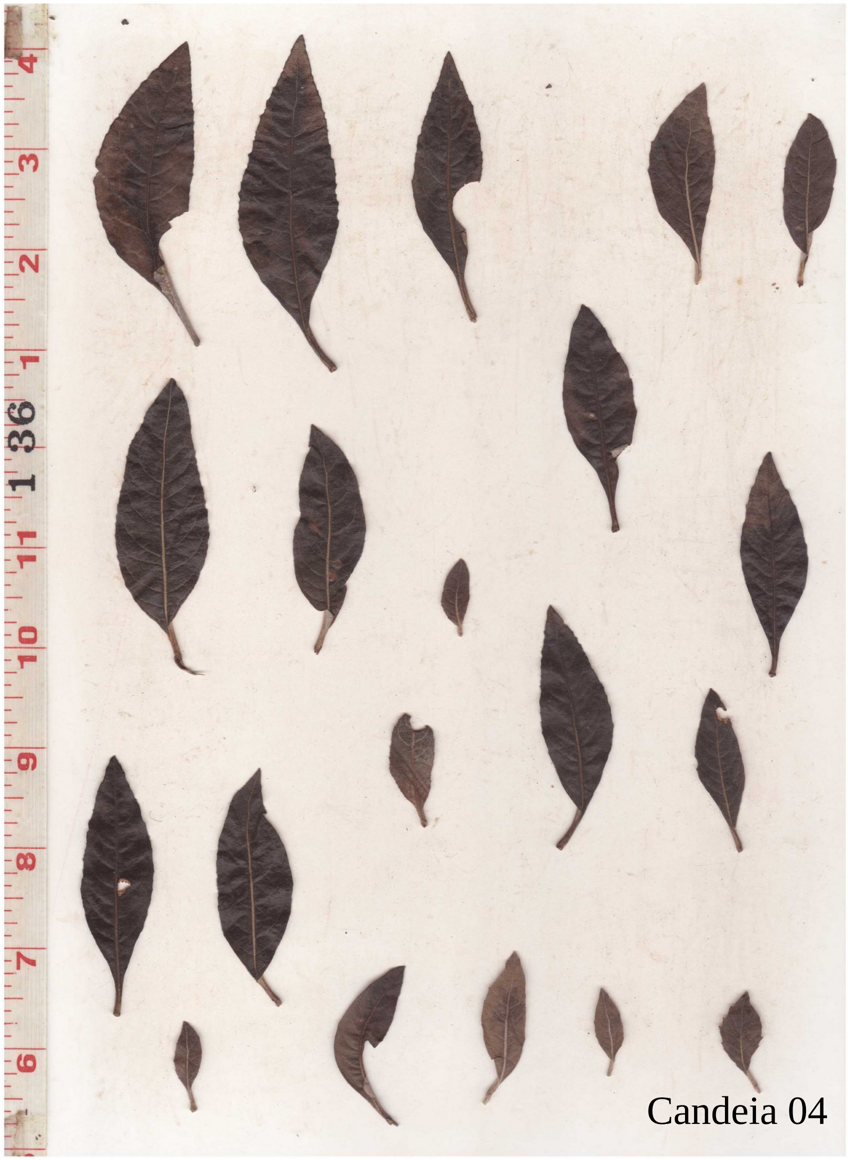

Candeia 04

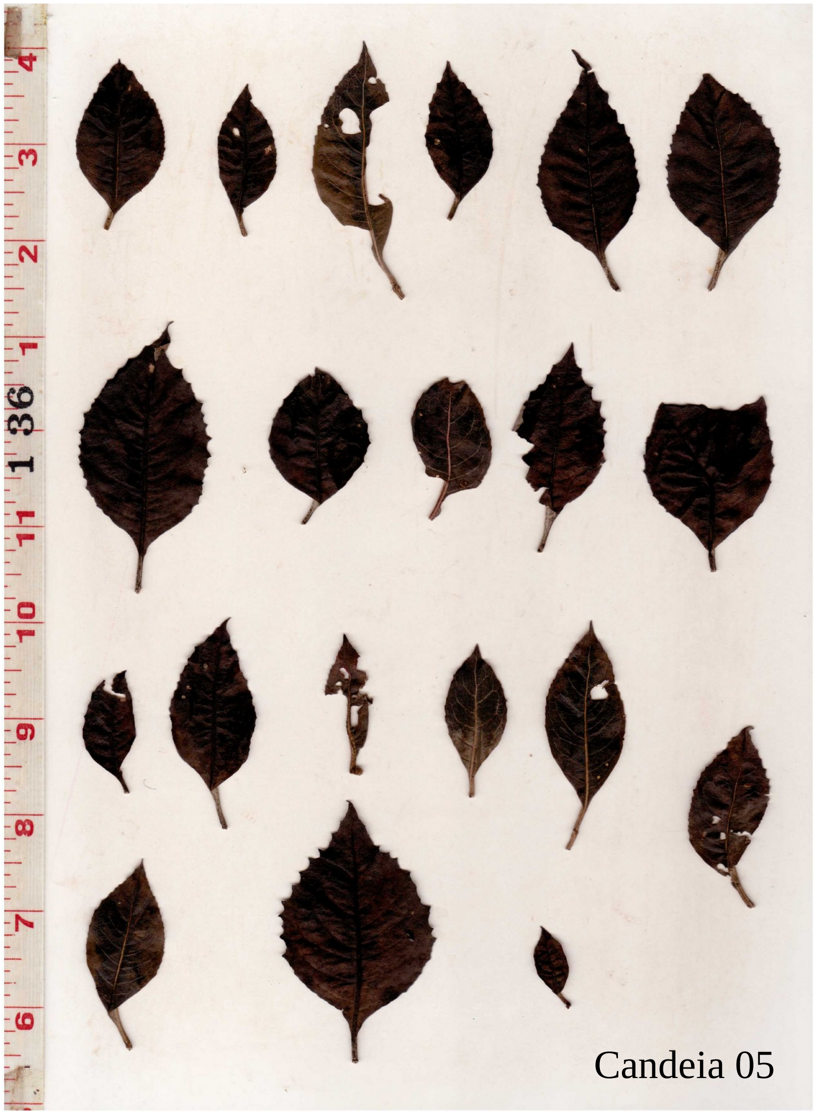
Candeia 05

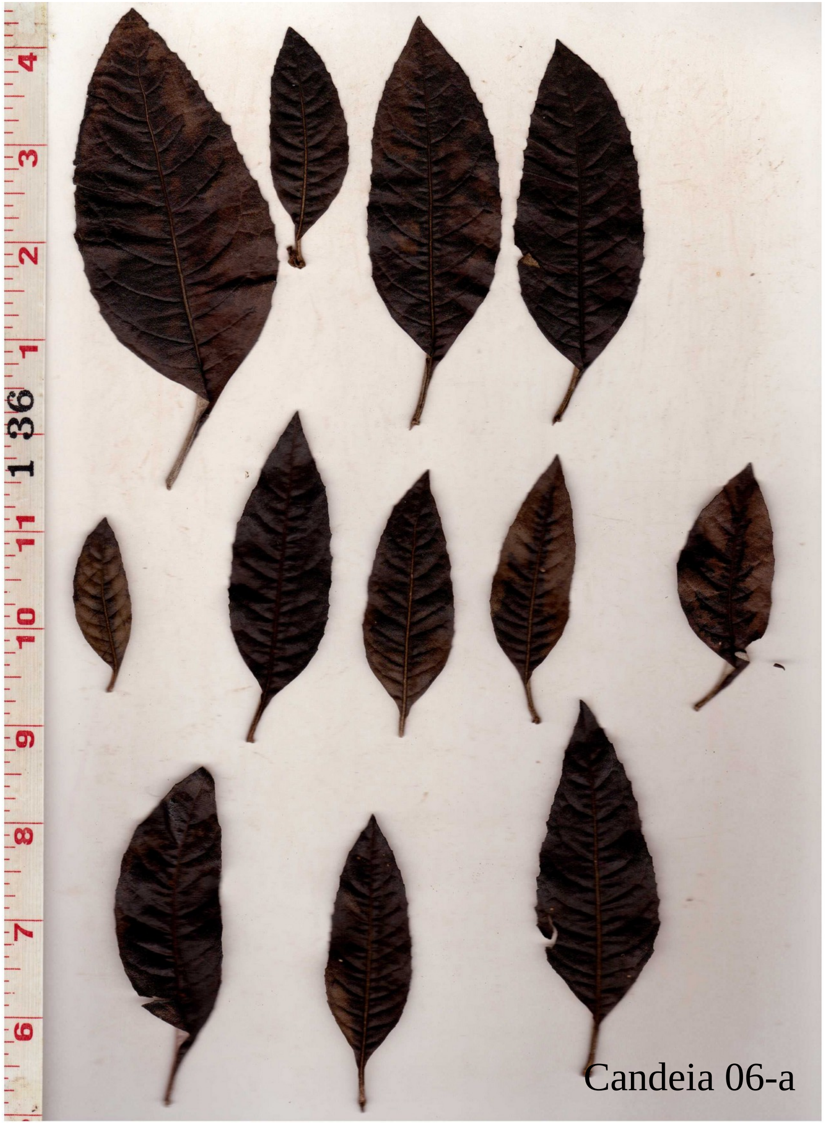
Candeia 06-a

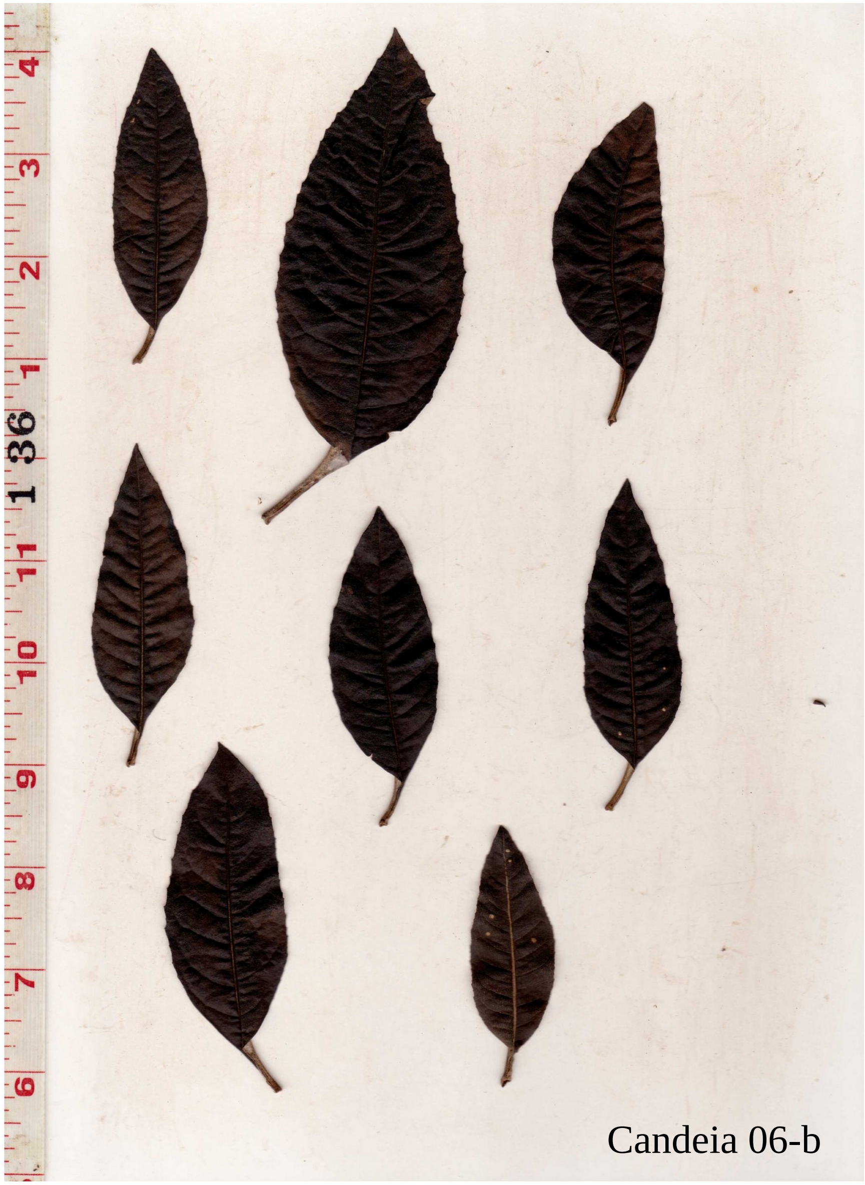
Candeia 06-b

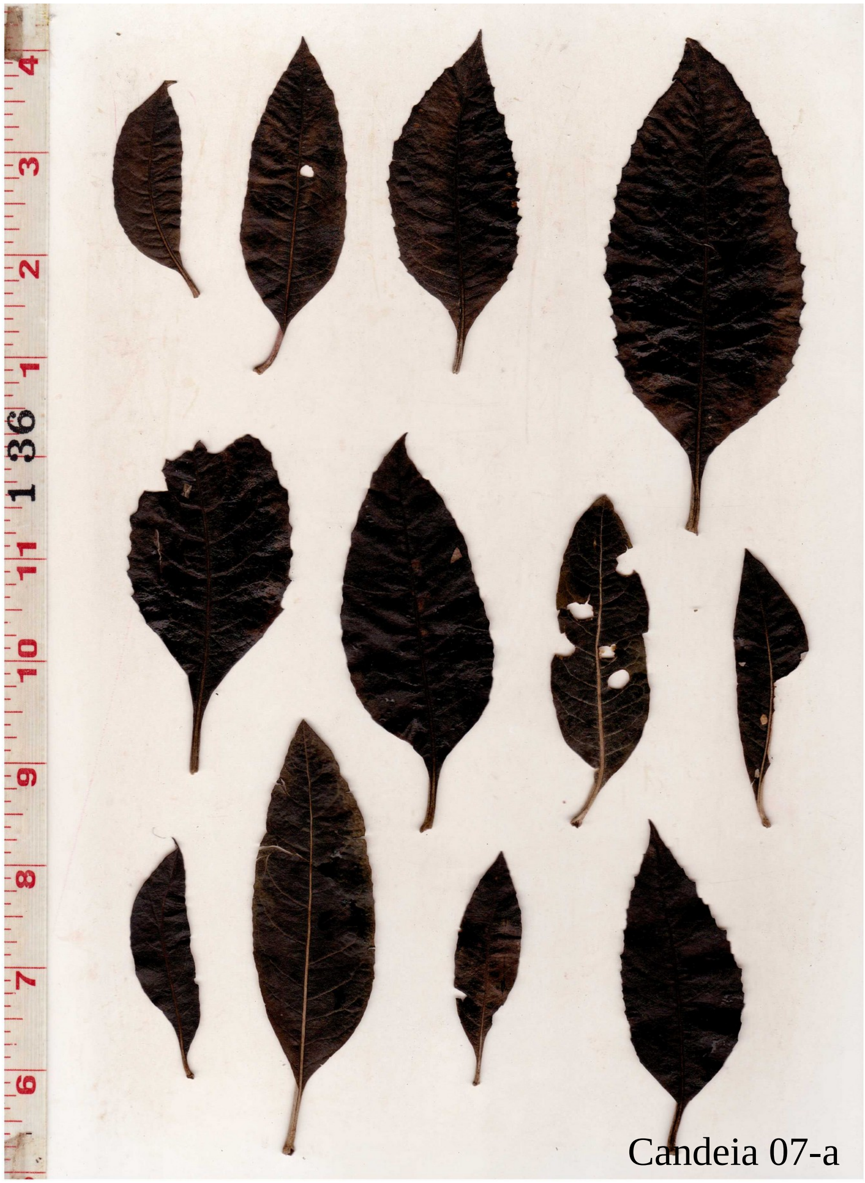
Candeia 07-a

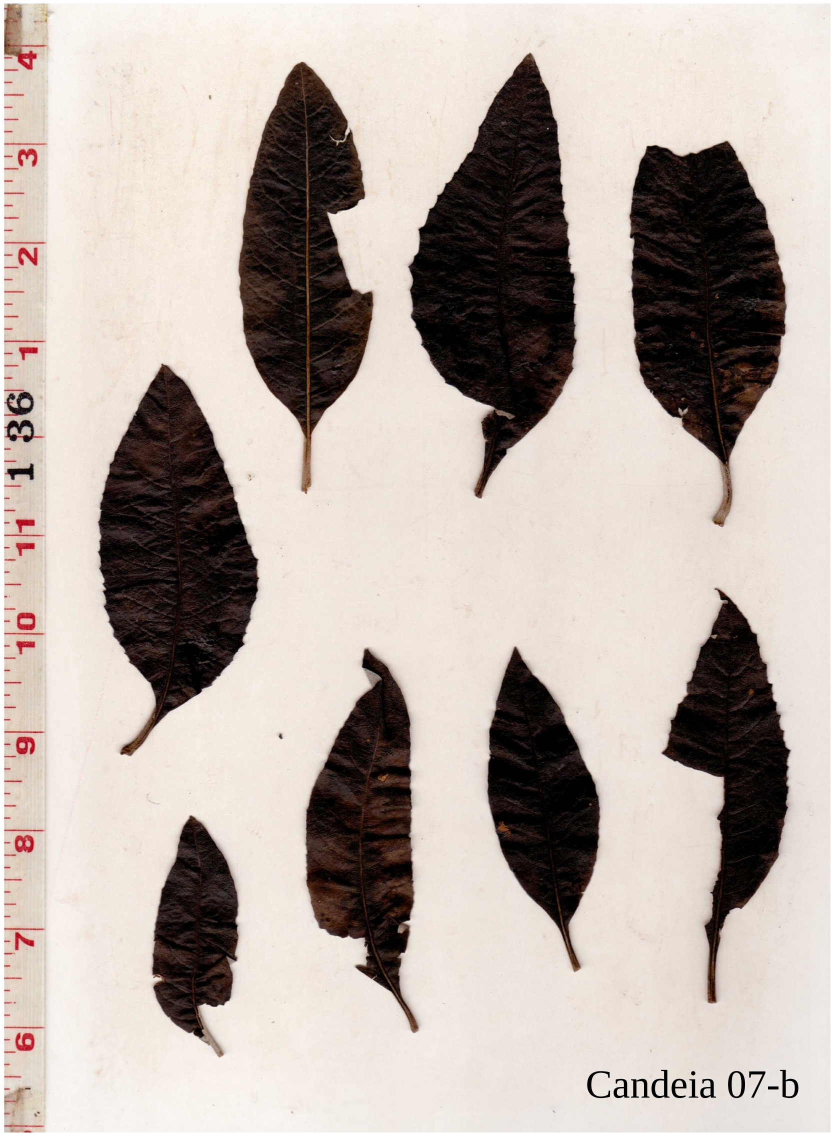

Candeia 07-b

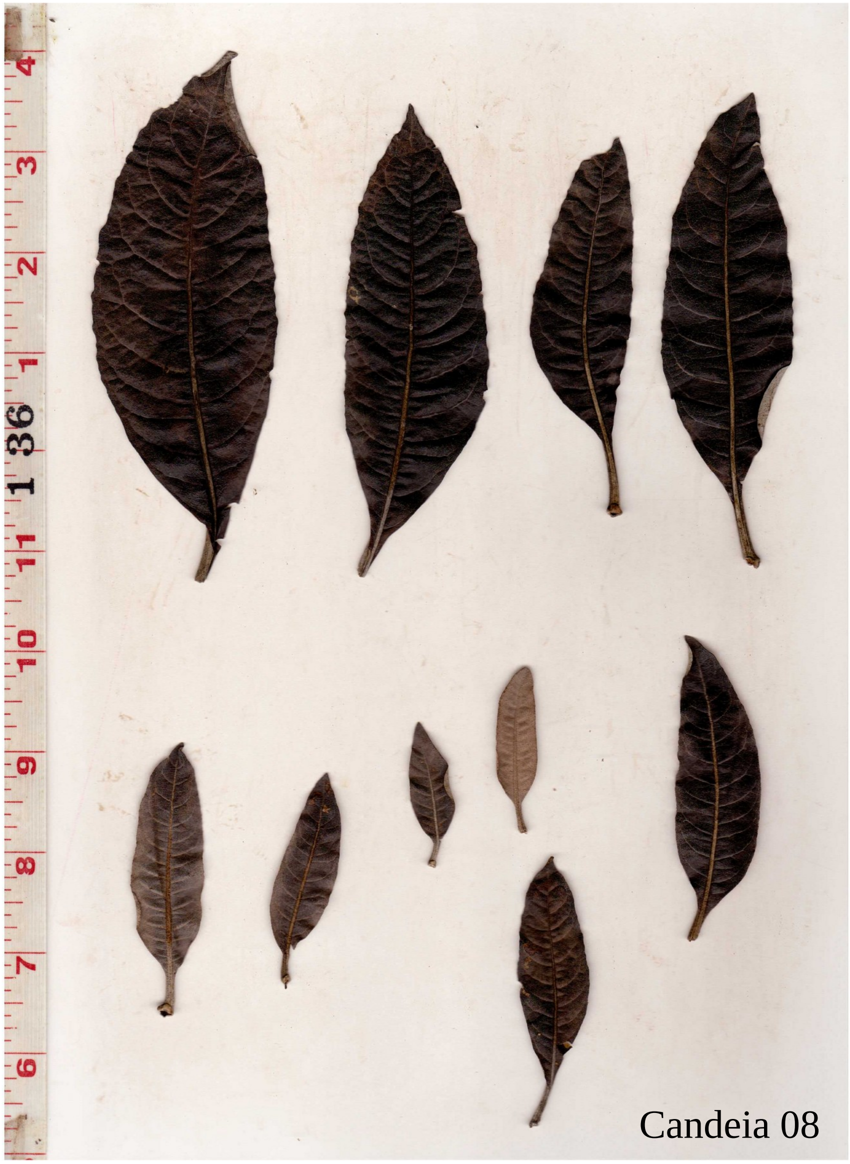

Candeia 08

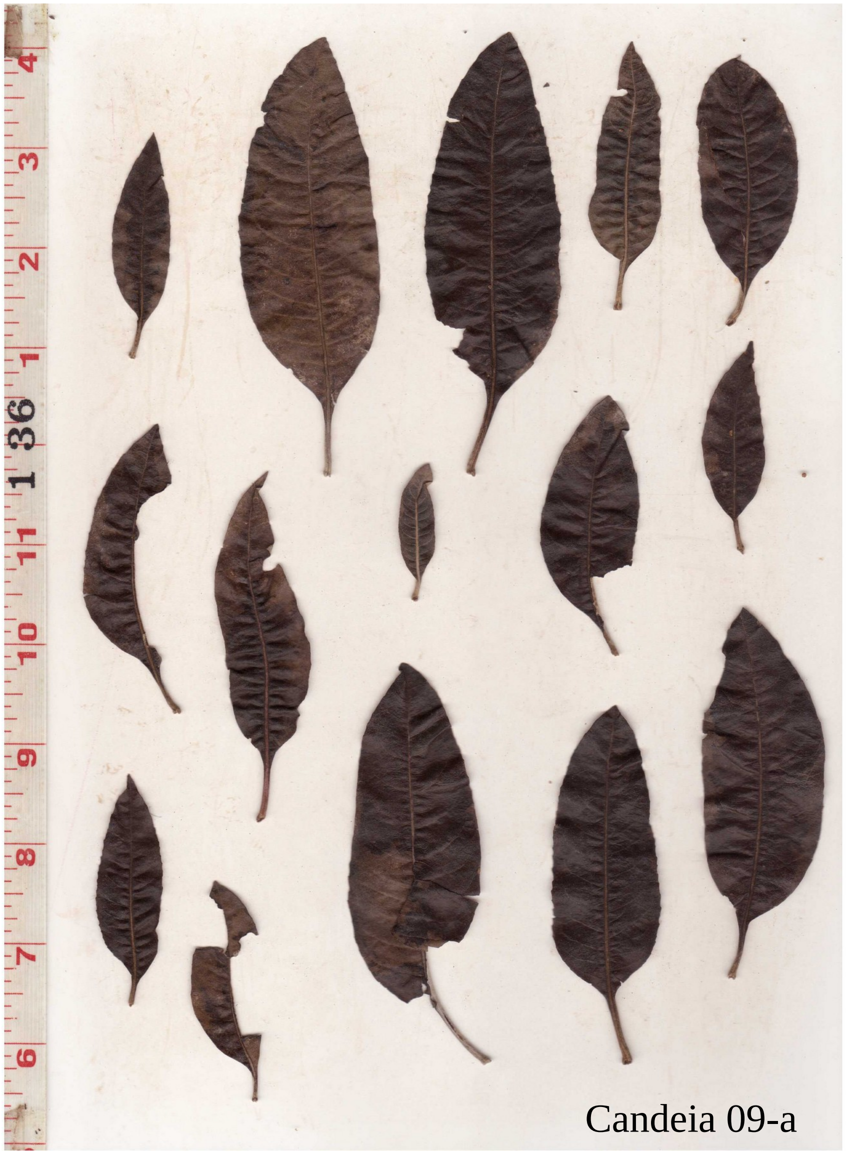
Candeia 09-a

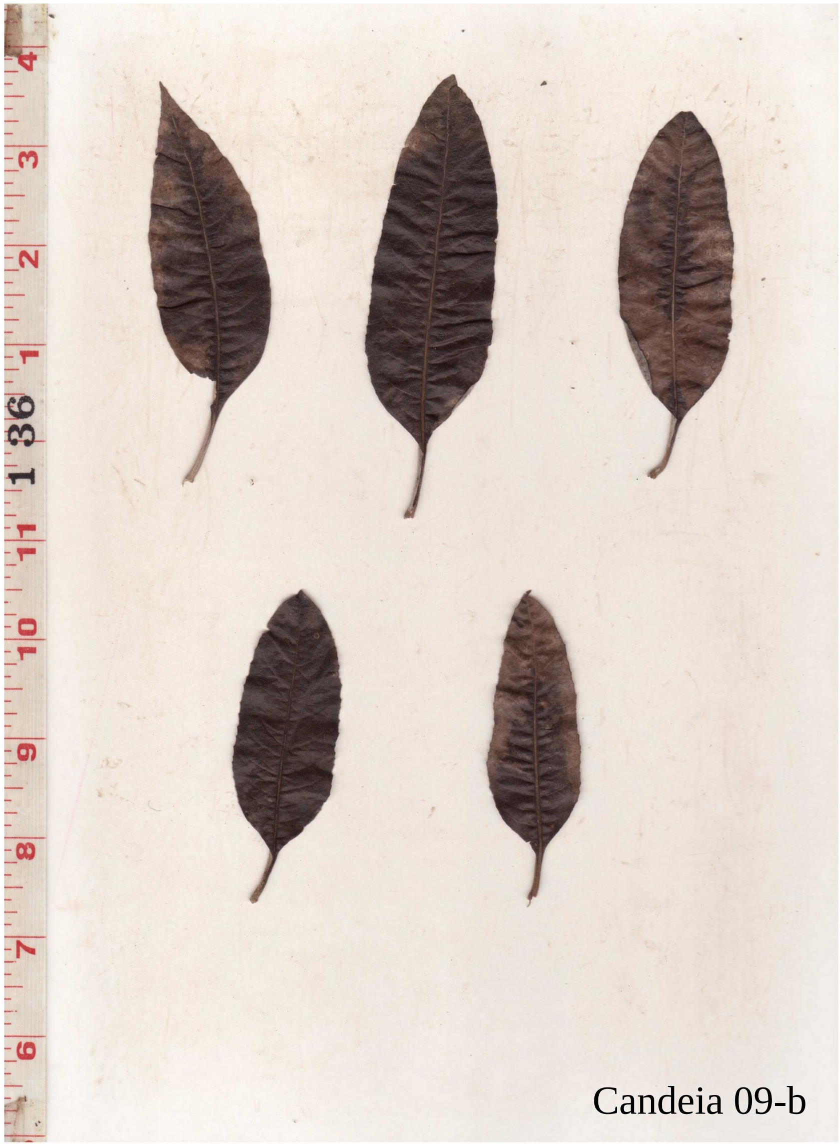
Candeia 09-b

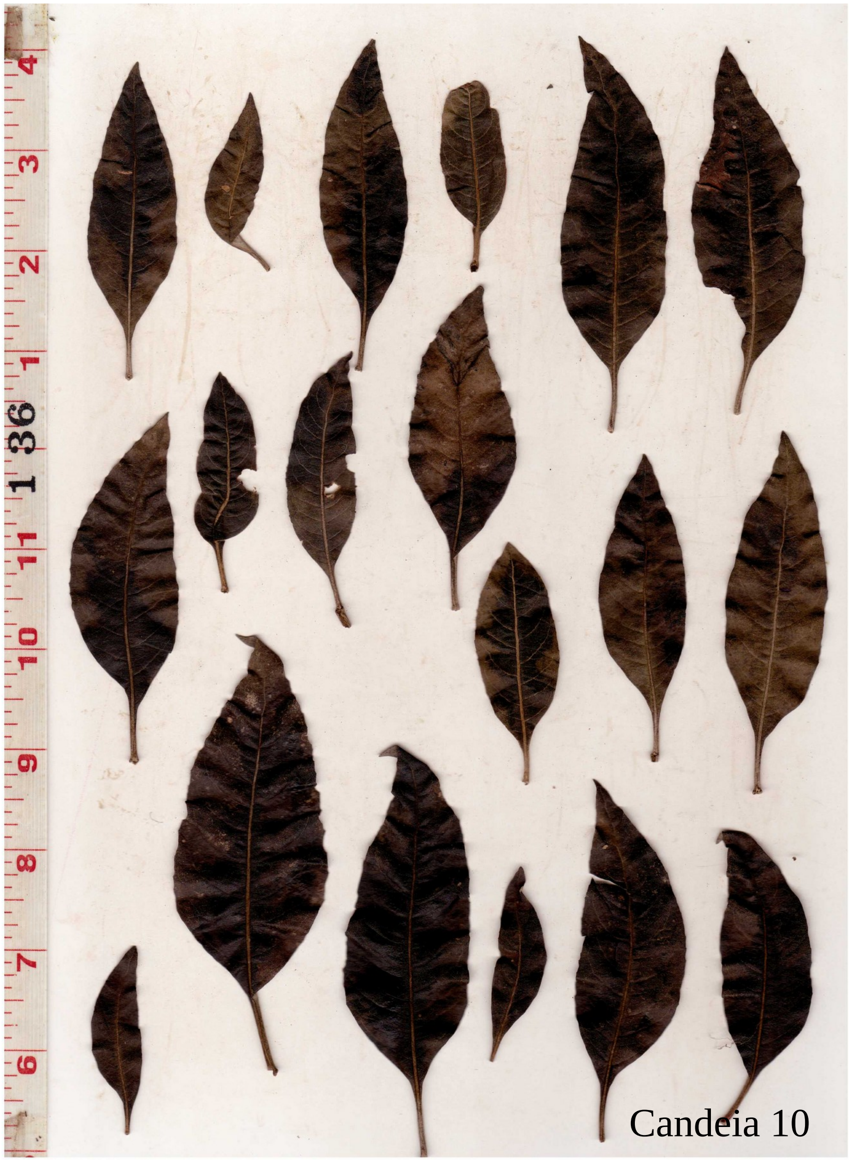

Candeia 10

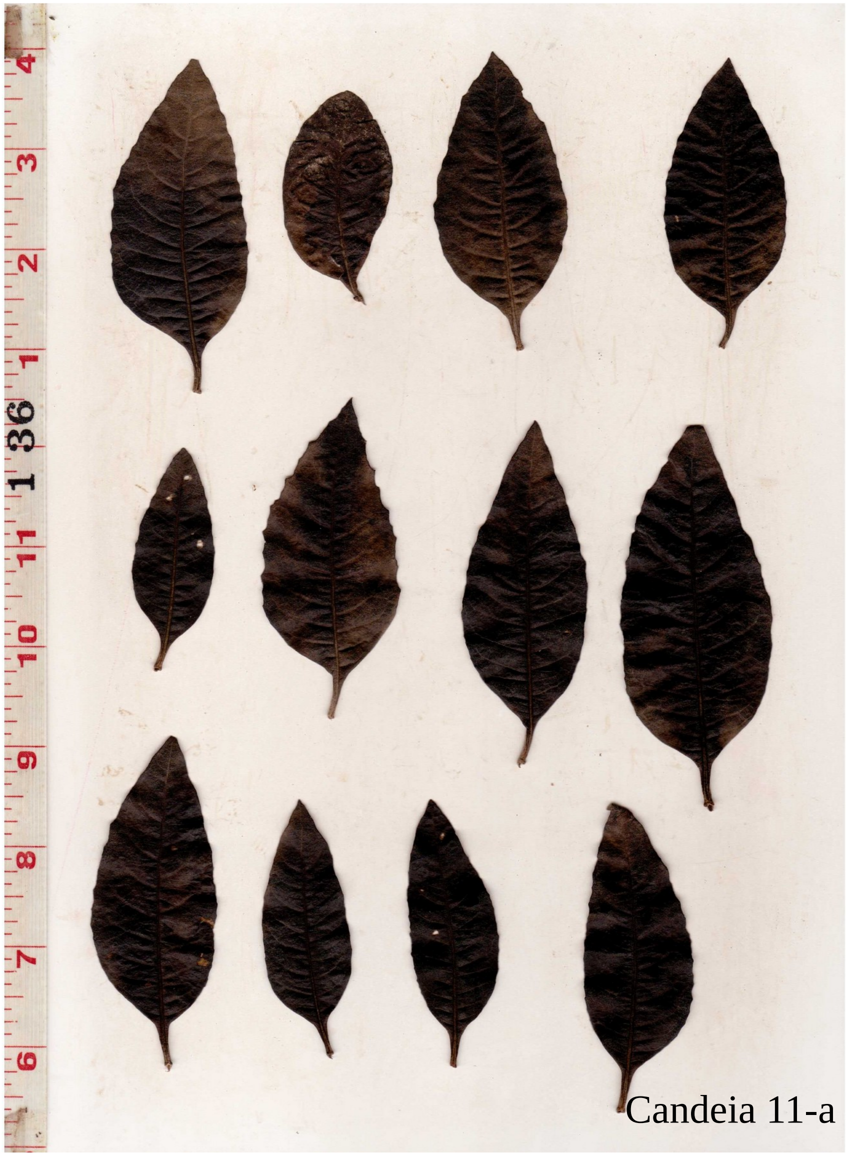

Candeia 11-a

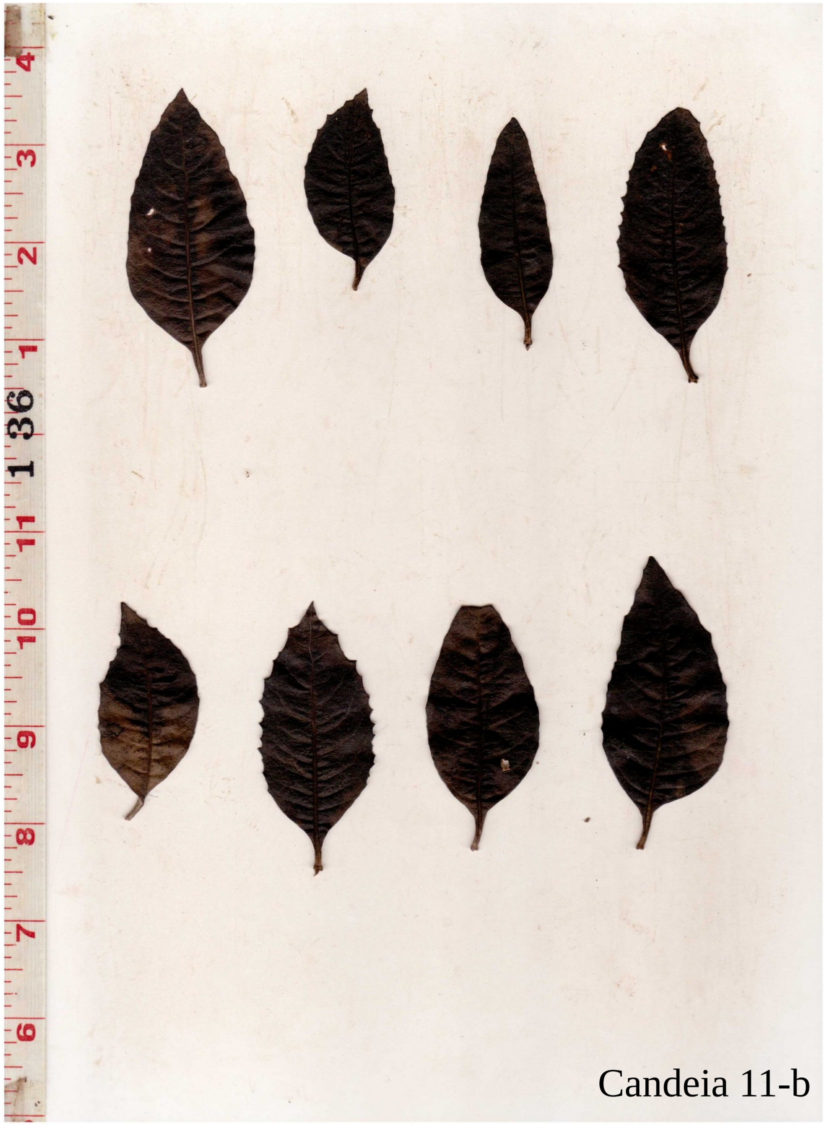

Candeia 11-b

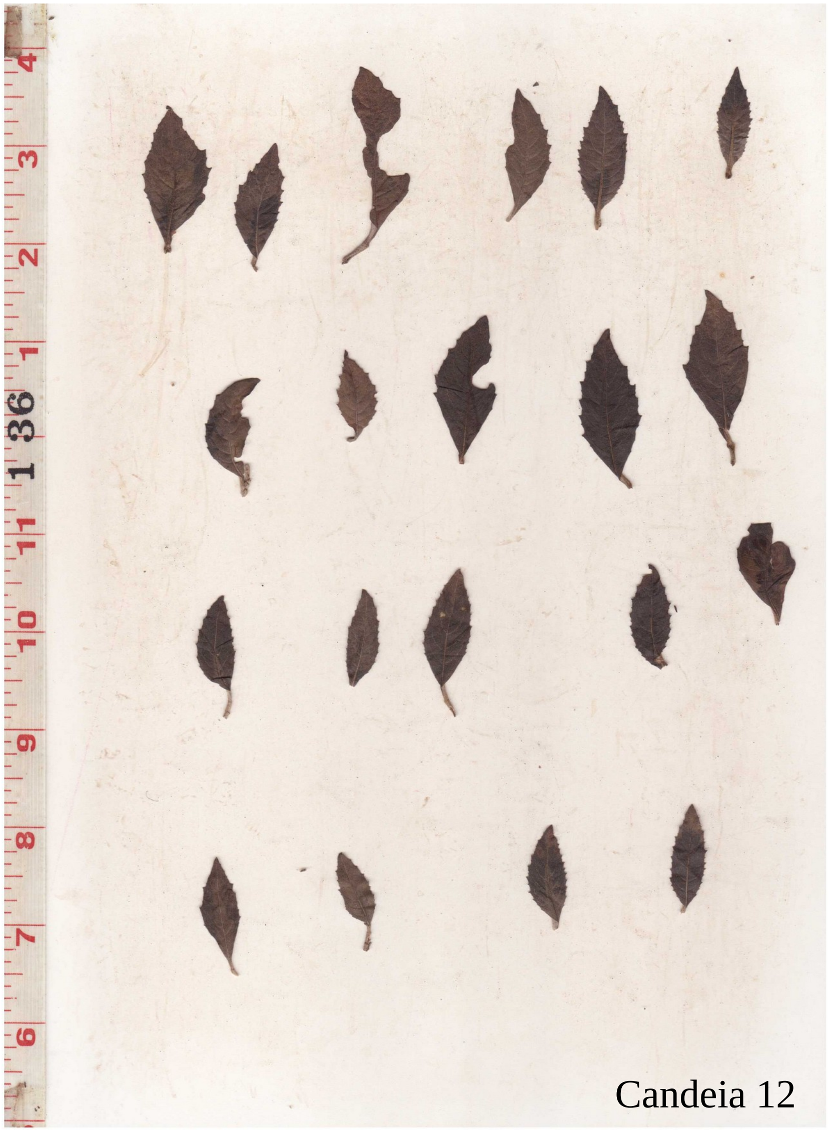

Candeia 12

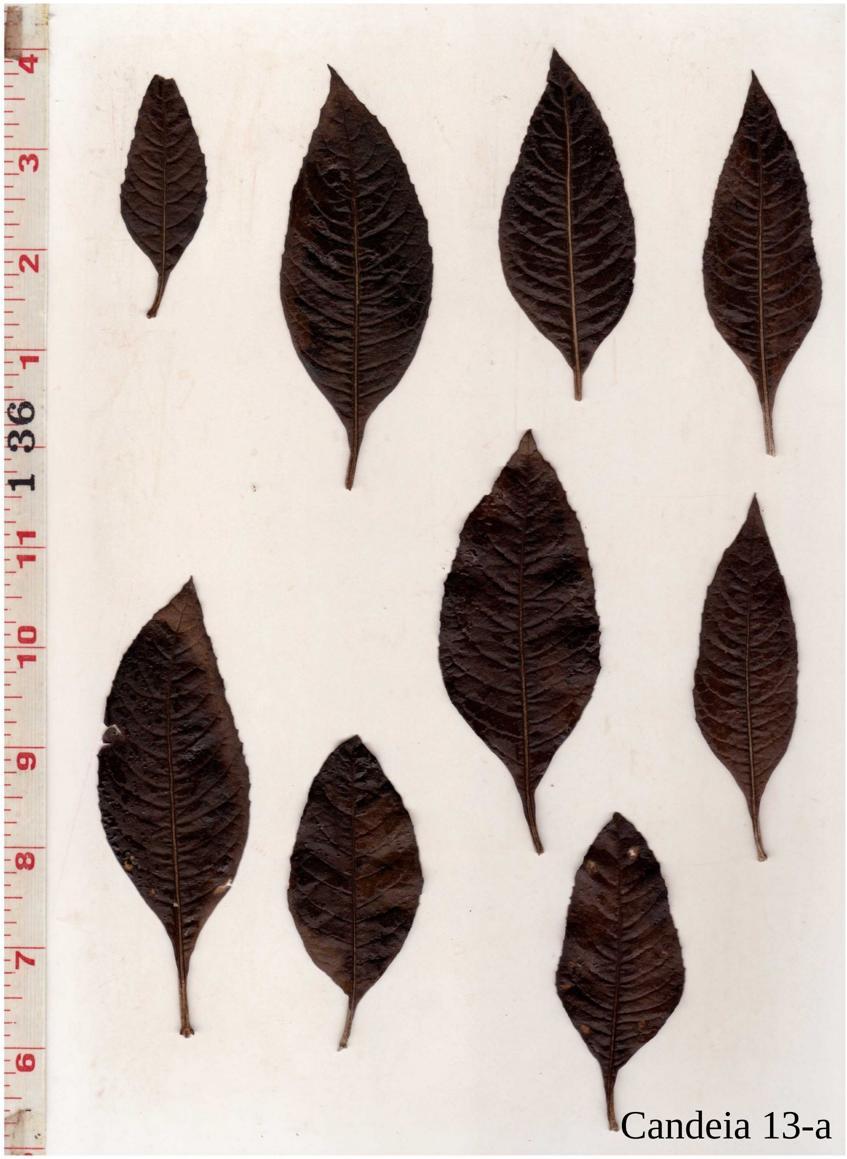
Candeia 13-a

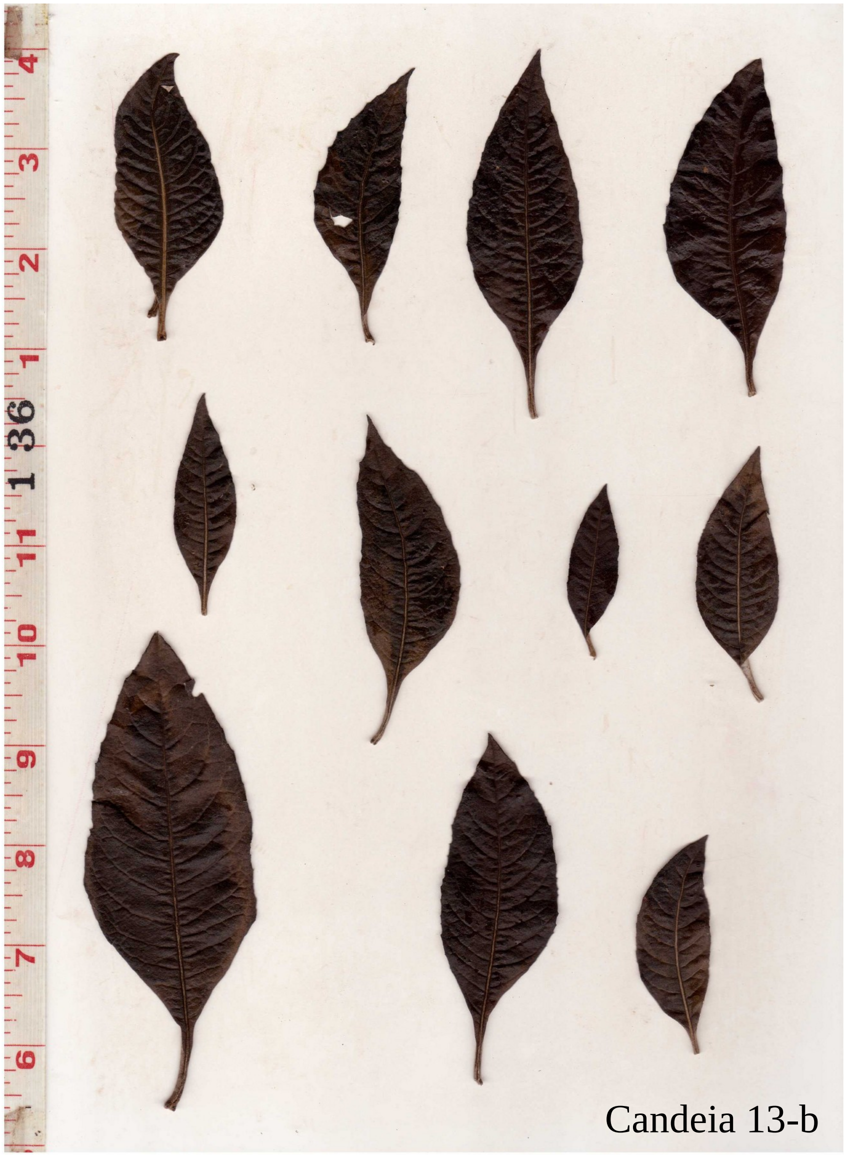
Candeia 13-b

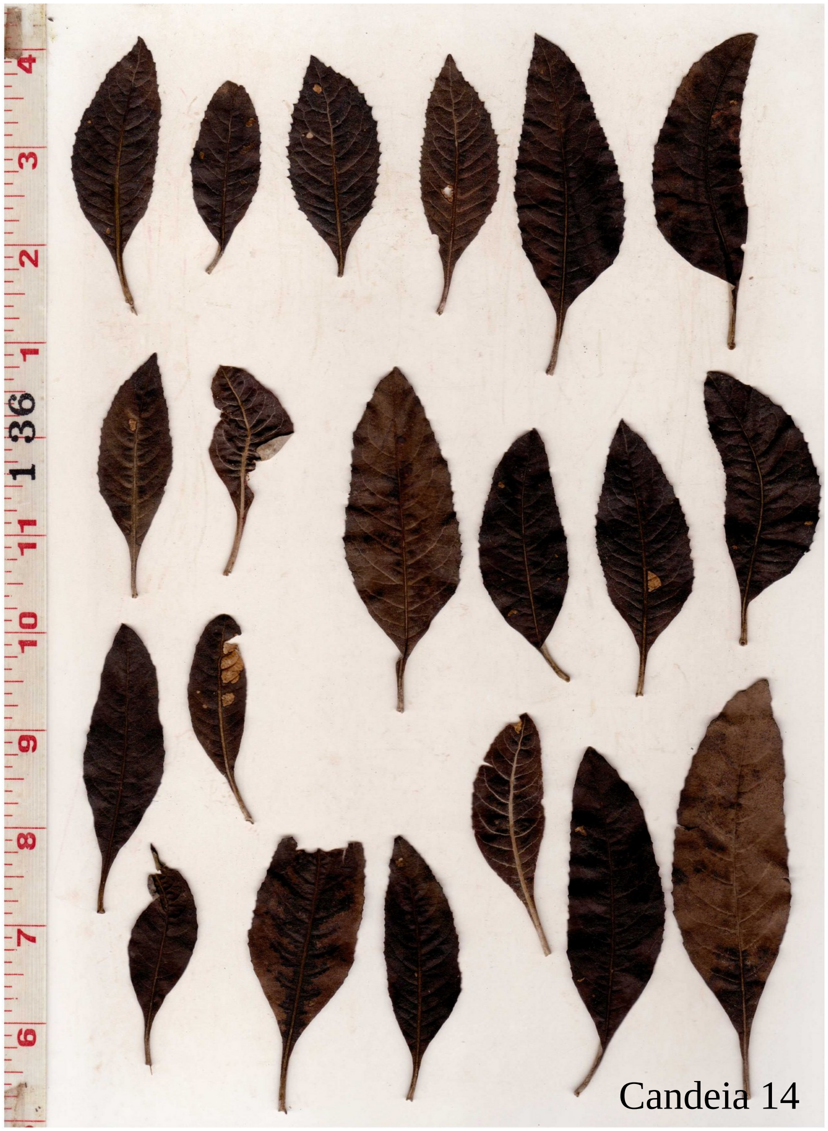
Candeia 14

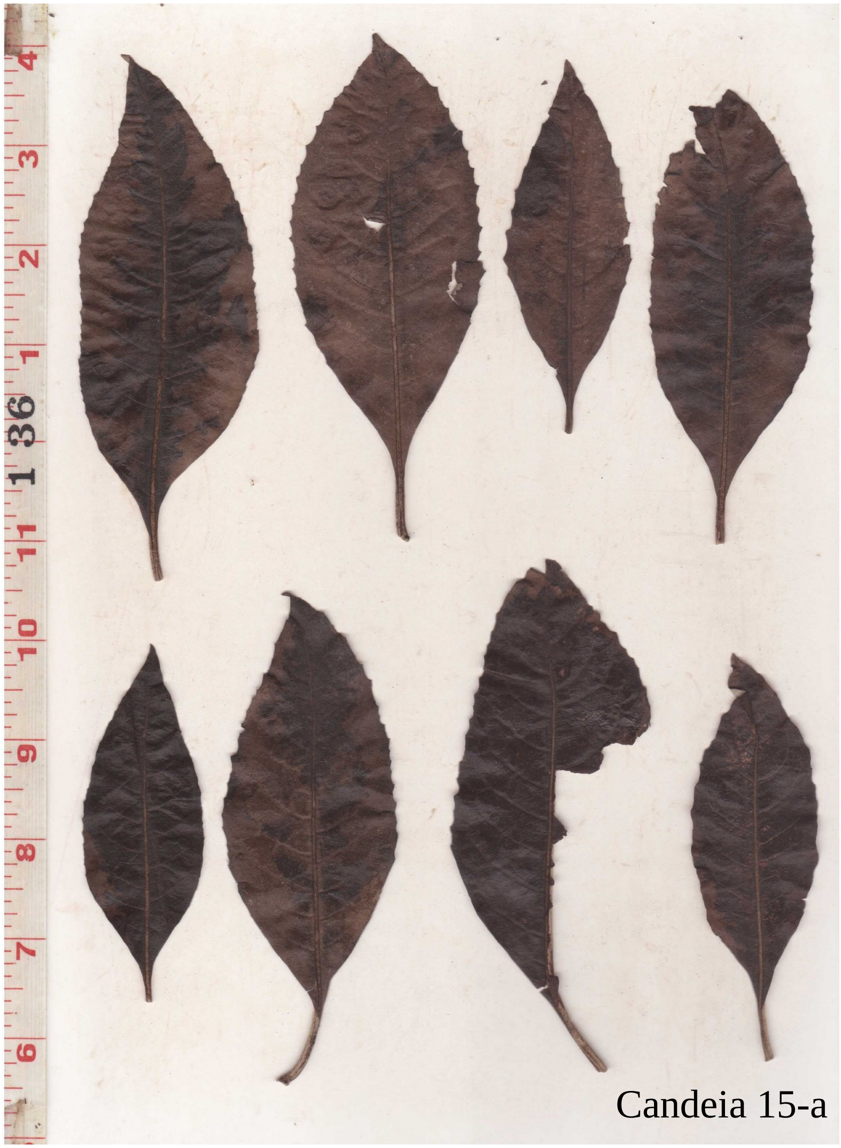
Candeia 15-a

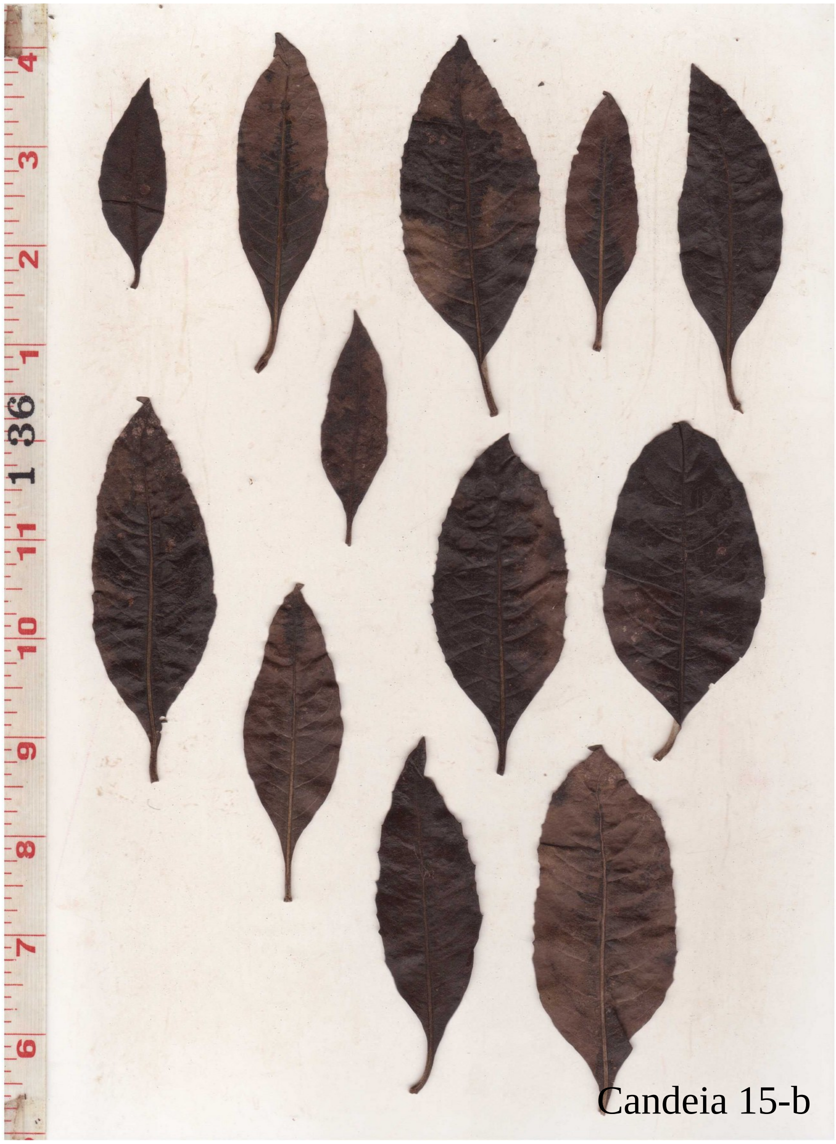
Candeia 15-b

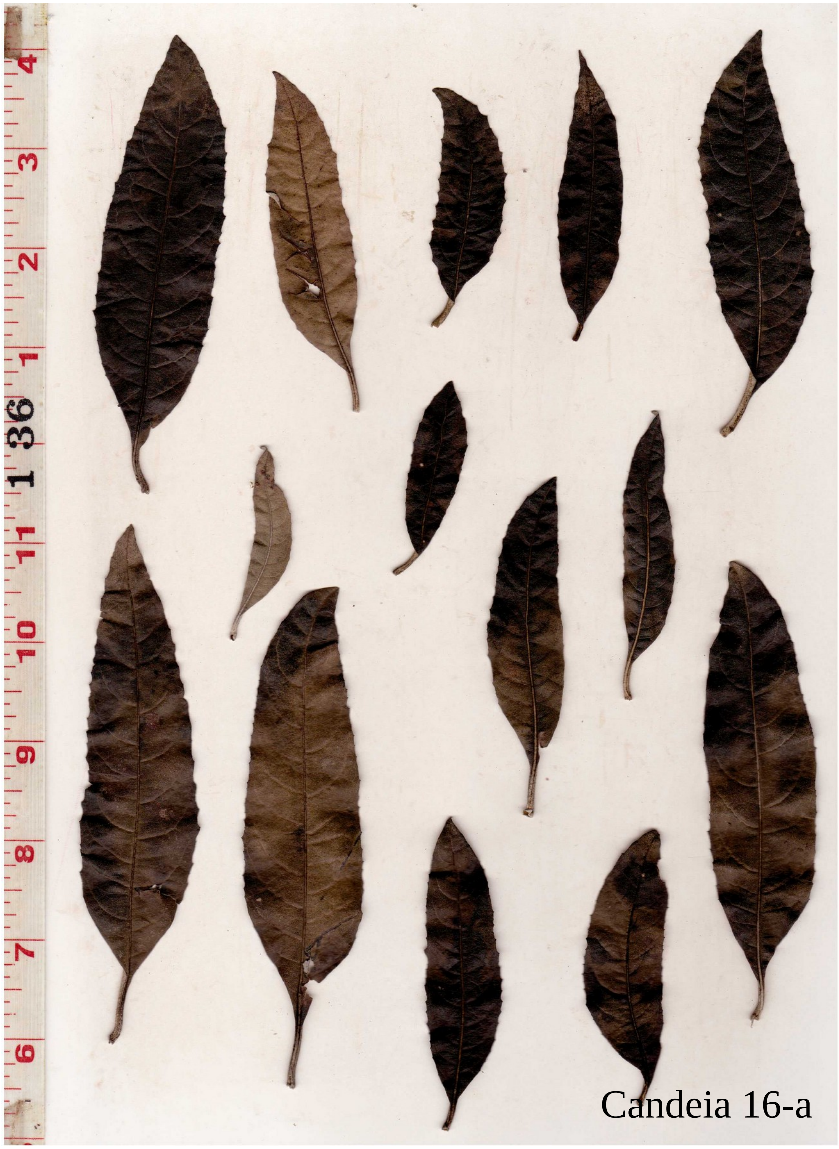
Candeia 16-a

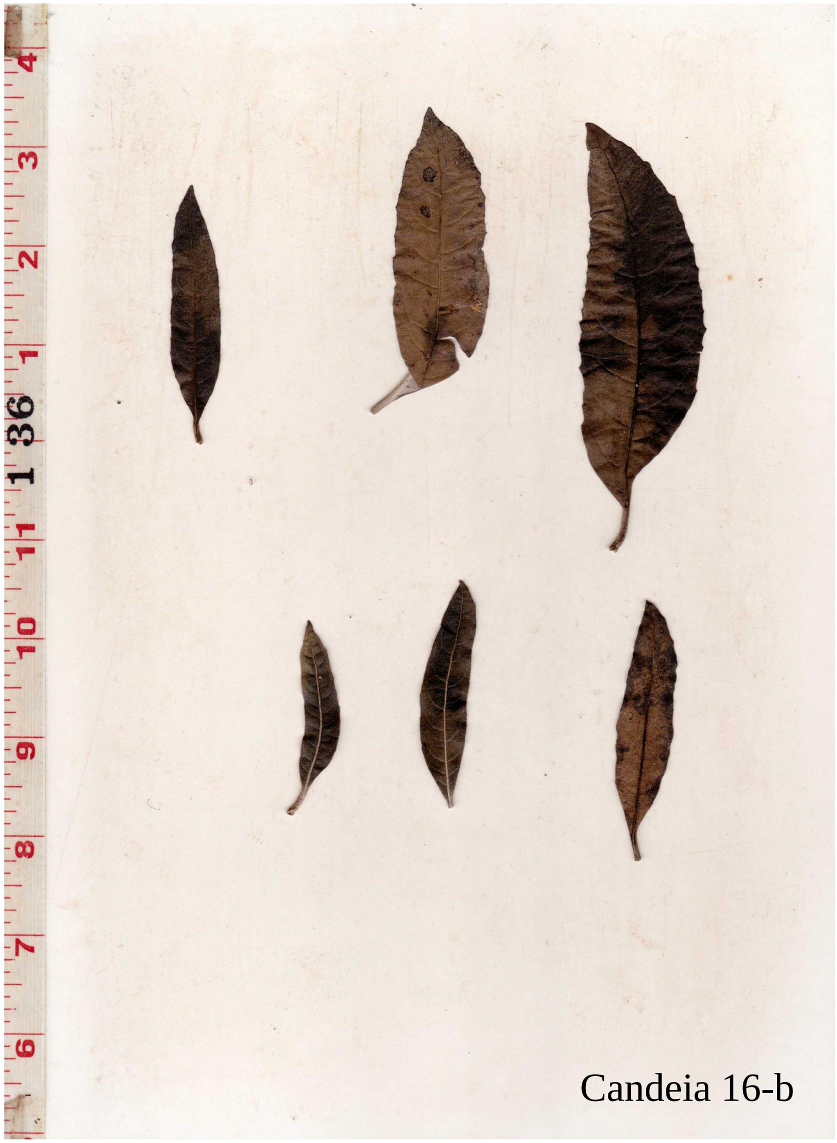

Candeia 16-b

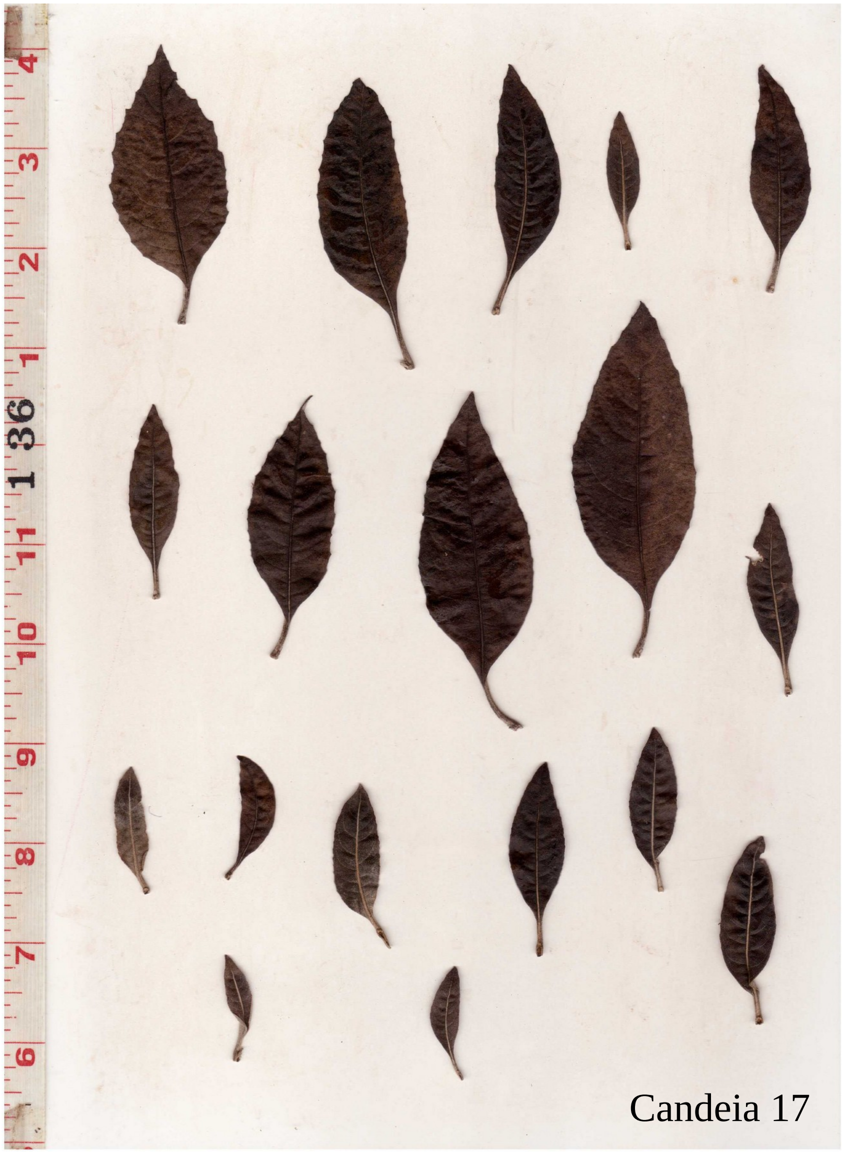

Candeia 17

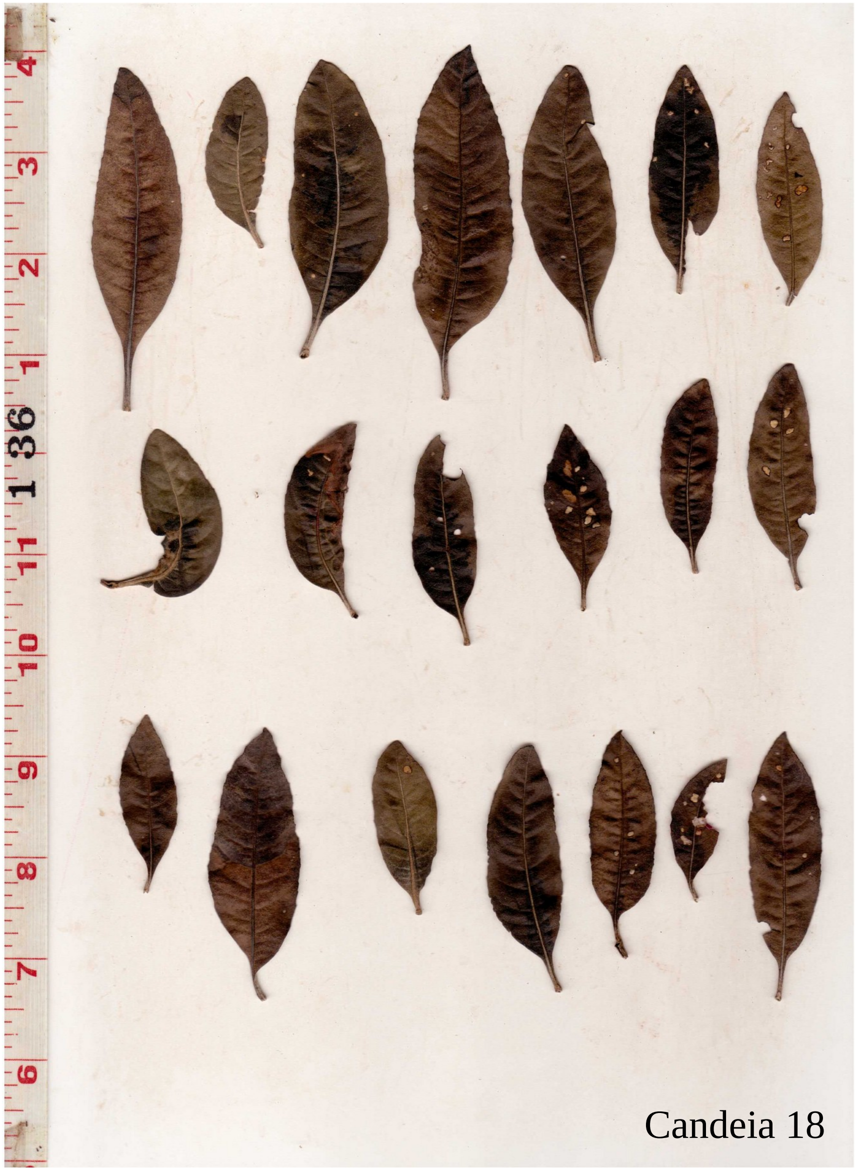
Candeia 18

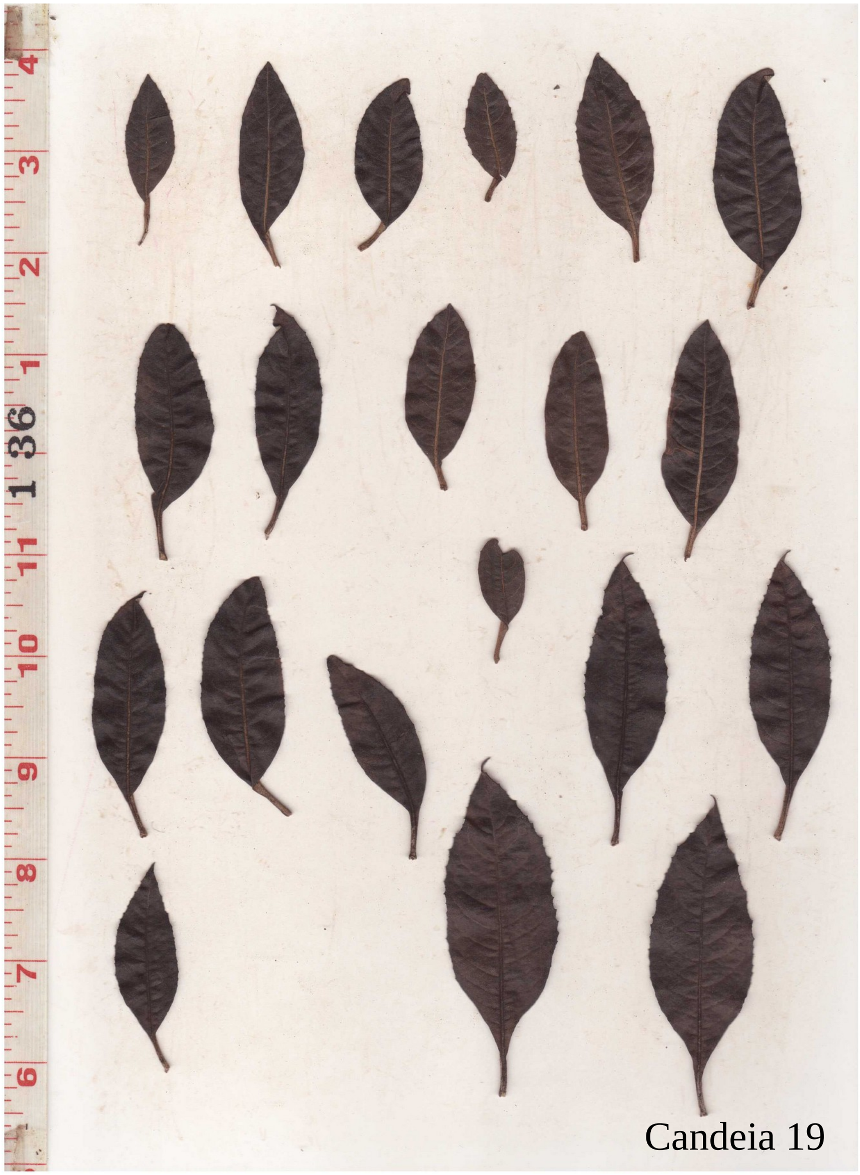
Candeia 19

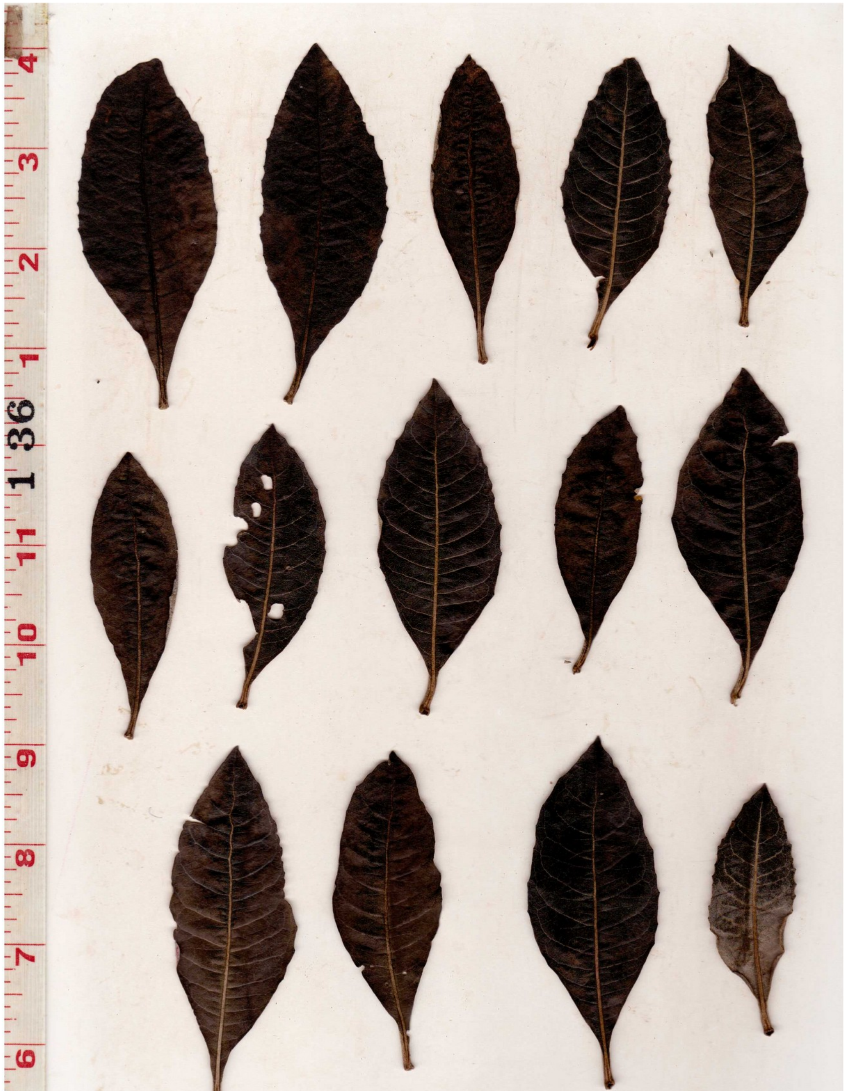

Candeia 20-a

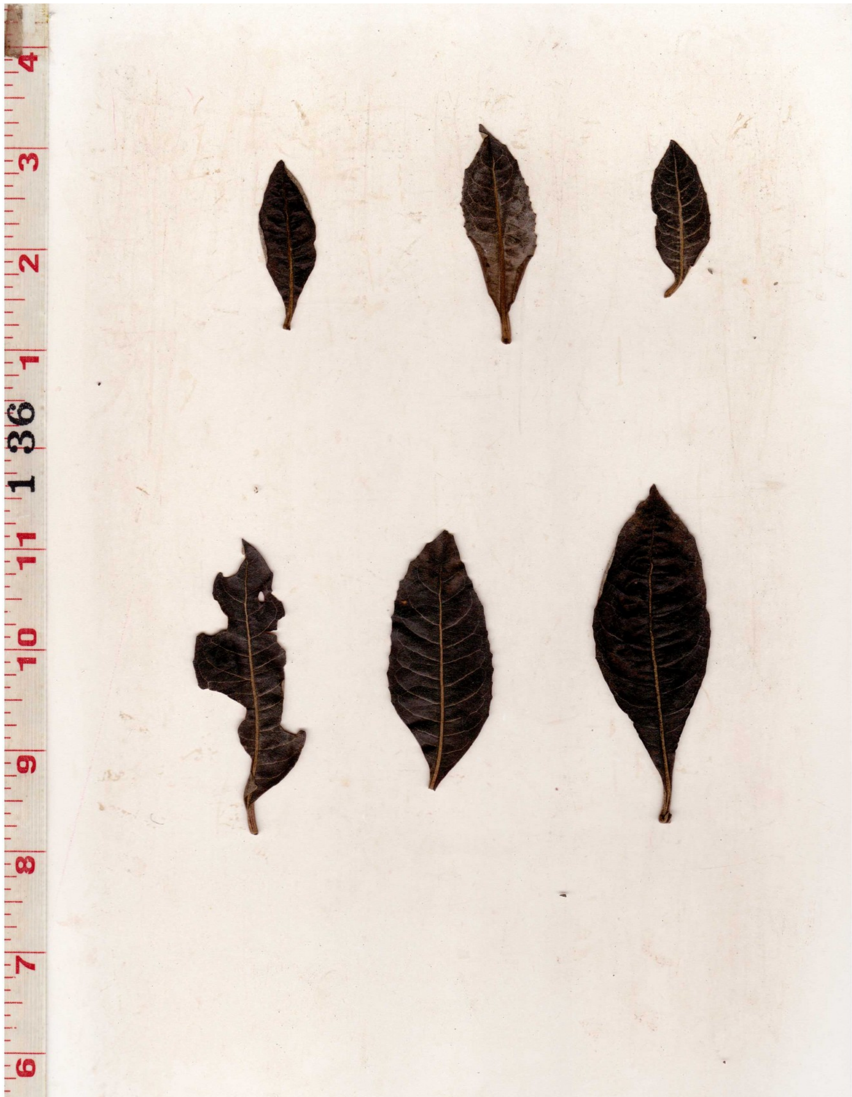

Candeia 20-b

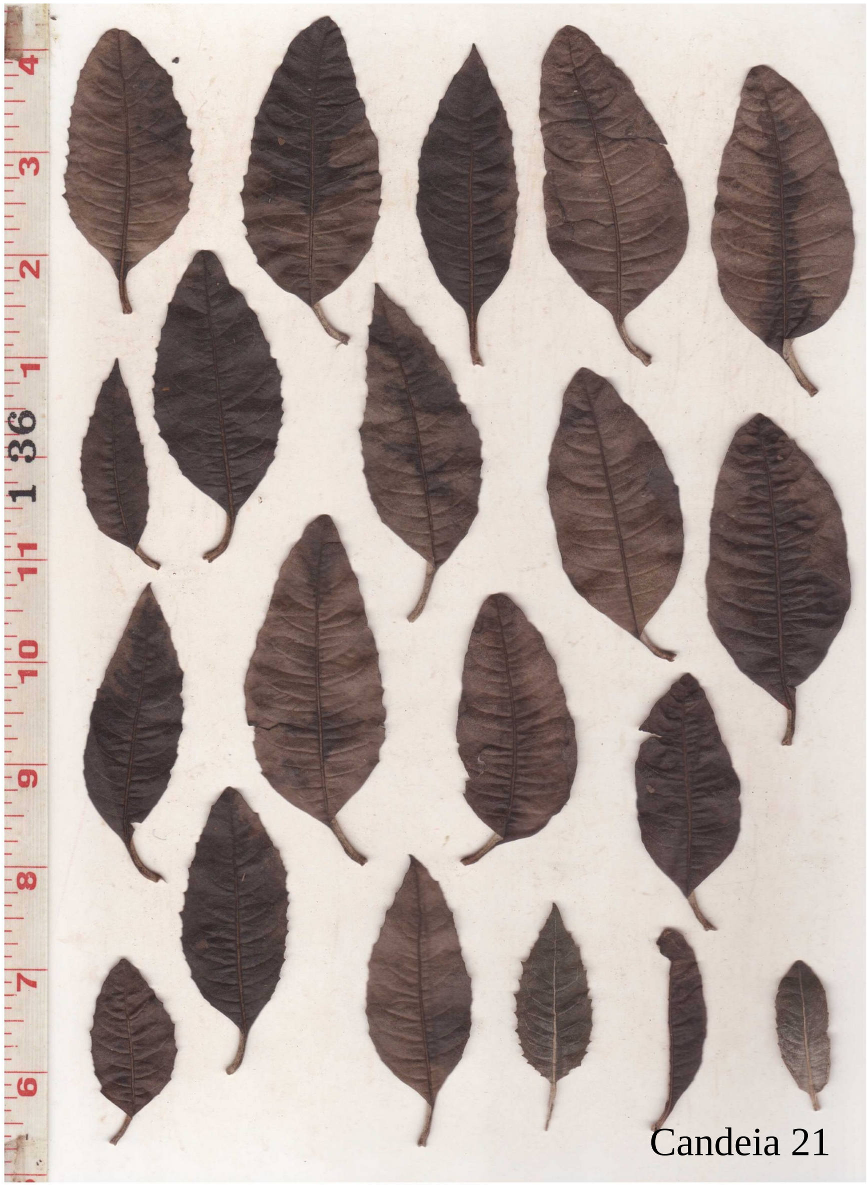

Candeia 21

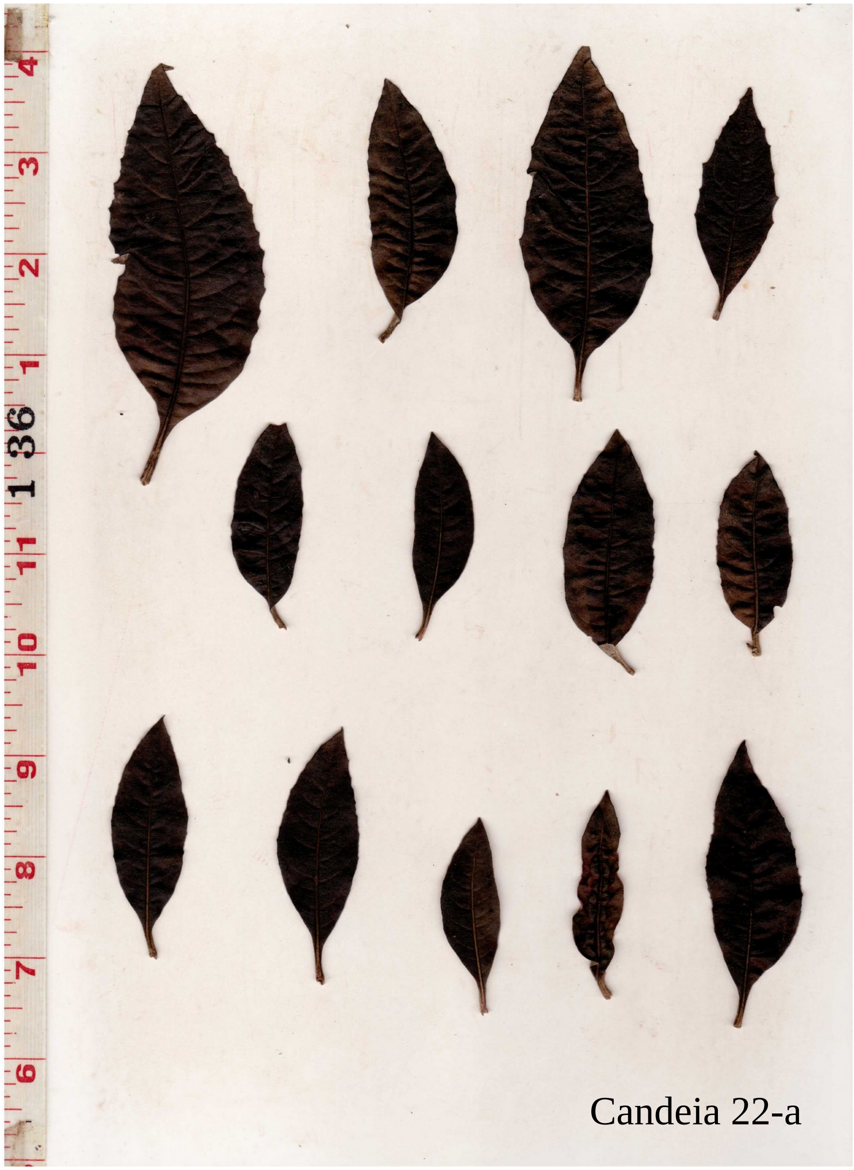

Candeia 22-a

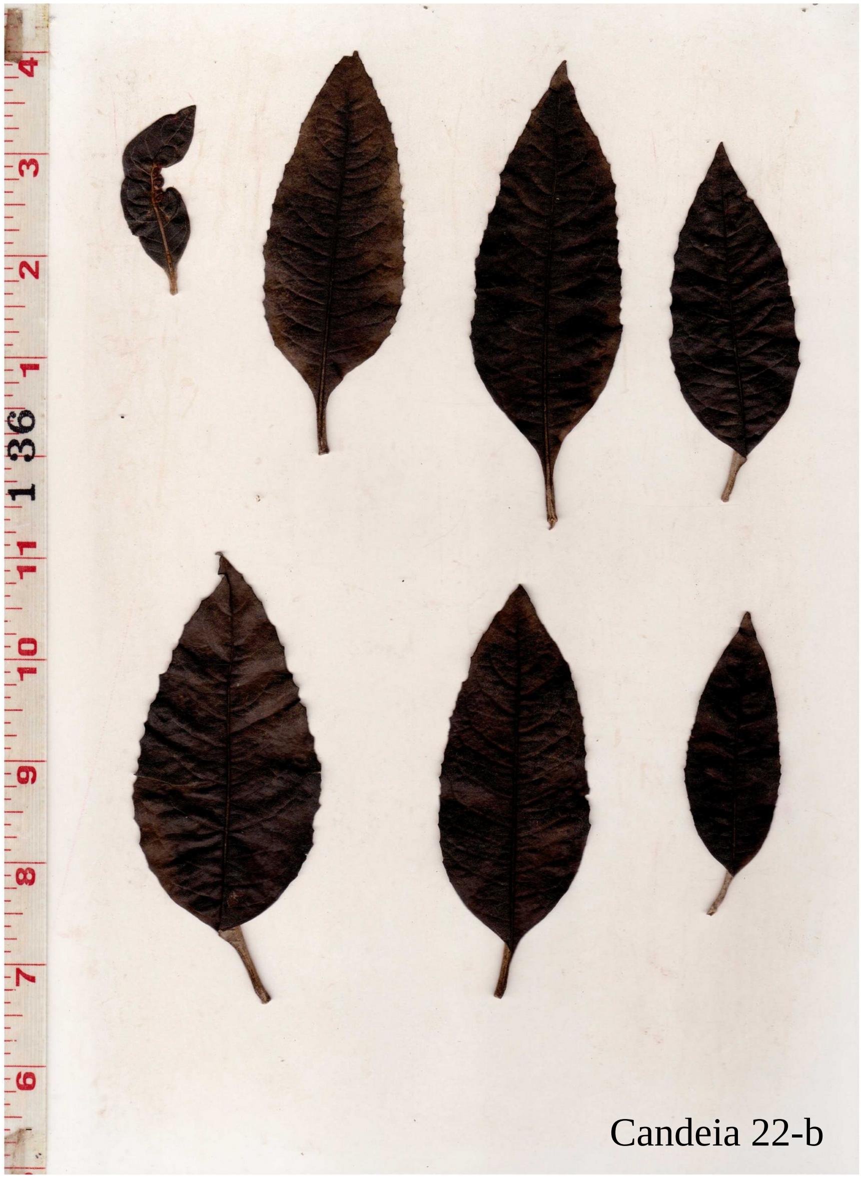

Candeia 22-b

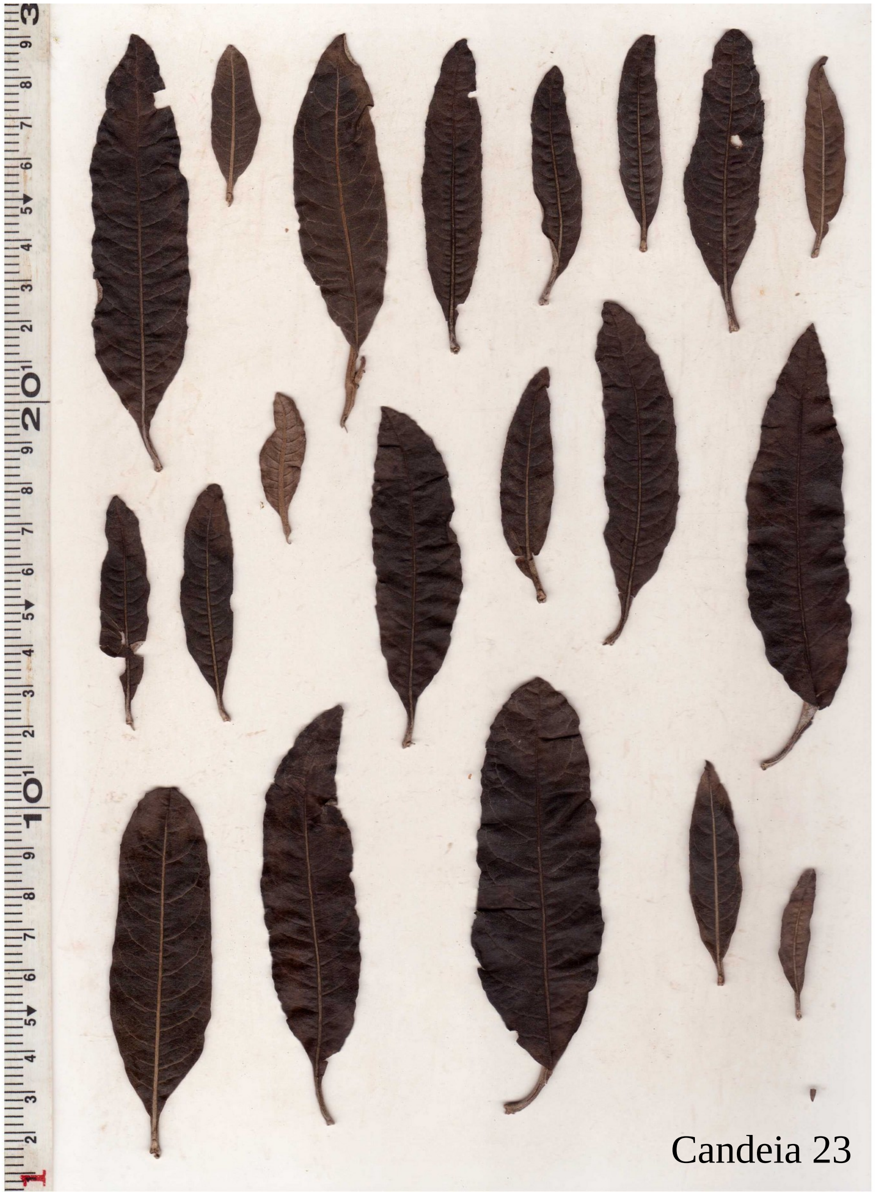

Candeia 23

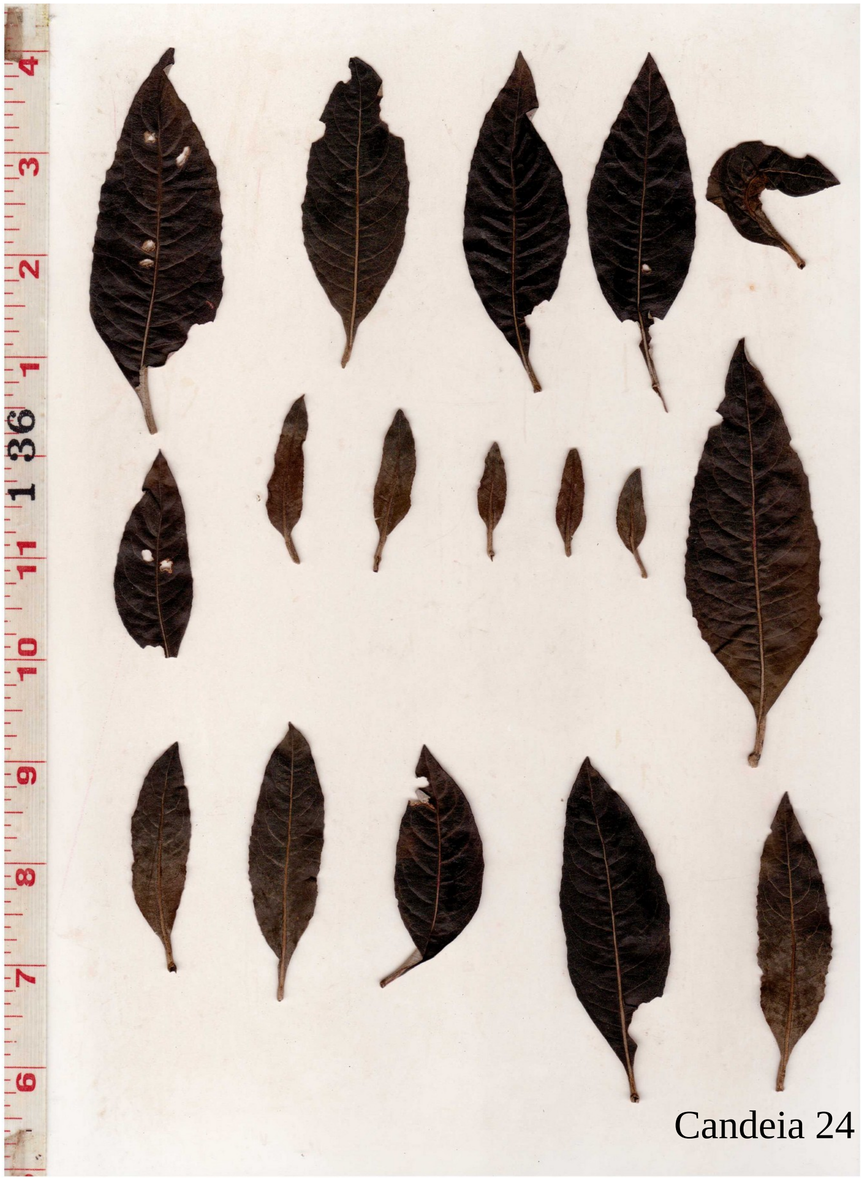

Candeia 24

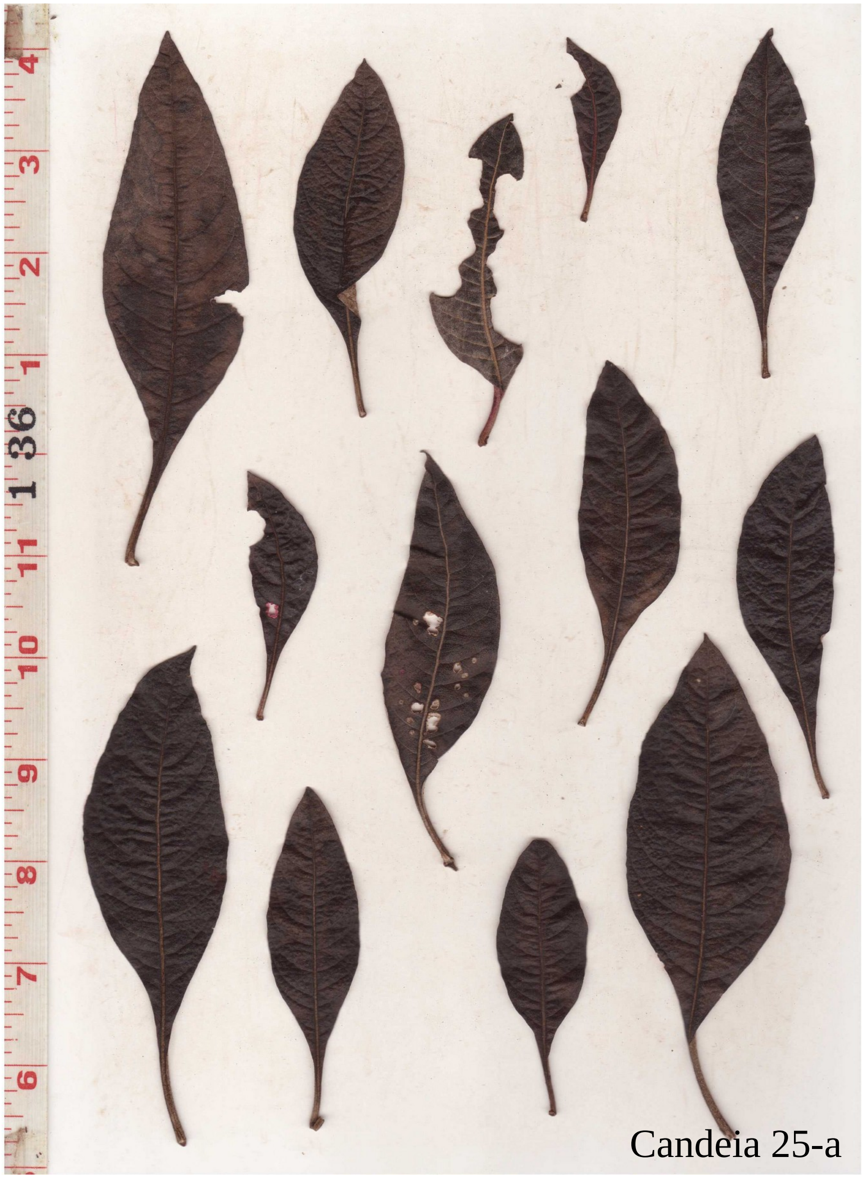

Candeia 25-a

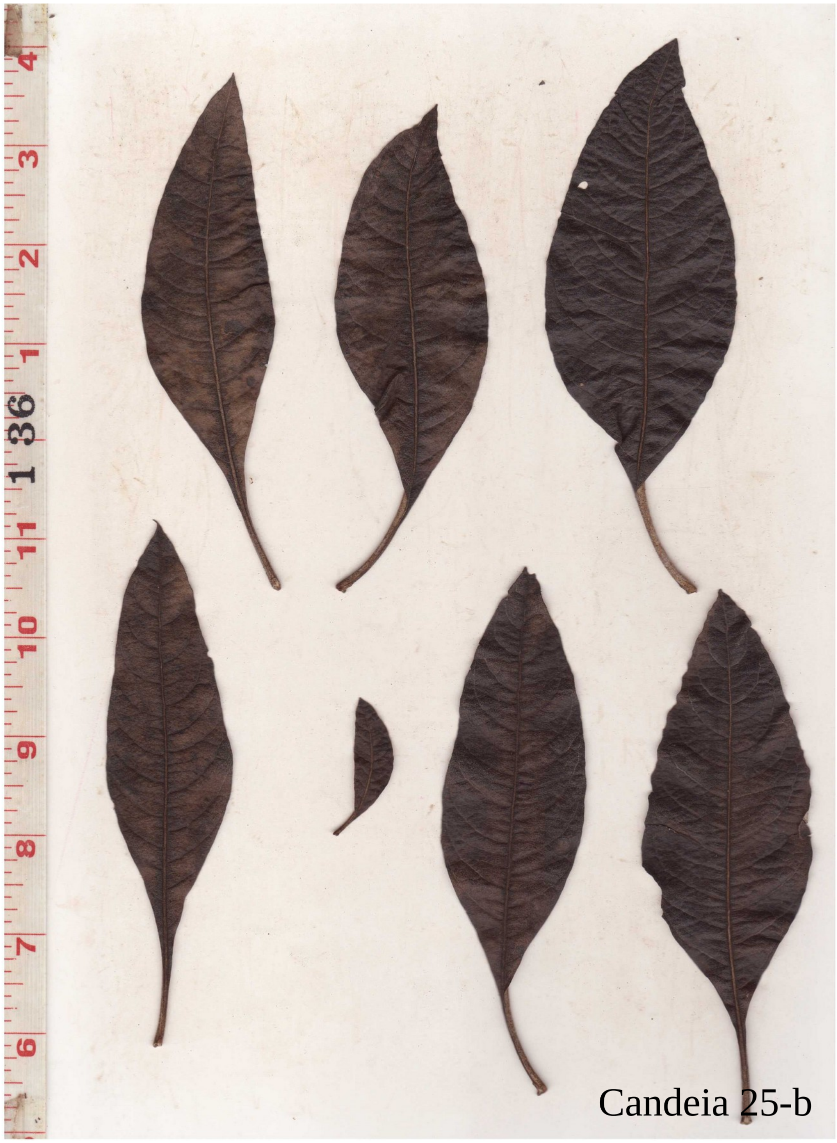

Candeia 25-b

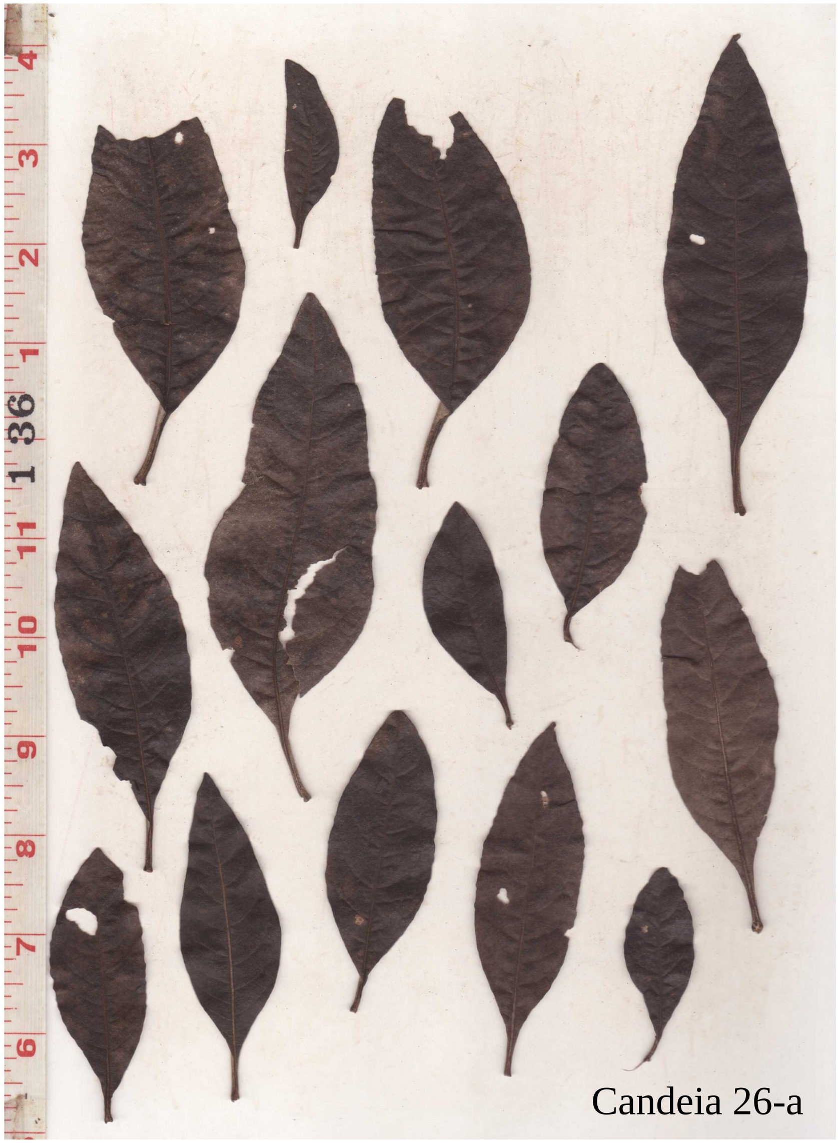

Candeia 26-a

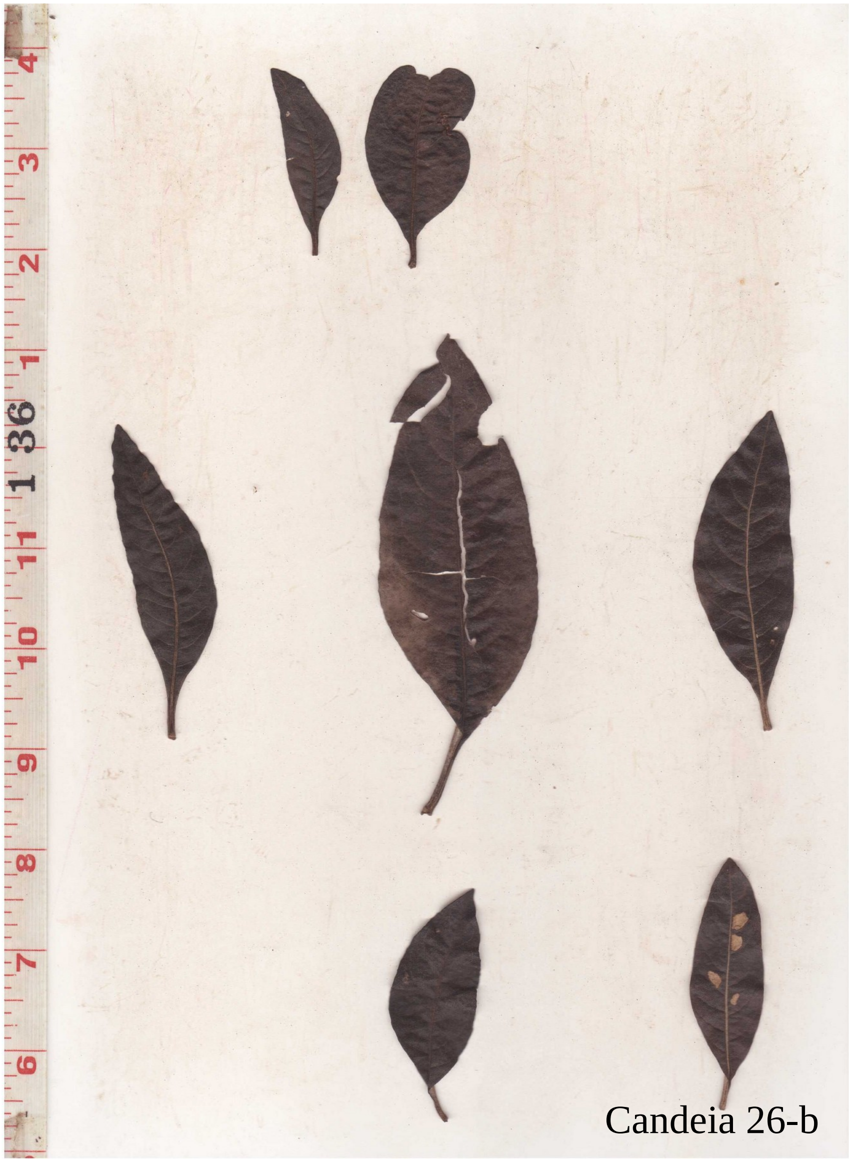
Candeia 26-b

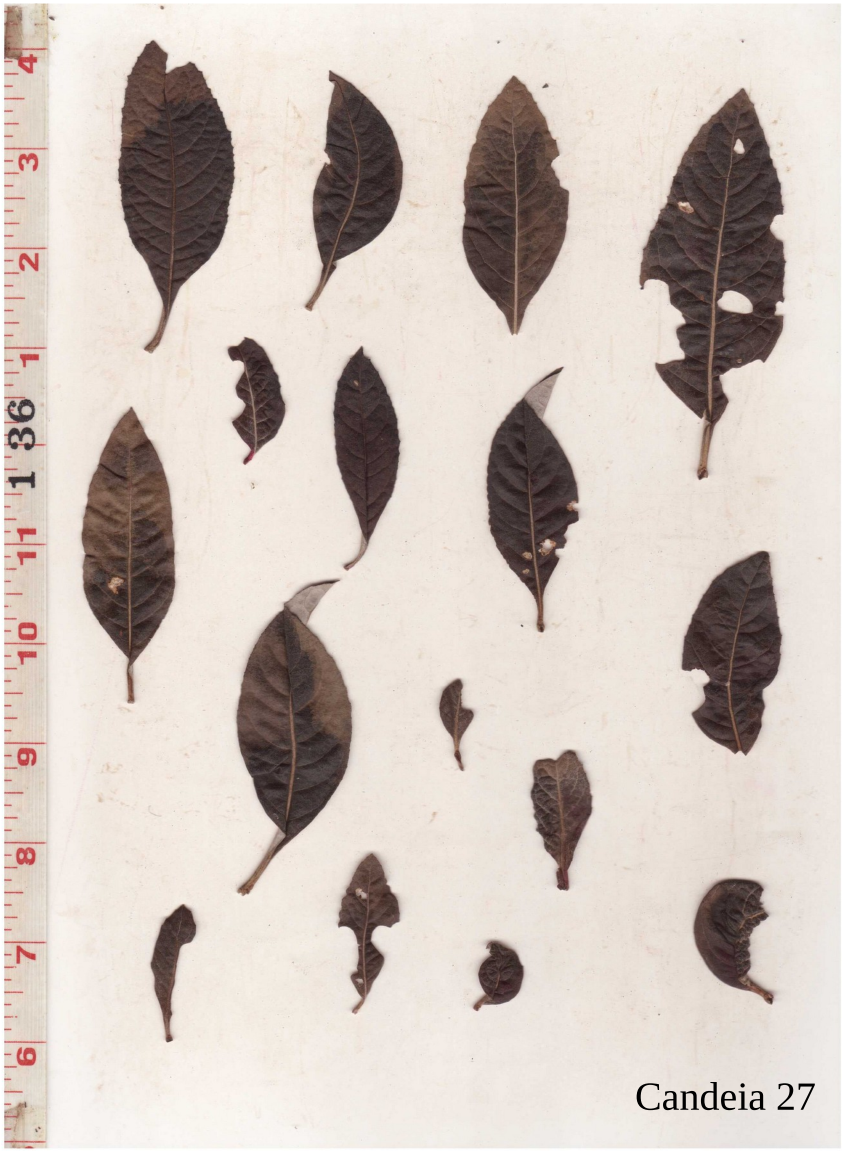
Candeia 27

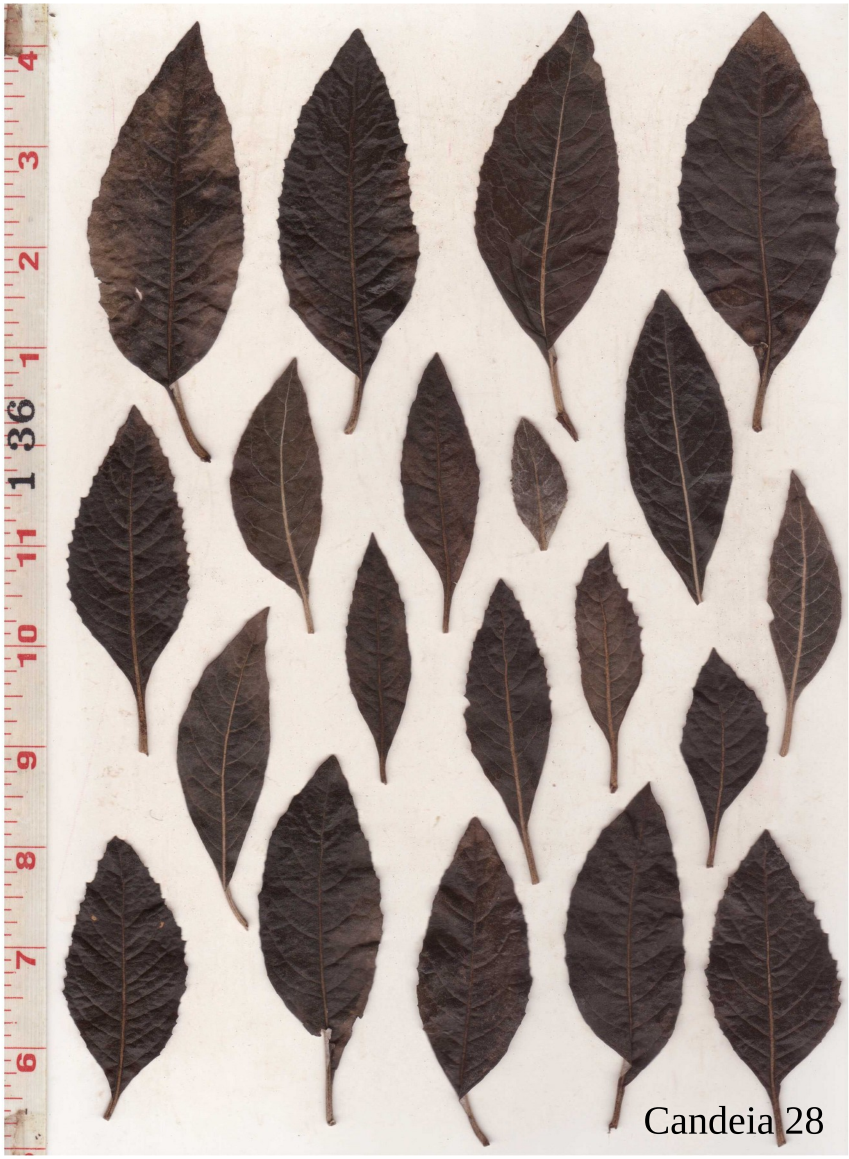
Candeia 28

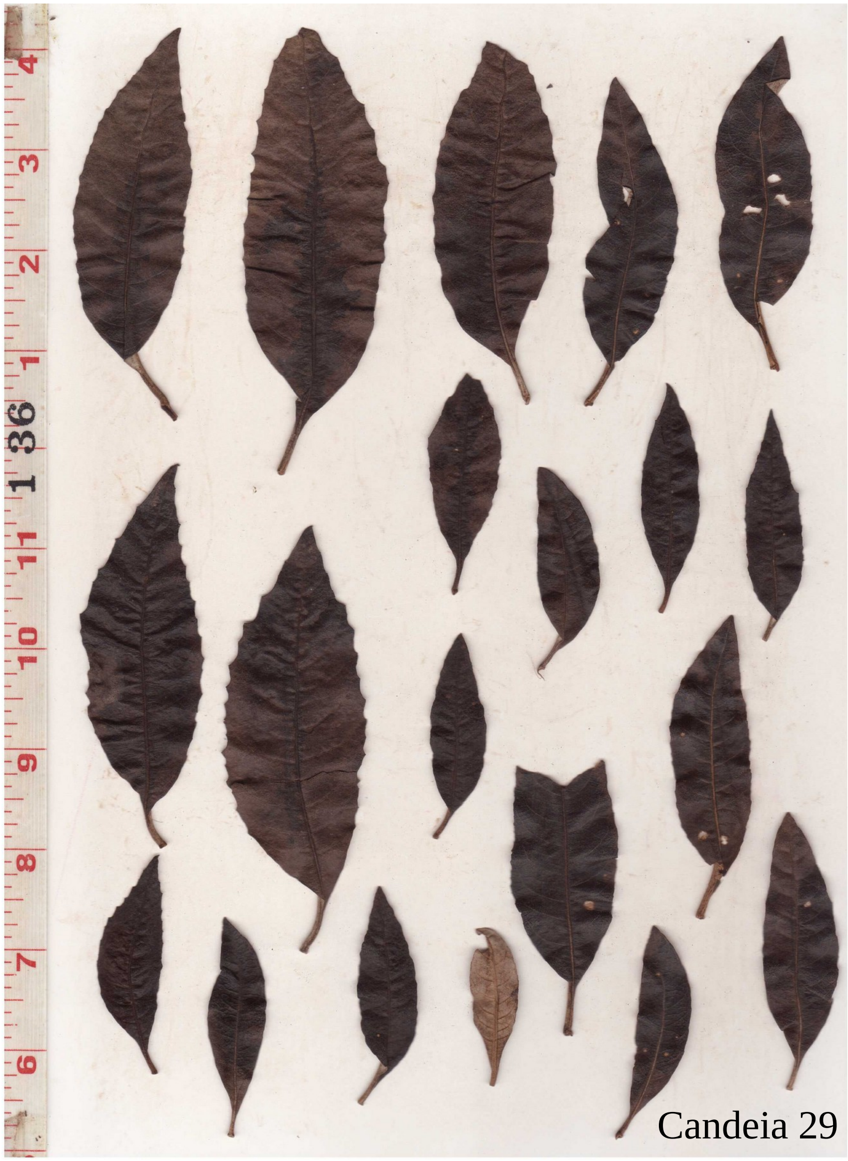
Candeia 29

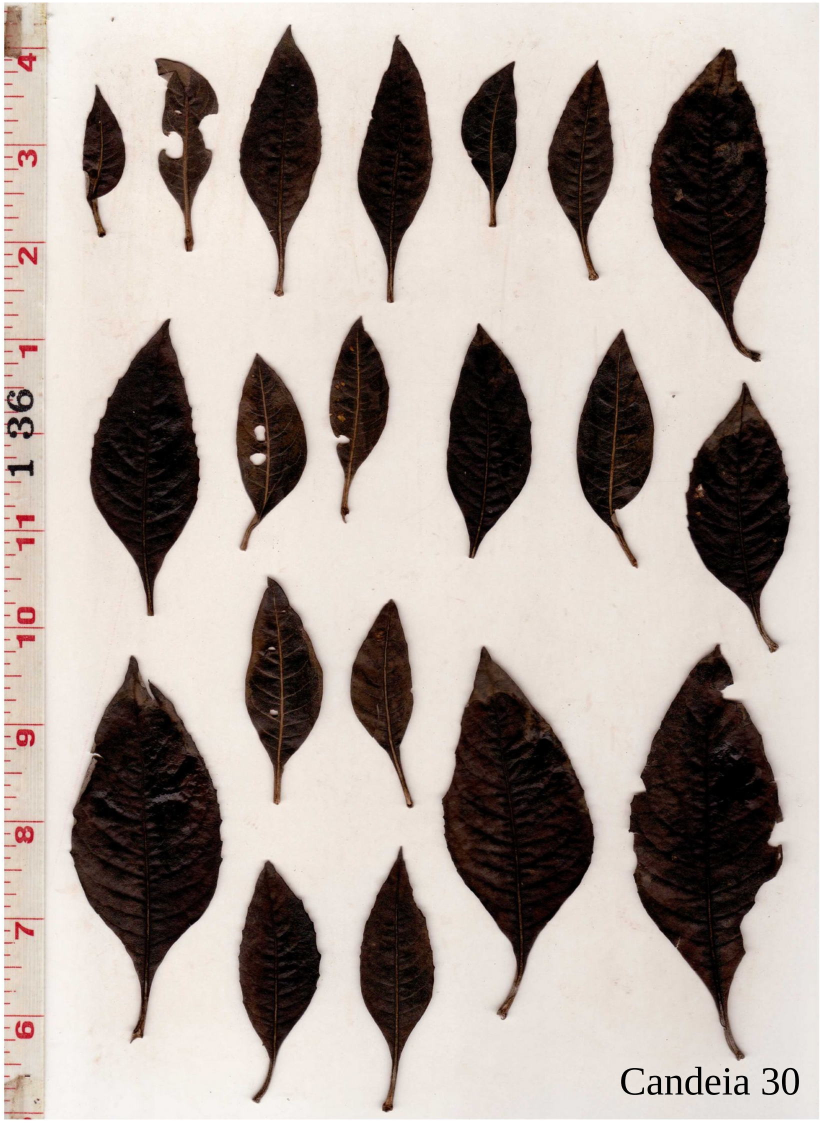

Candeia 30

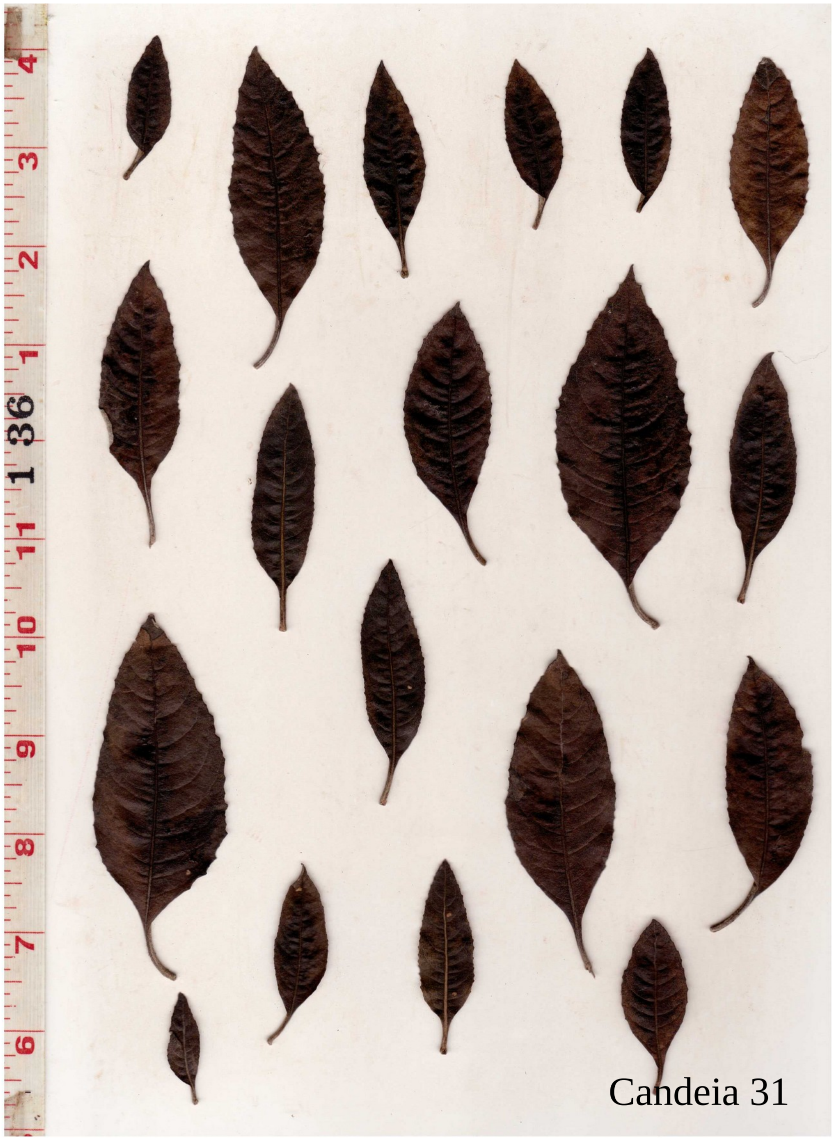

Candeia 31

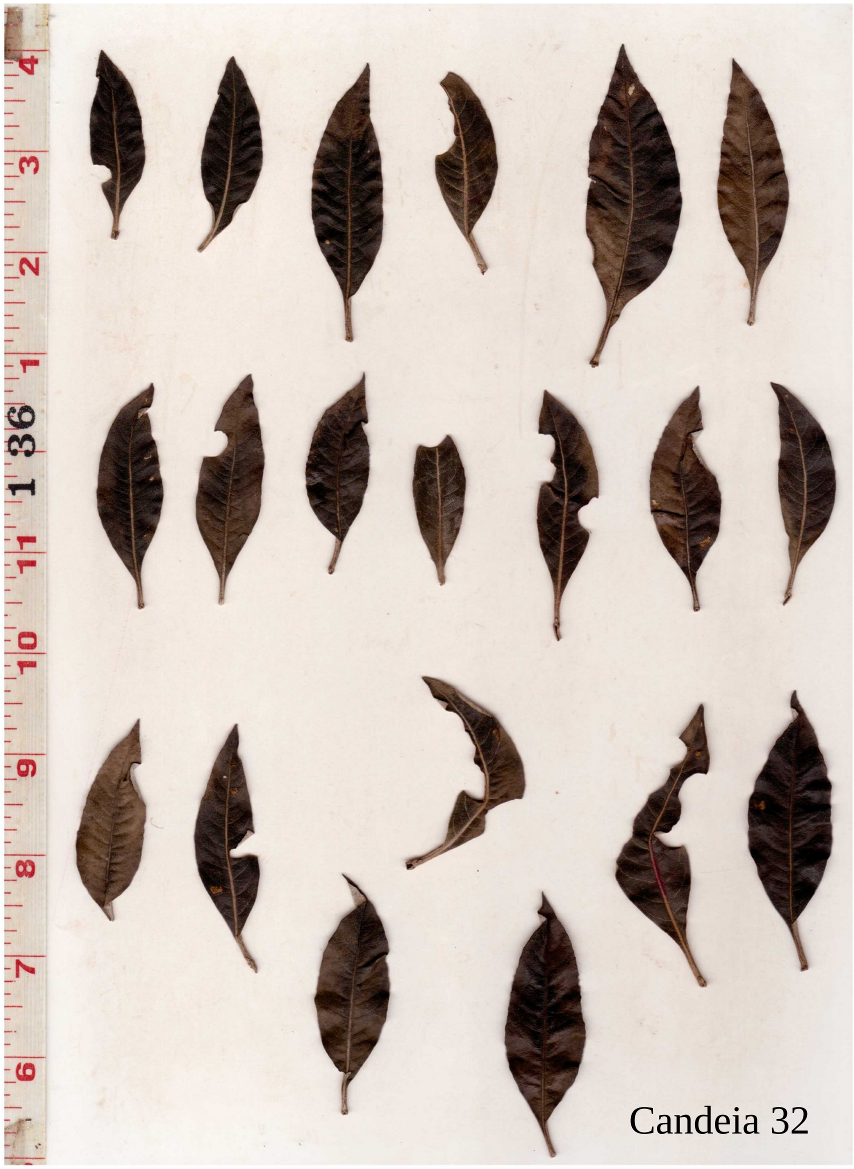
Candeia 32

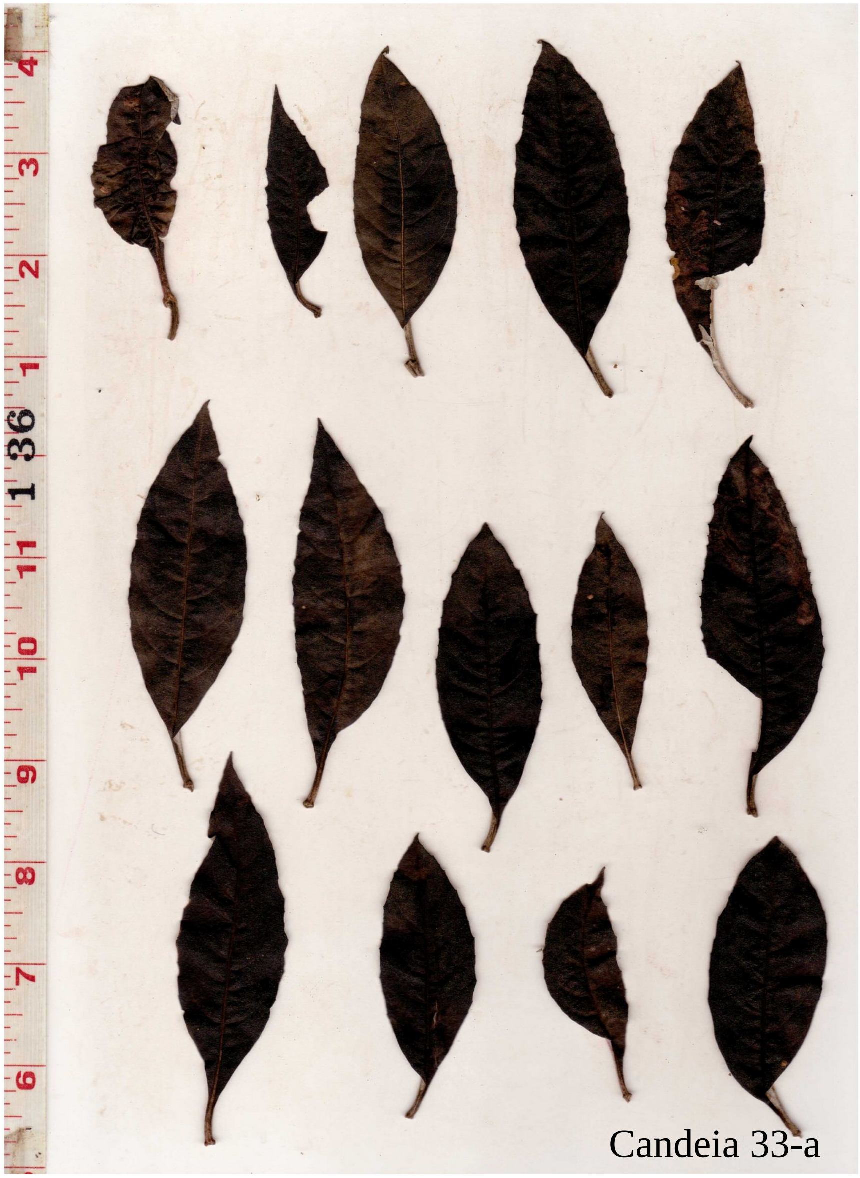
Candeia 33-a

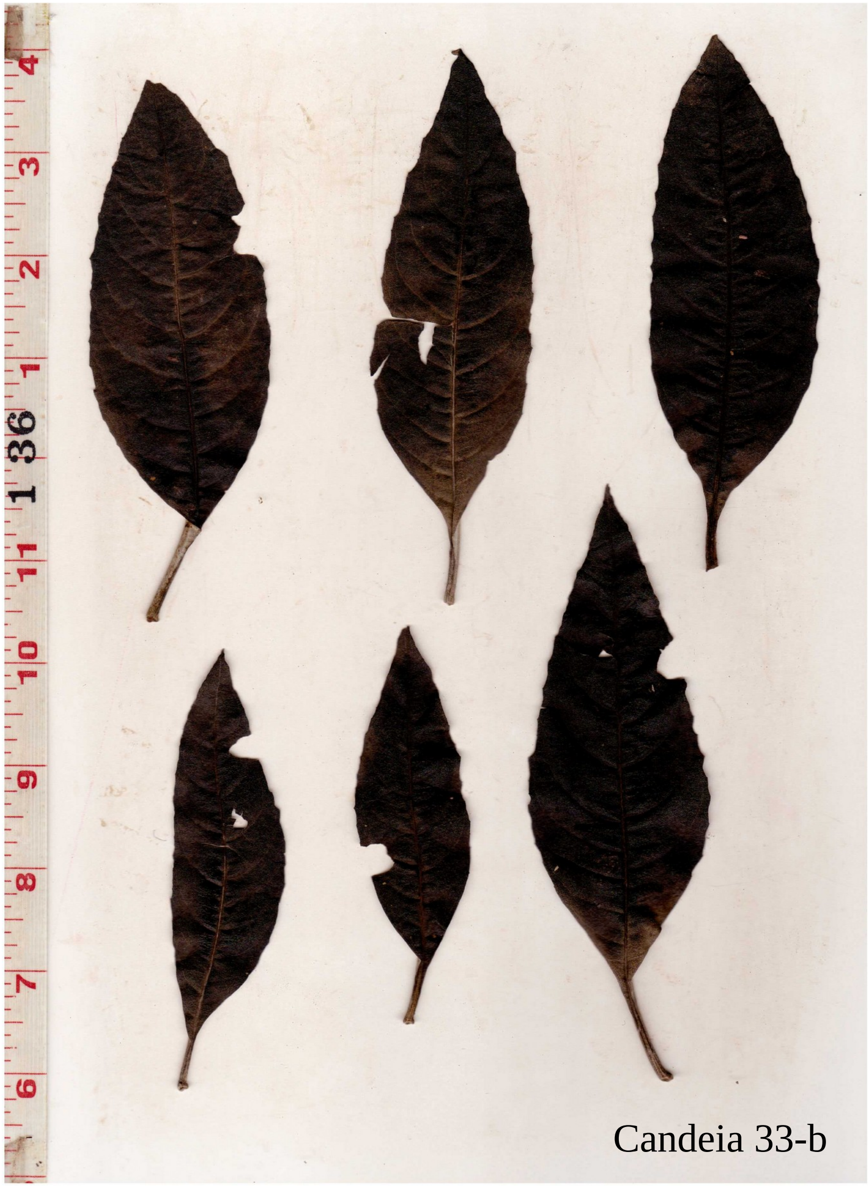

Candeia 33-b

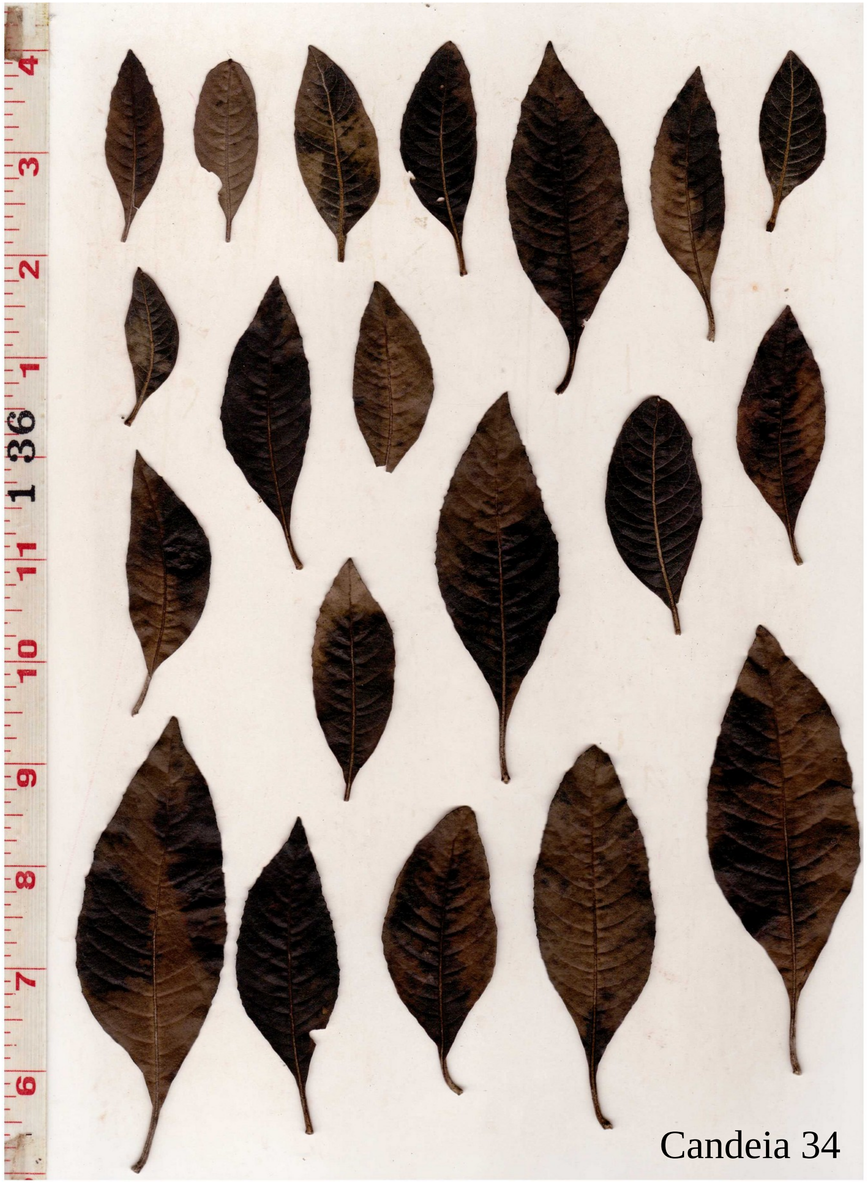

Candeia 34

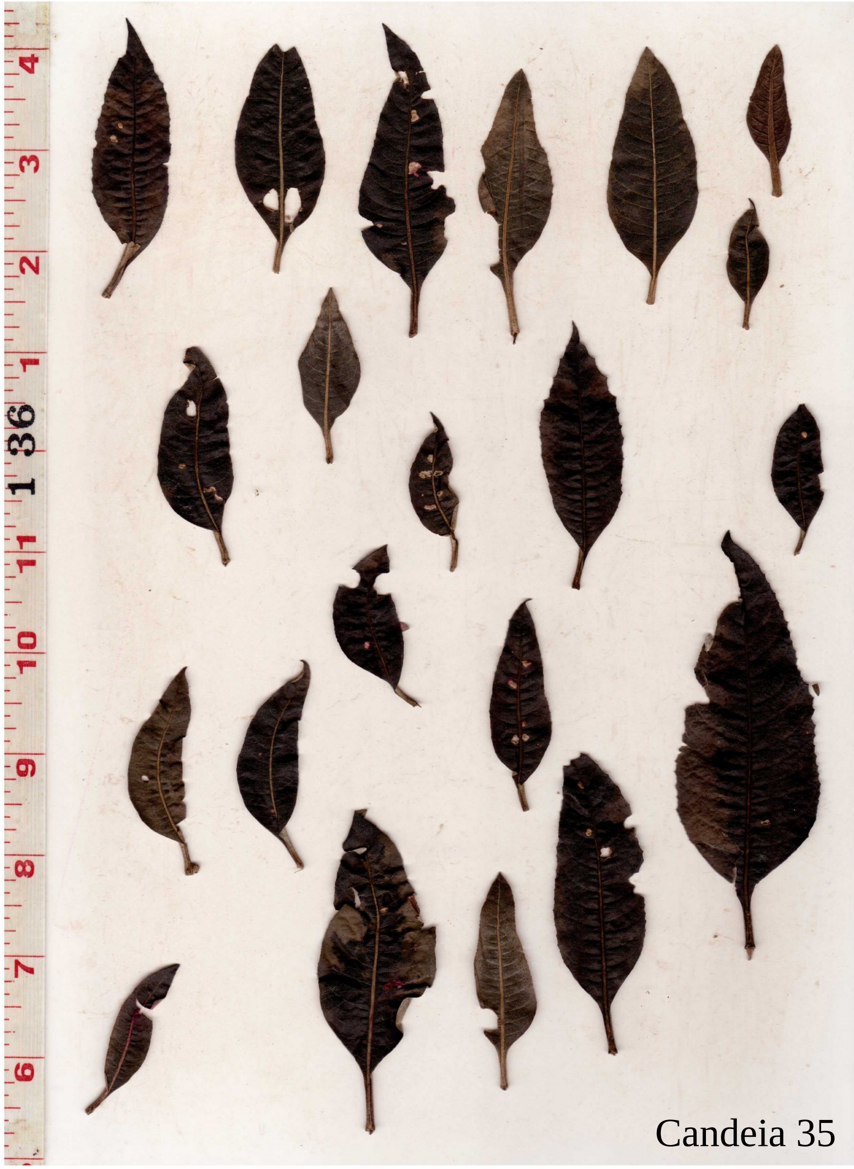
Candeia 35

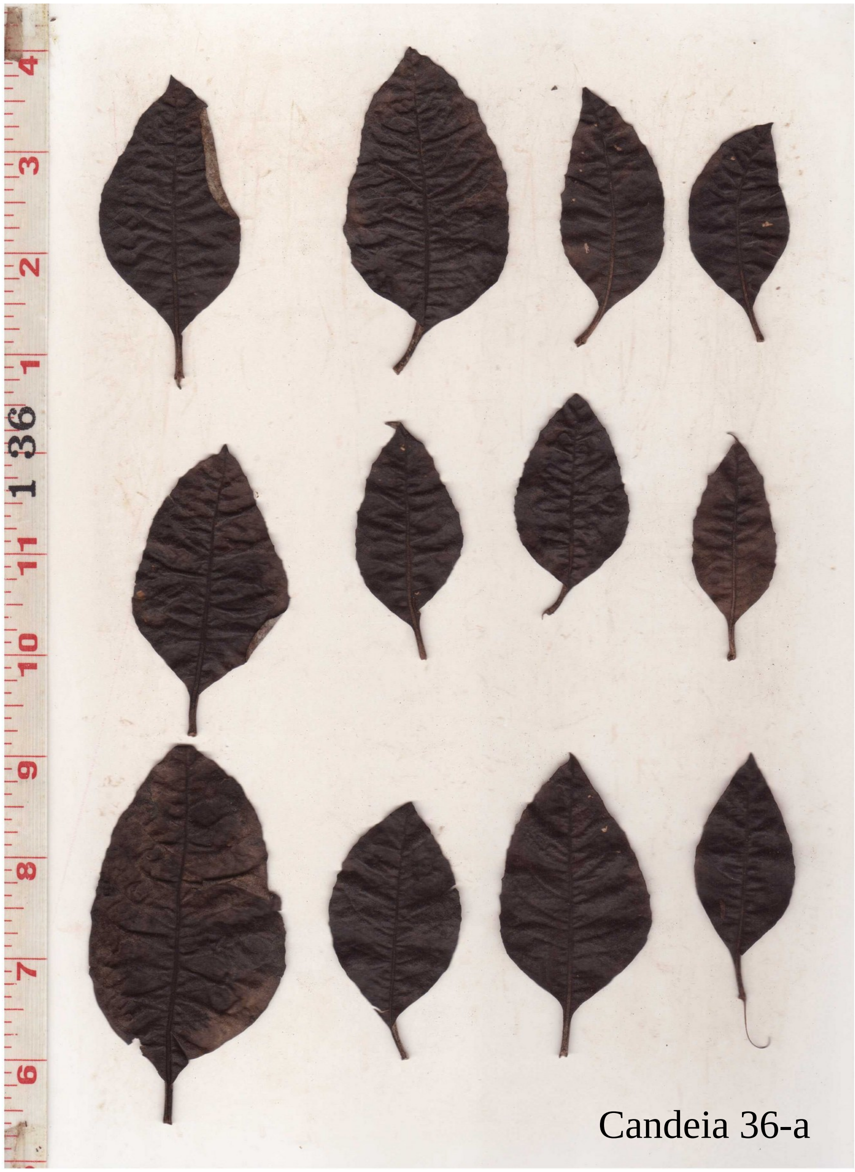
Candeia 36-a

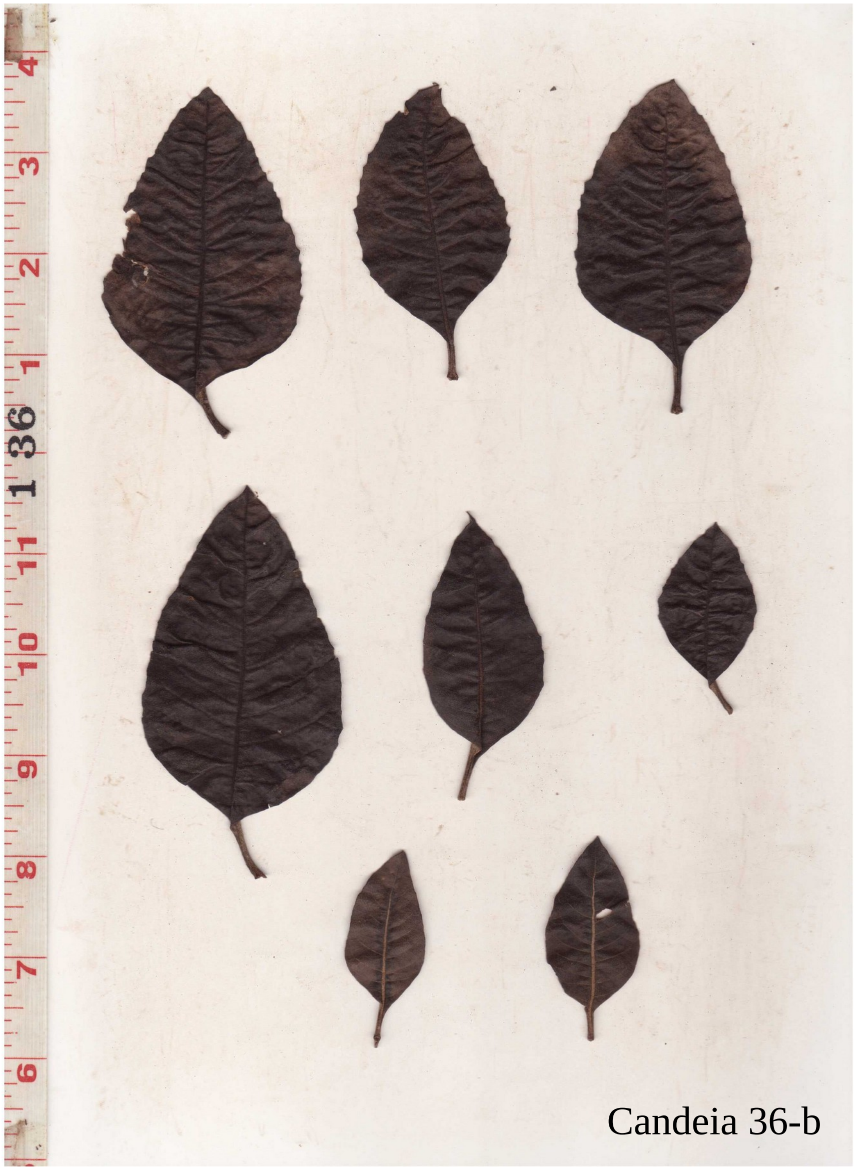

Candeia 36-b

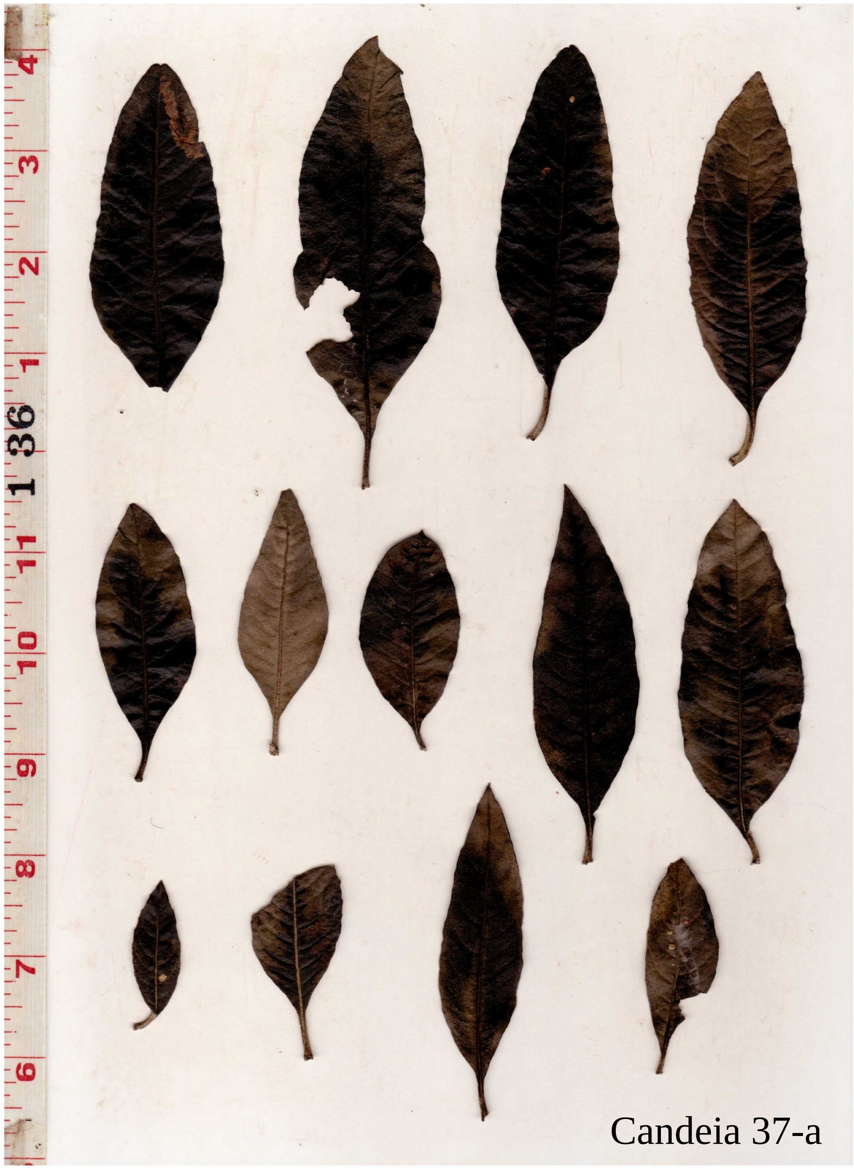

Candeia 37-a

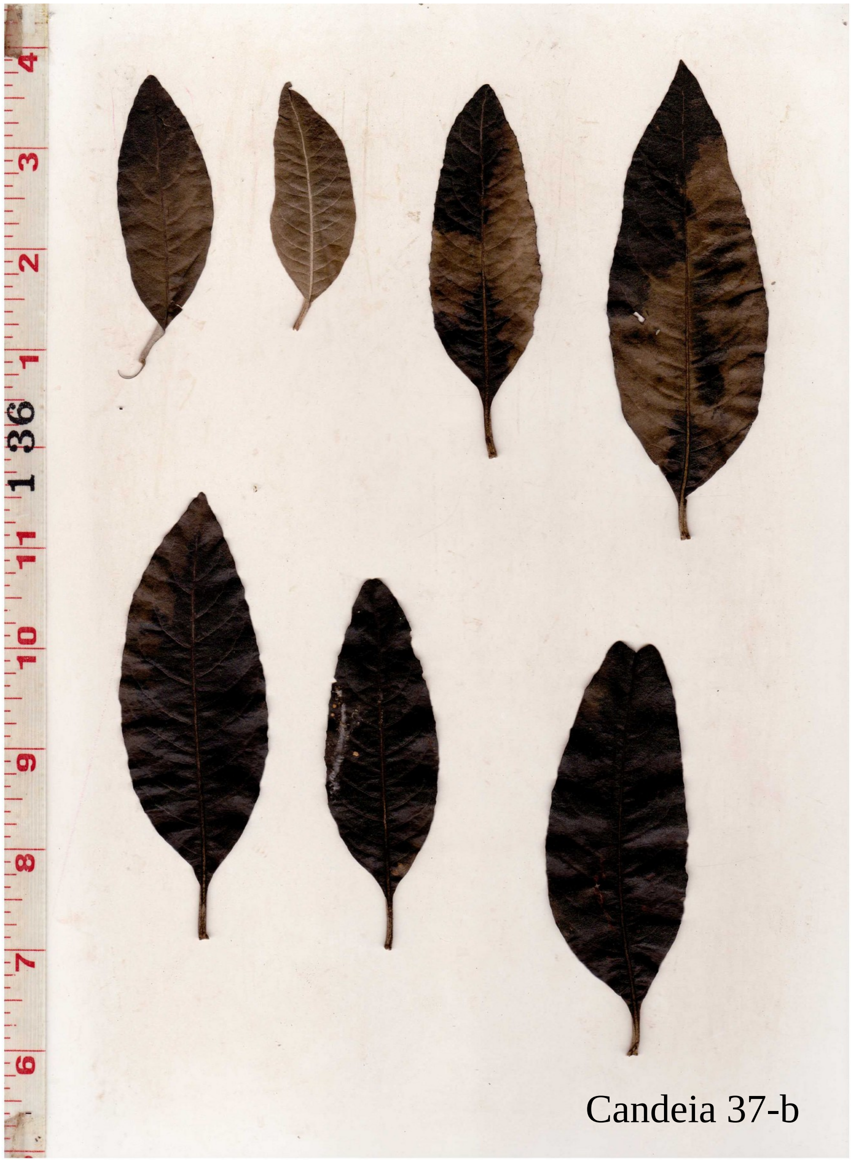

Candeia 37-b

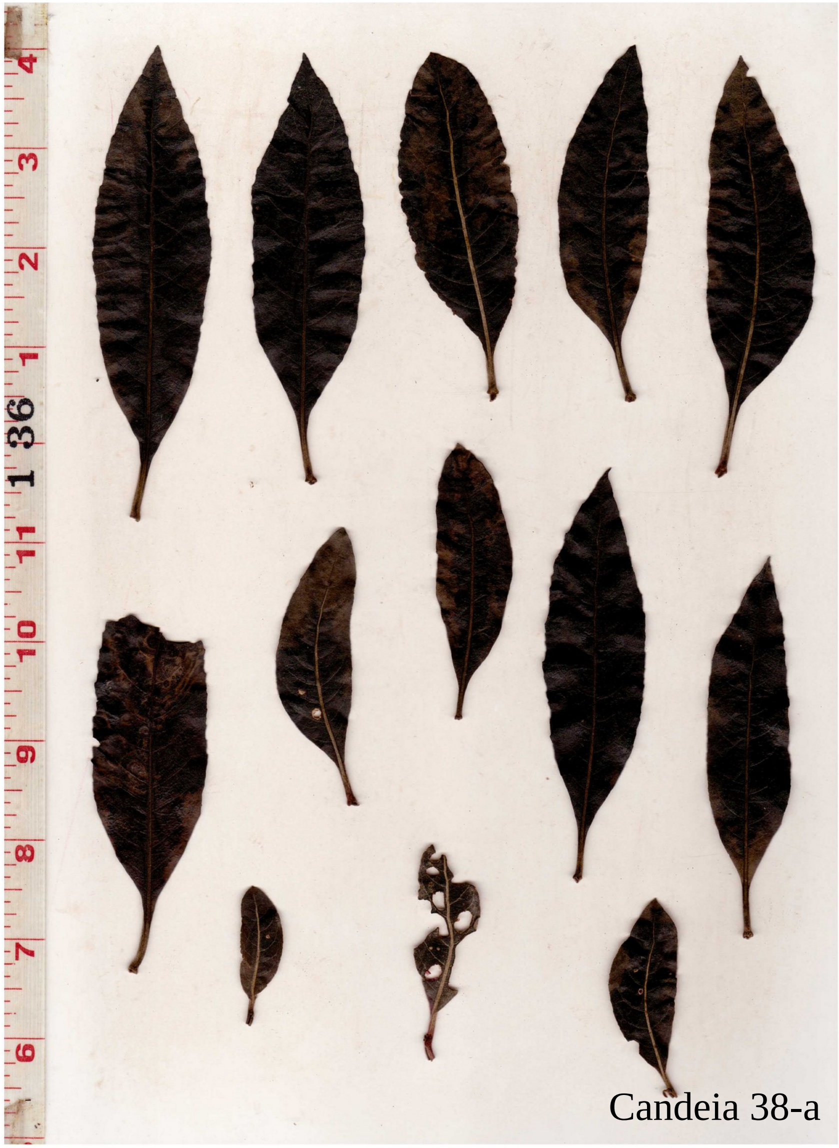

Candeia 38-a

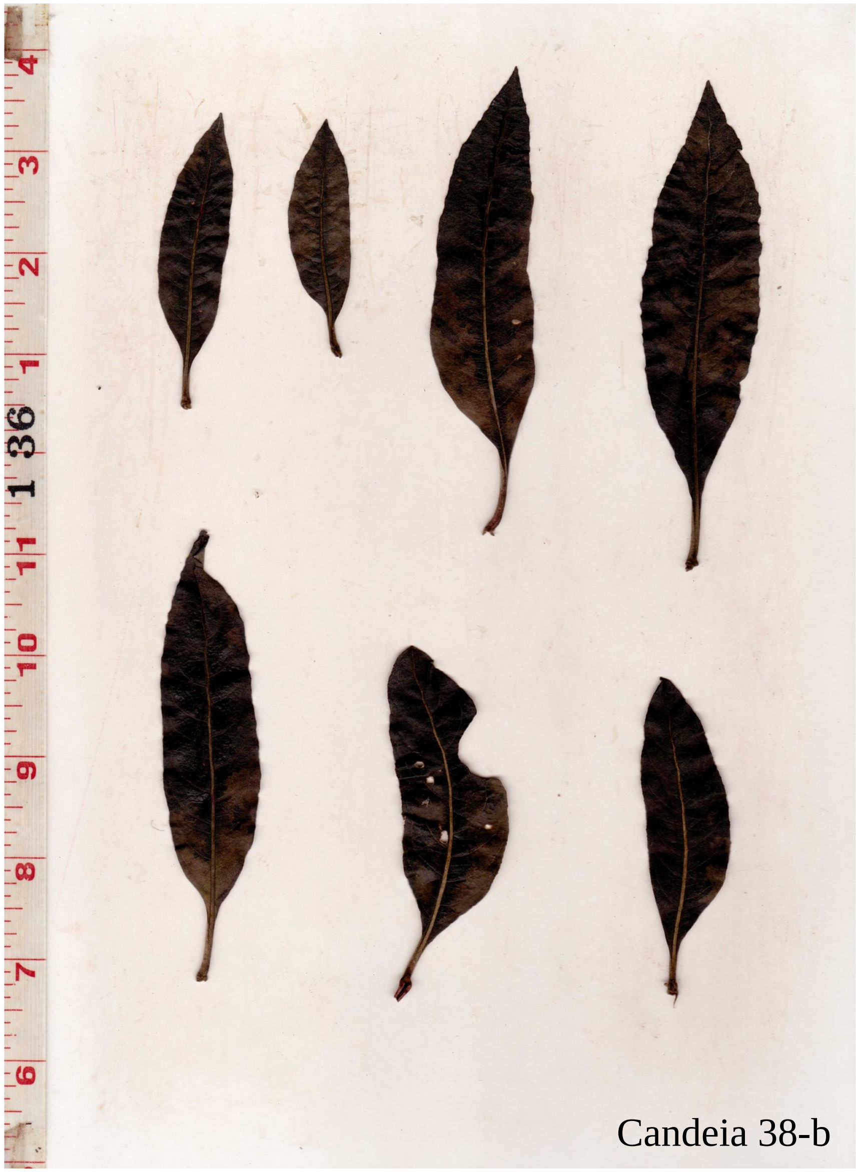
Candeia 38-b

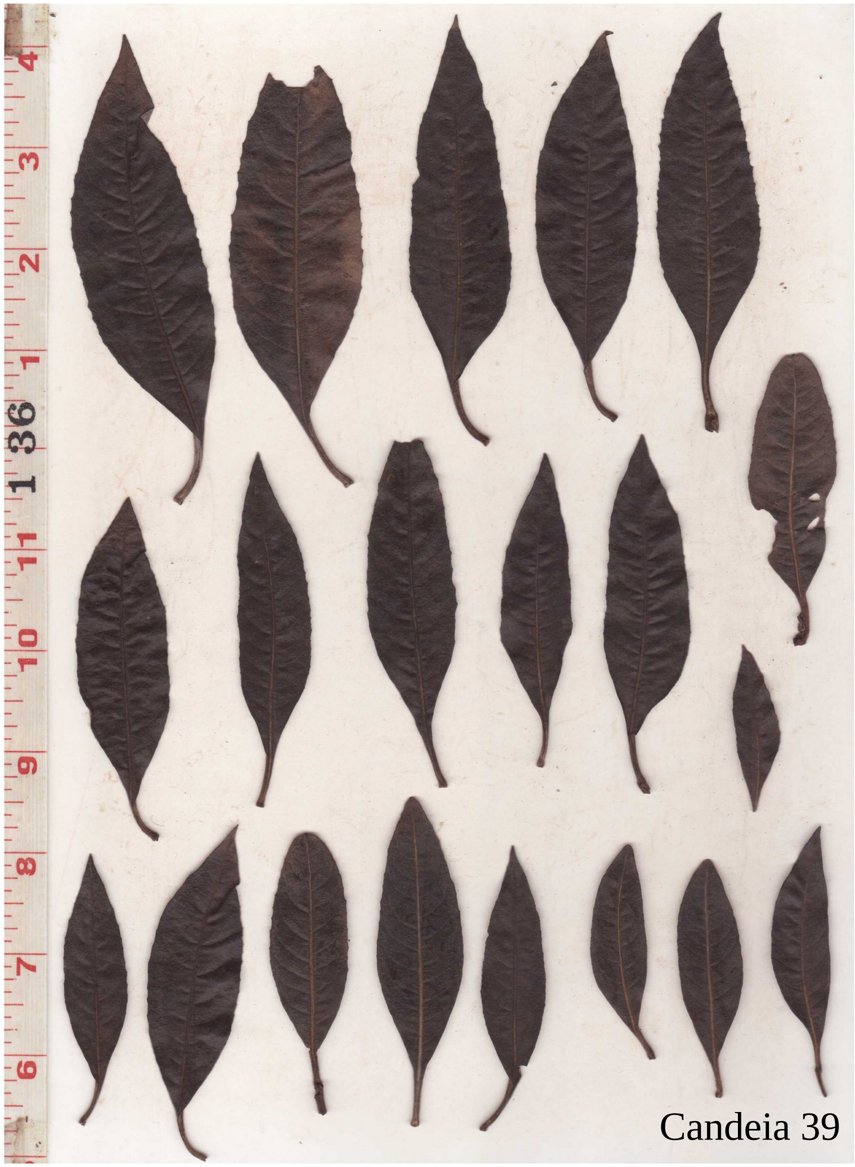
Candeia 39

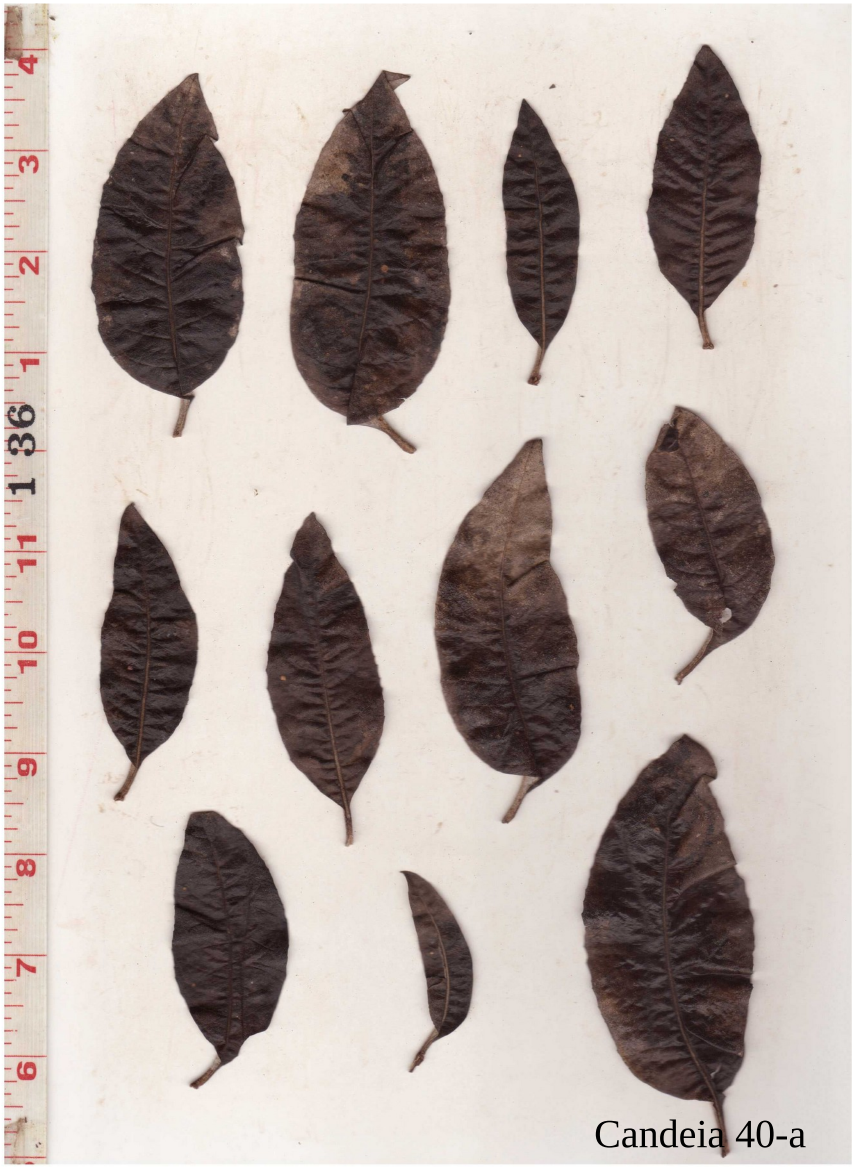

Candeia 40-a

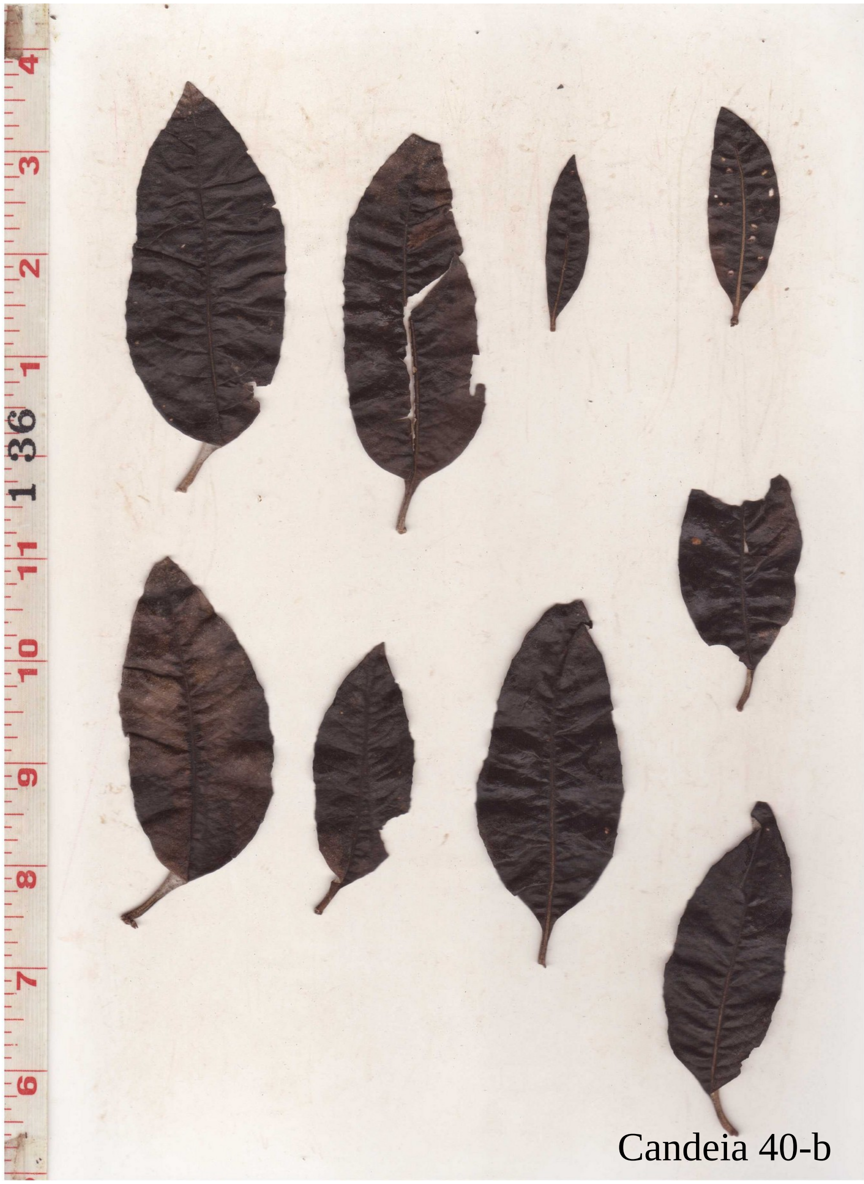

Candeia 40-b

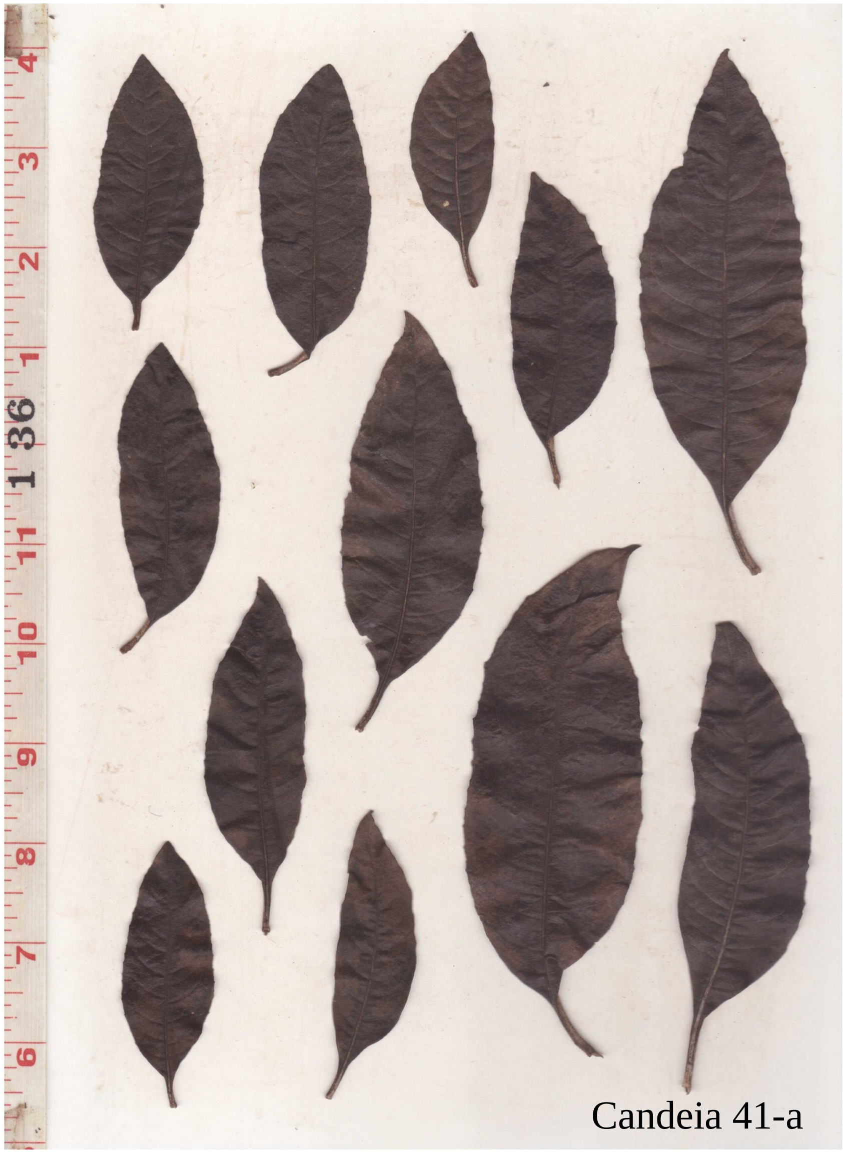

Candeia 41-a

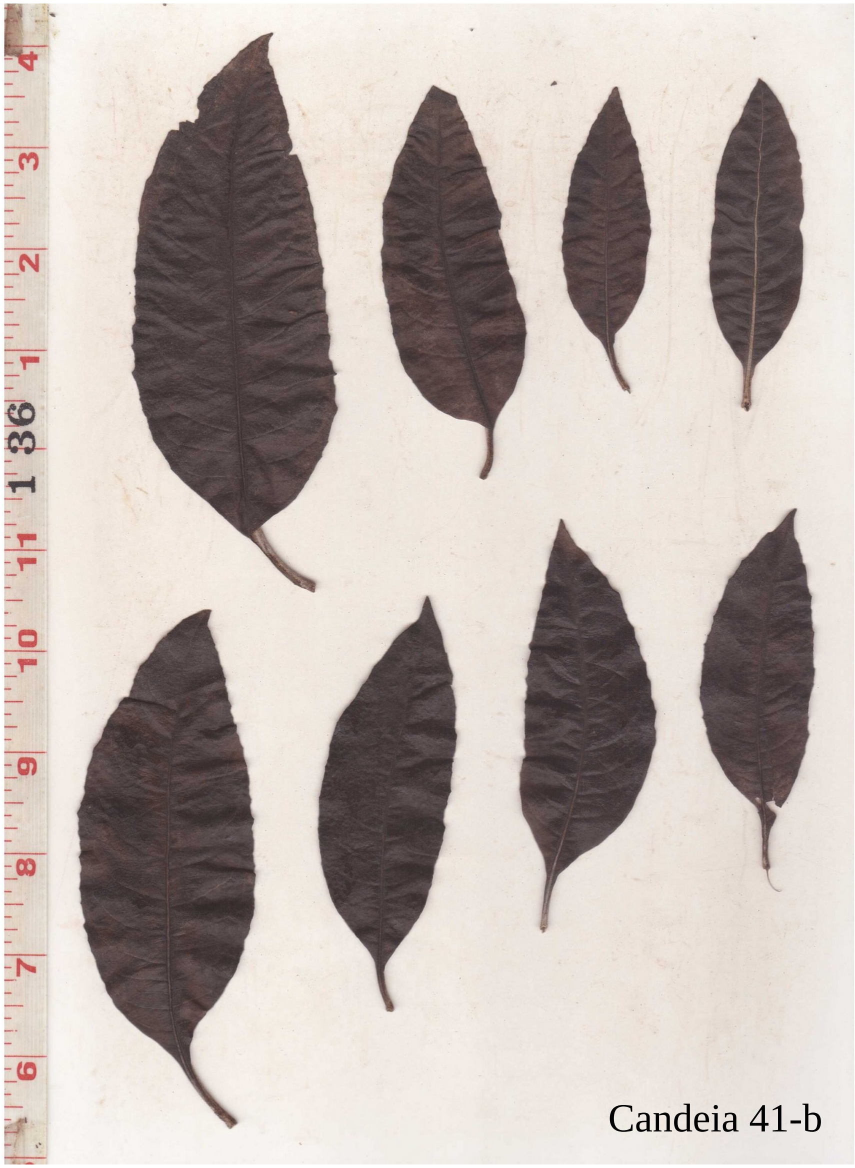

Candeia 41-b

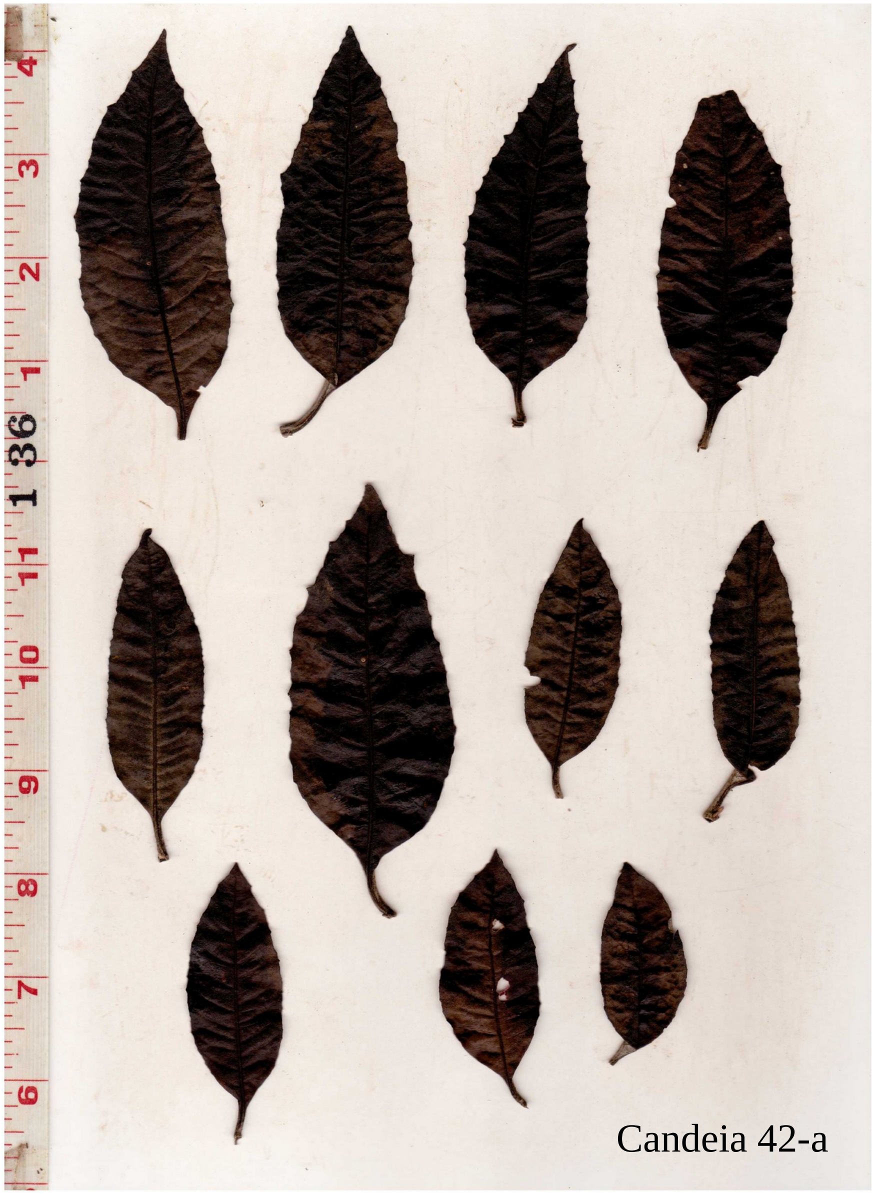

Candeia 42-a

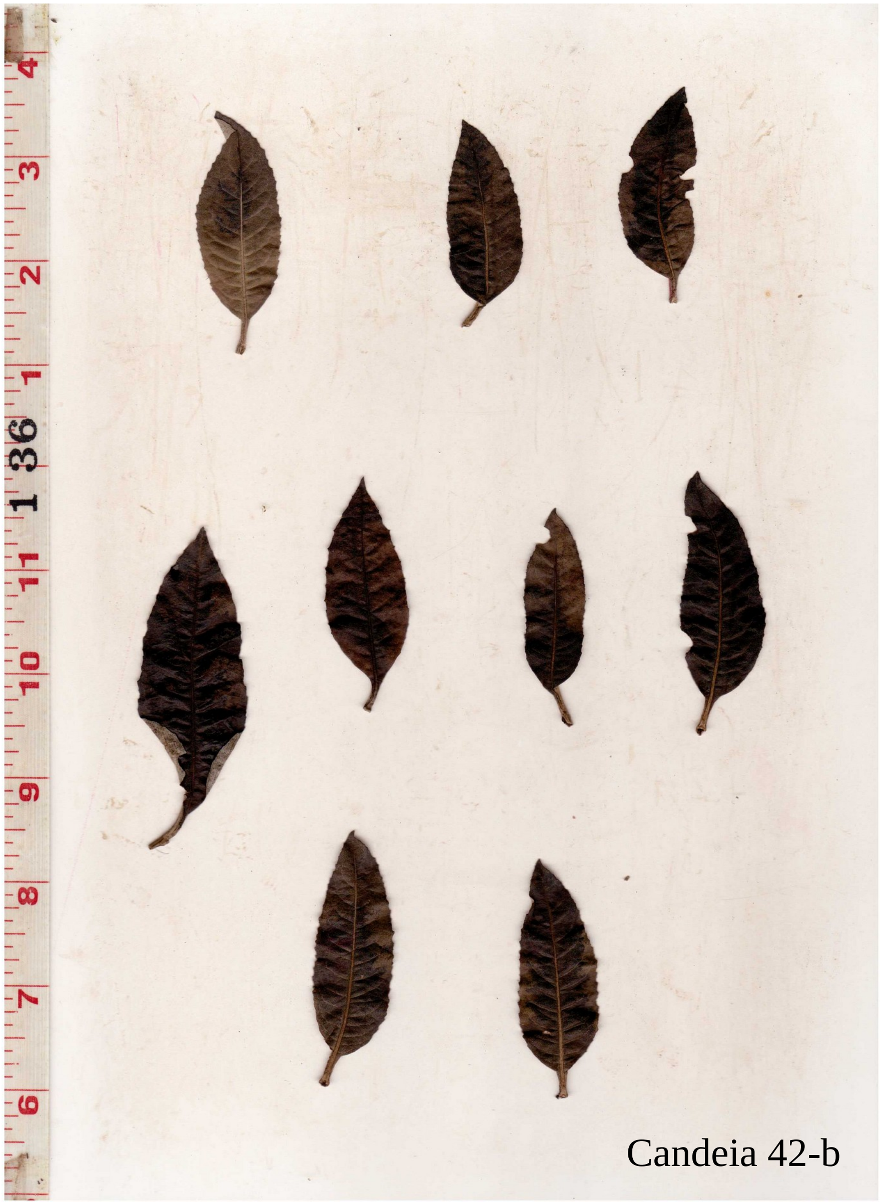

Candeia 42-b

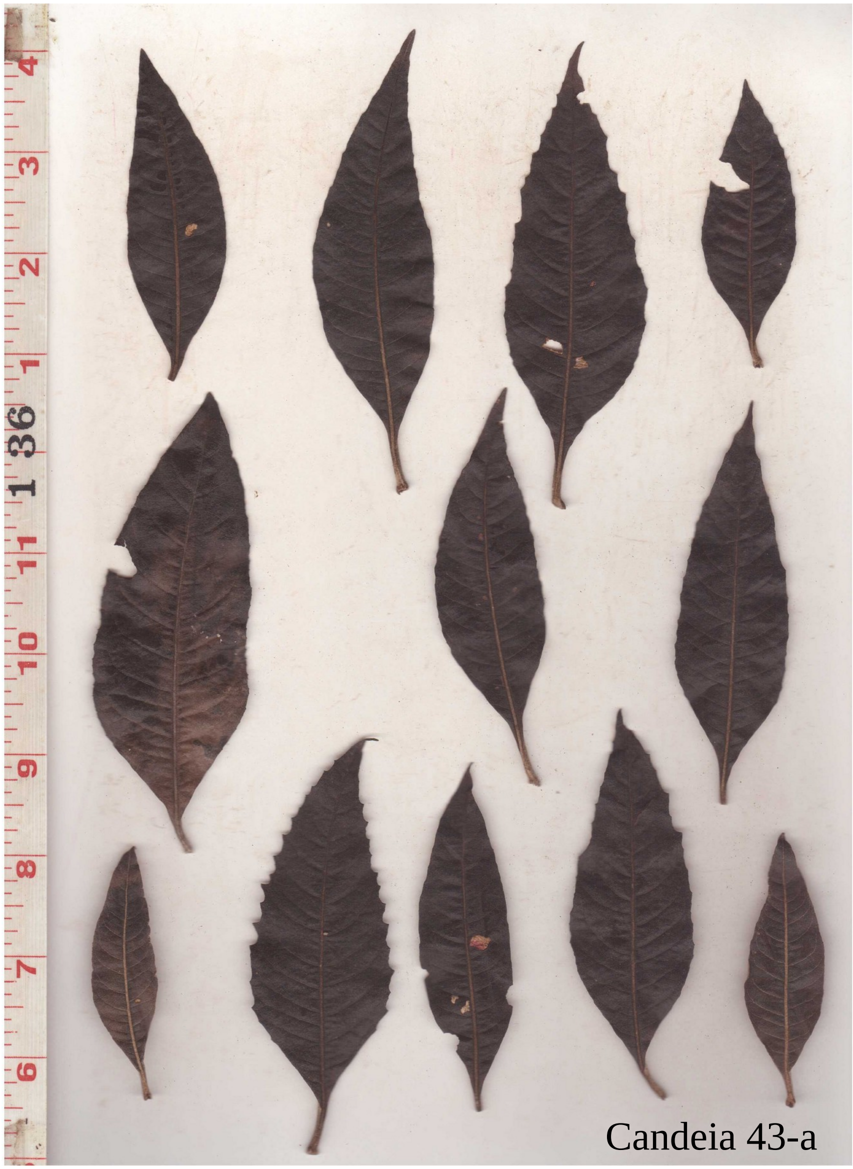

Candeia 43-a

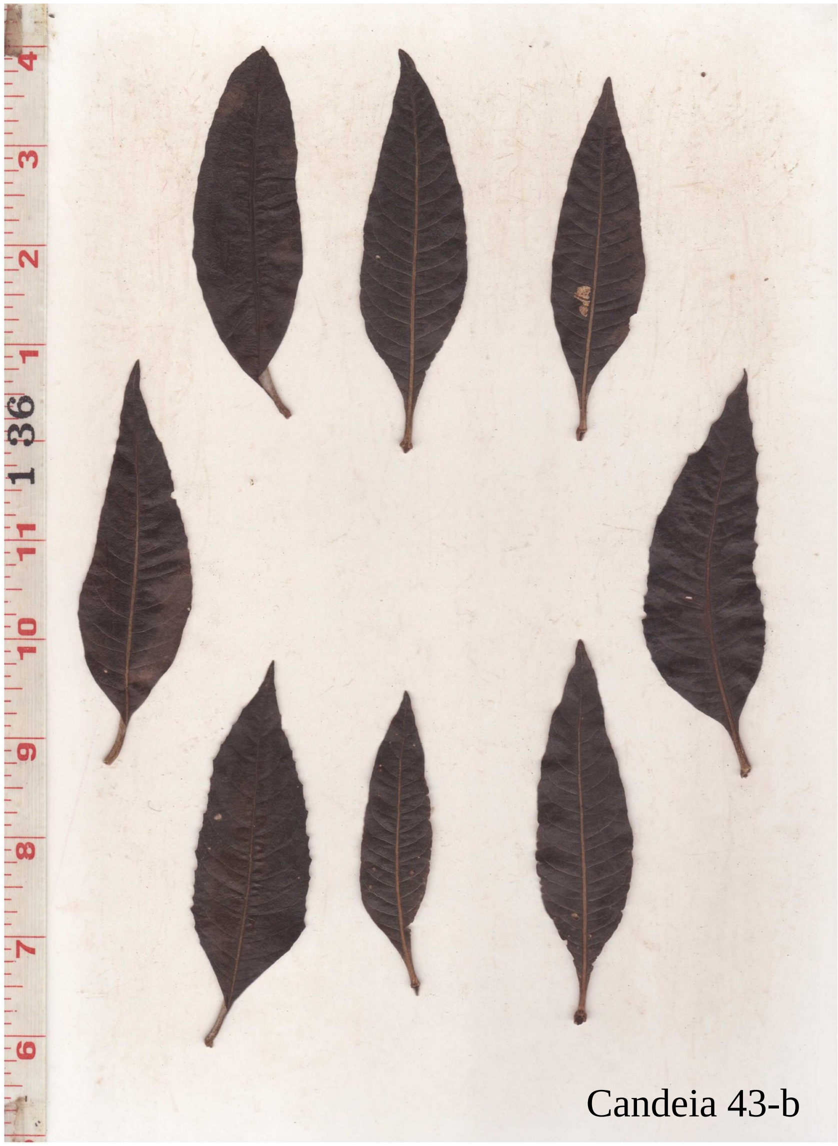

Candeia 43-b

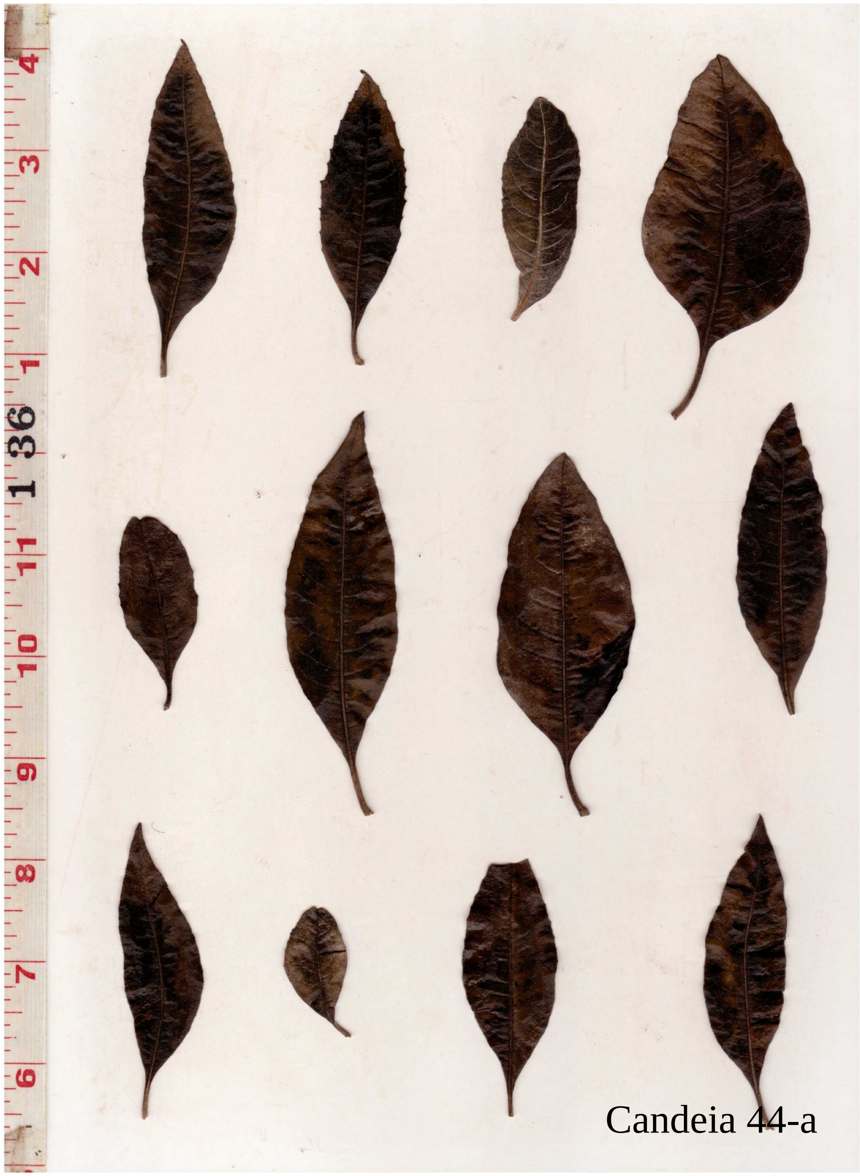

Candeia 44-a

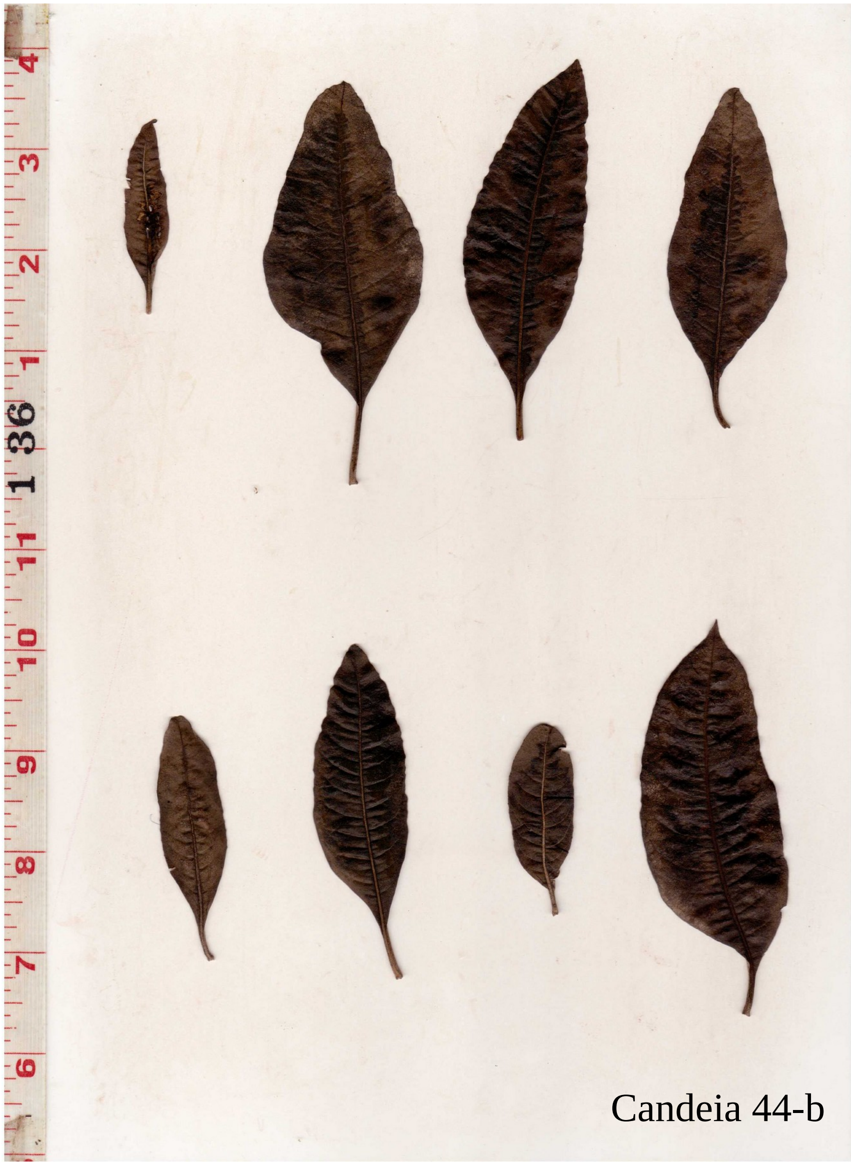

Candeia 44-b

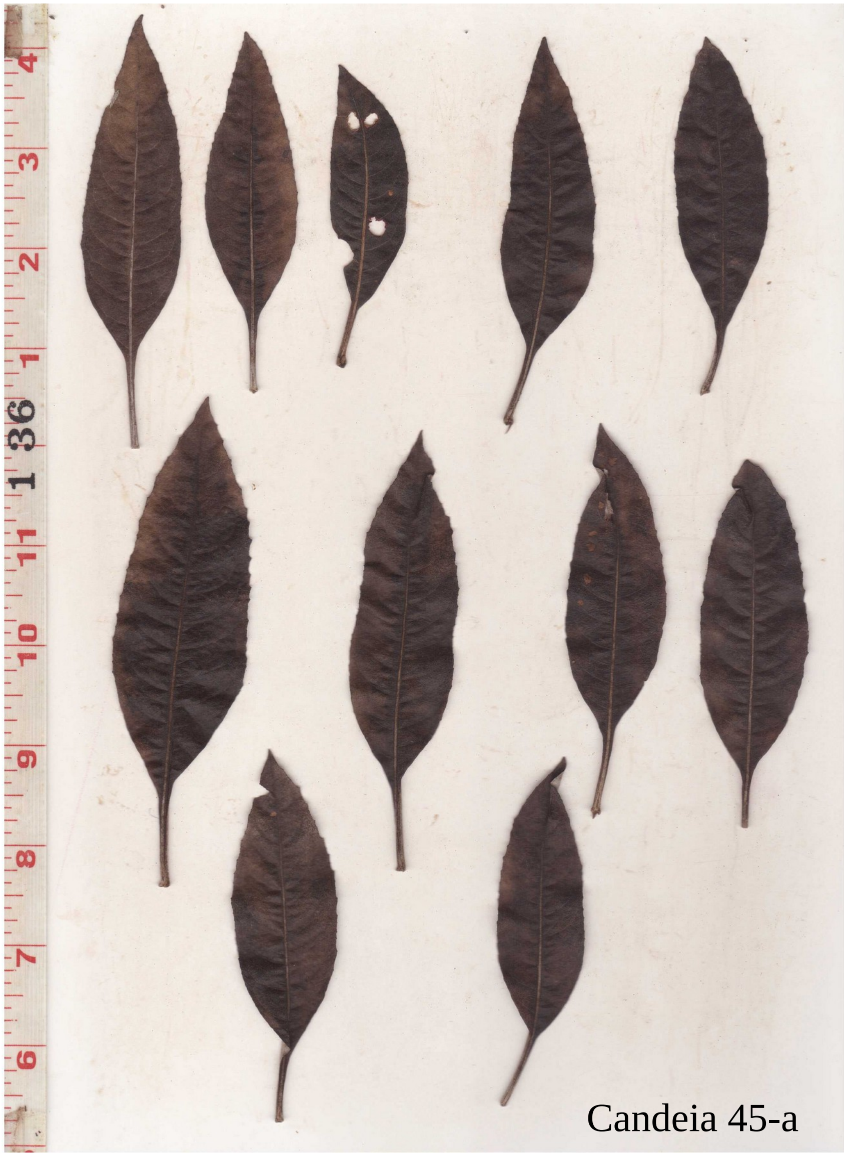

Candeia 45-a

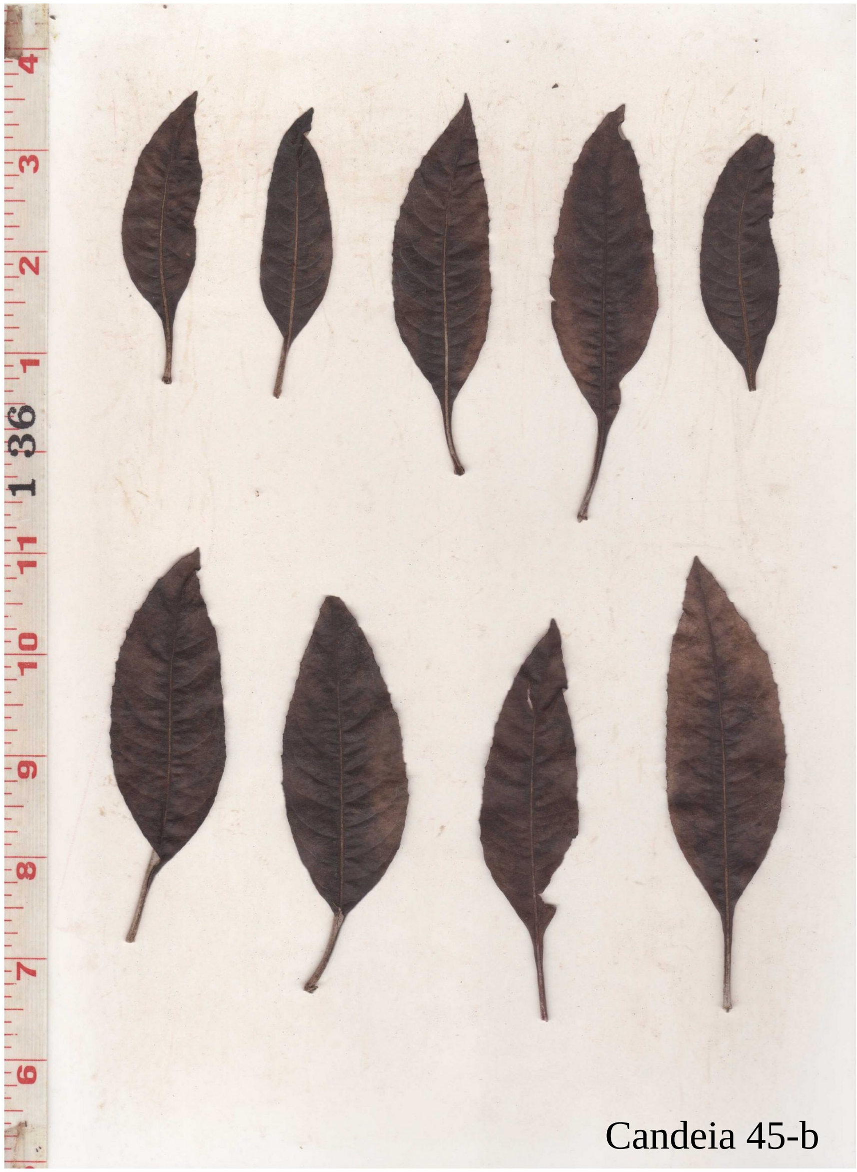

Candeia 45-b

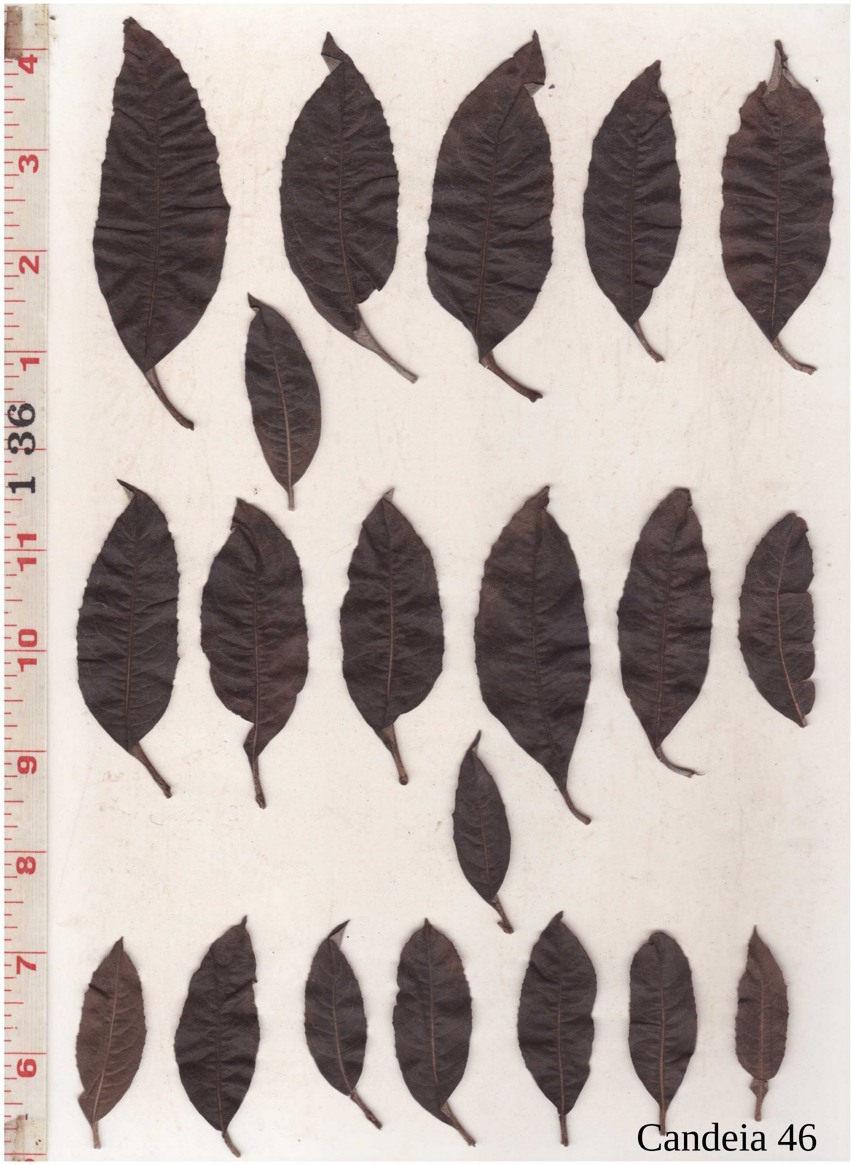
Candeia 46

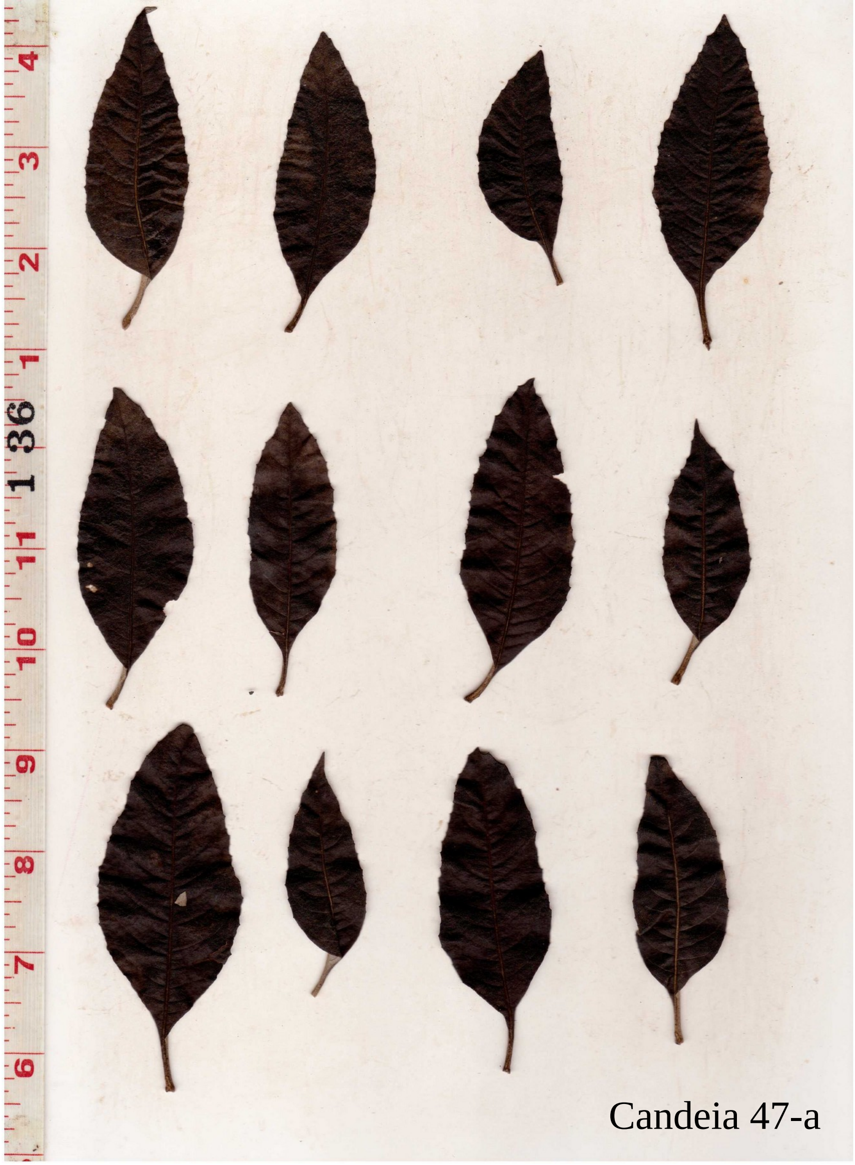
Candeia 47-a

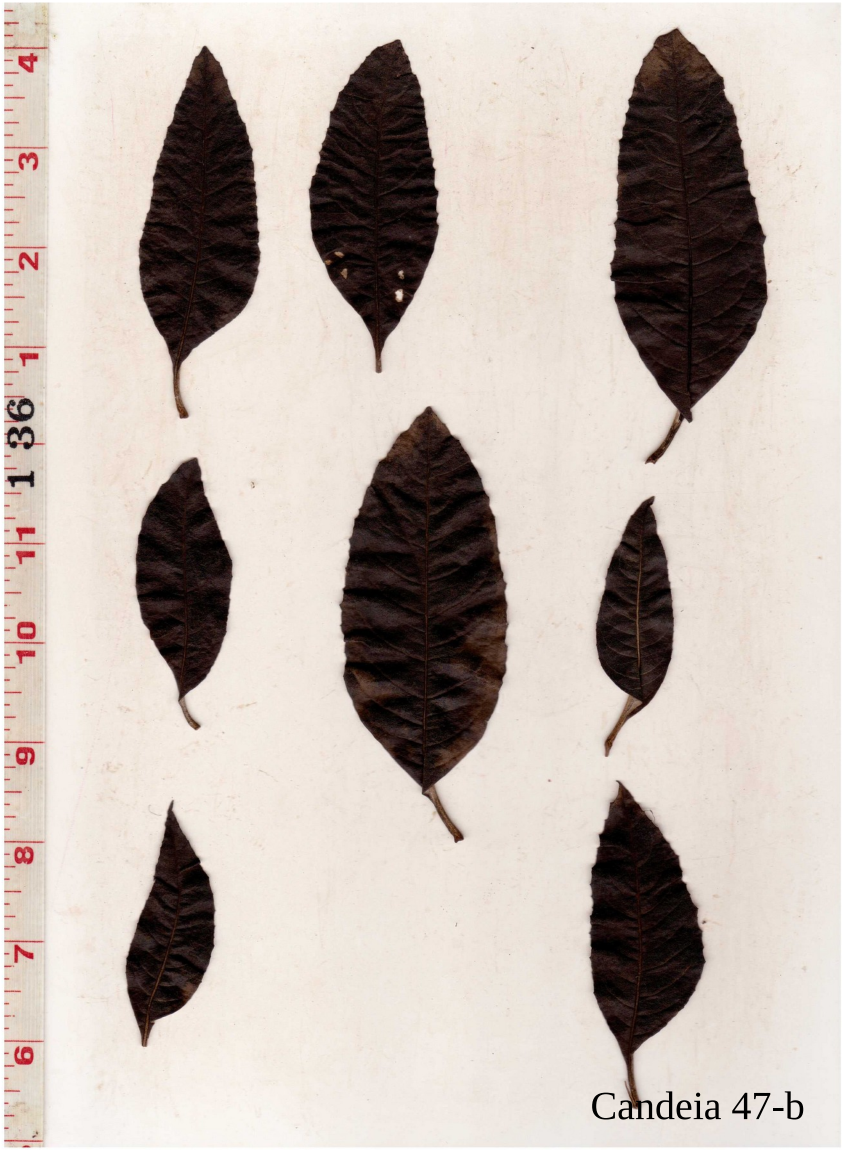
Candeia 47-b

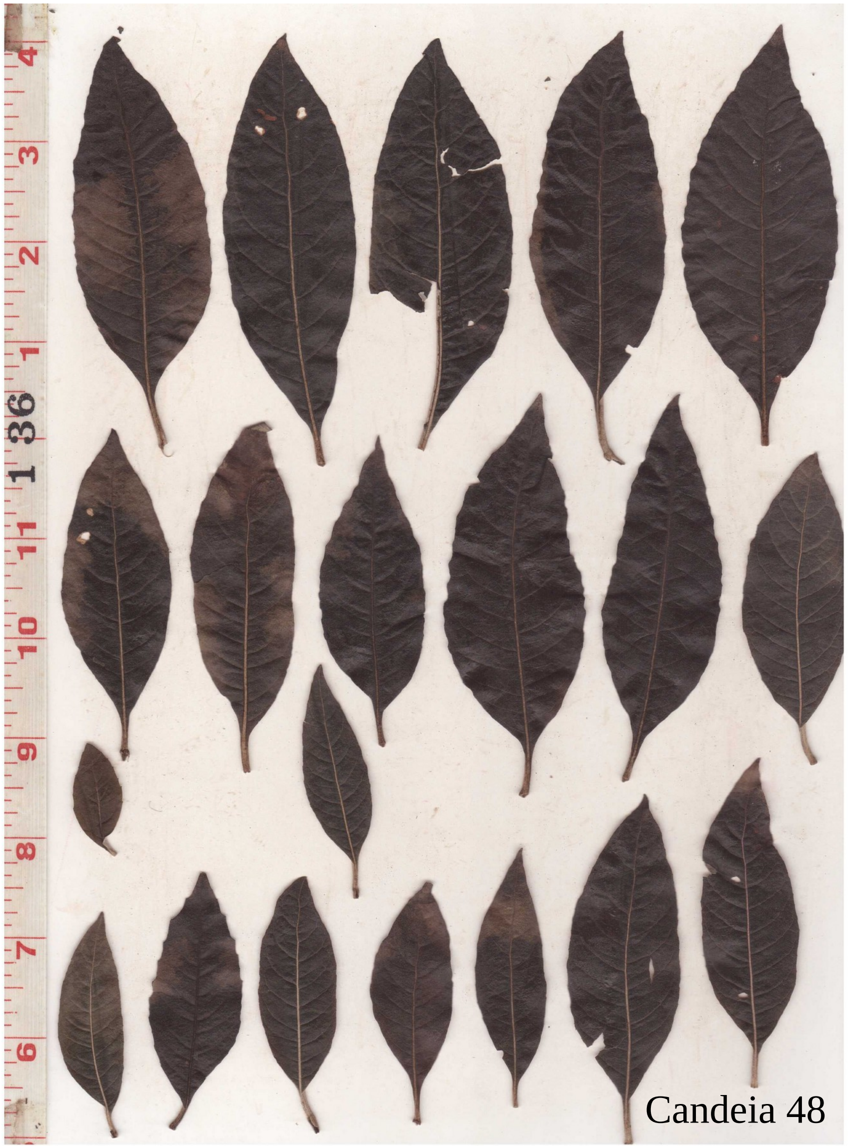
Candeia 48

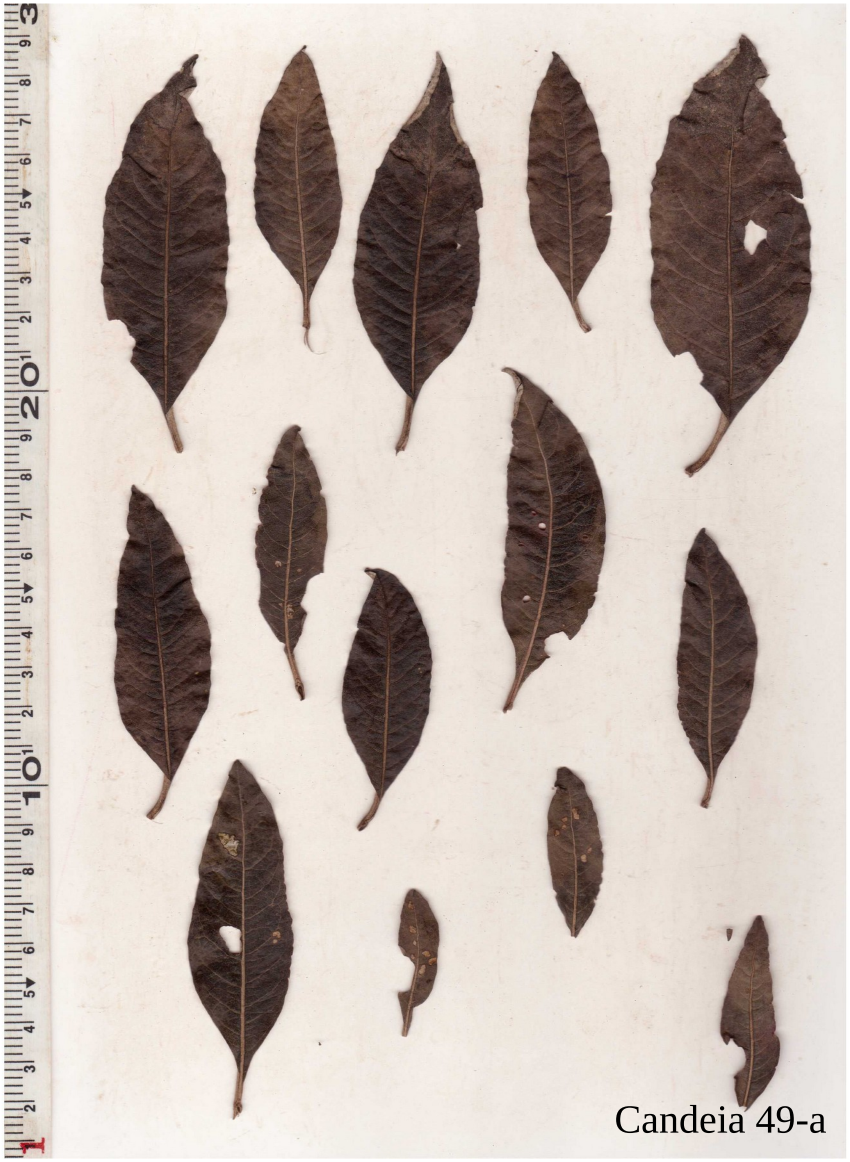

Candeia 49-a

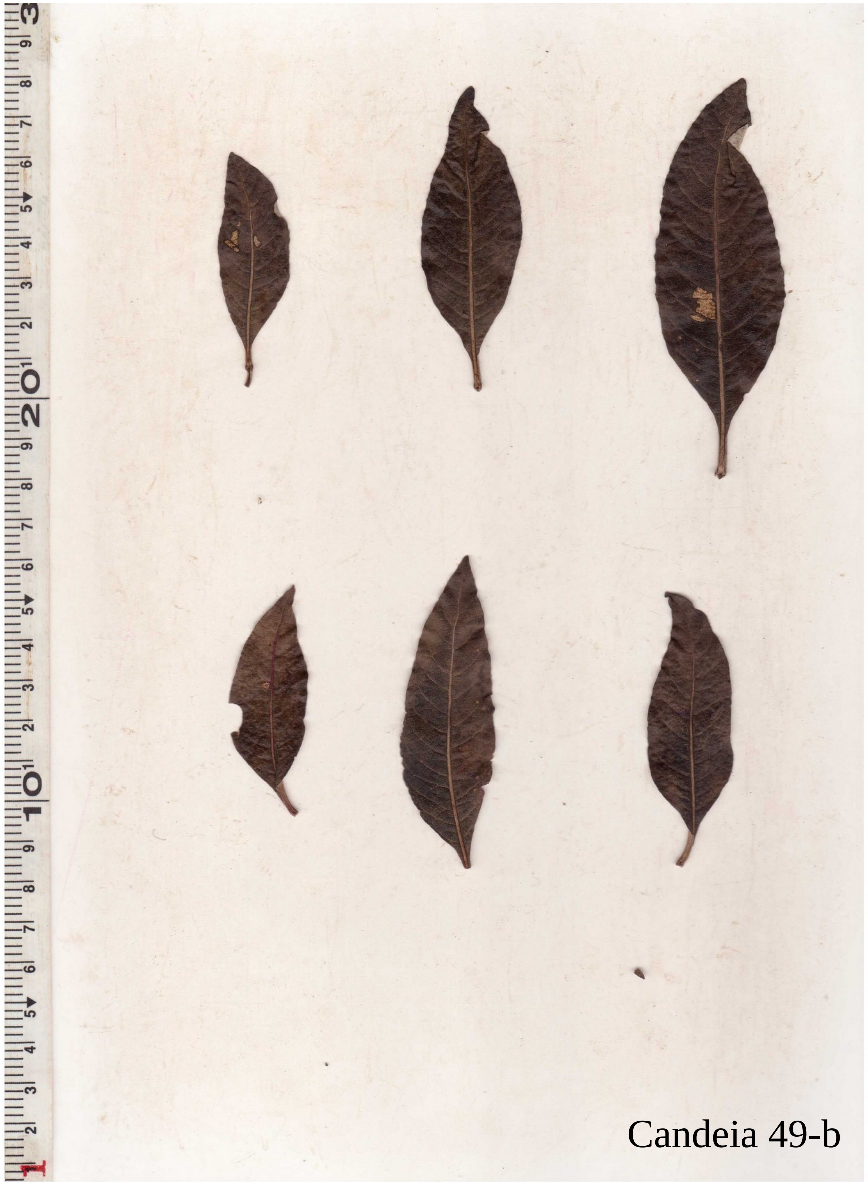

Candeia 49-b

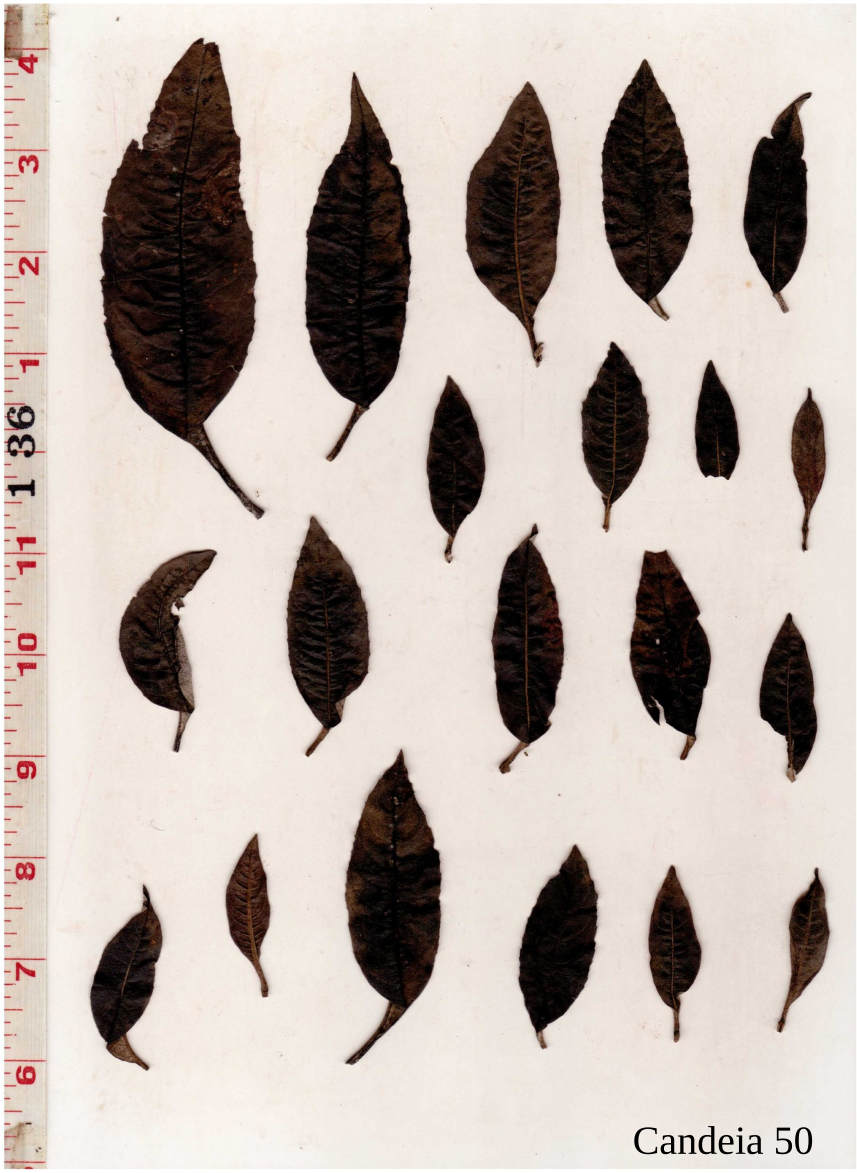

Candeia 50

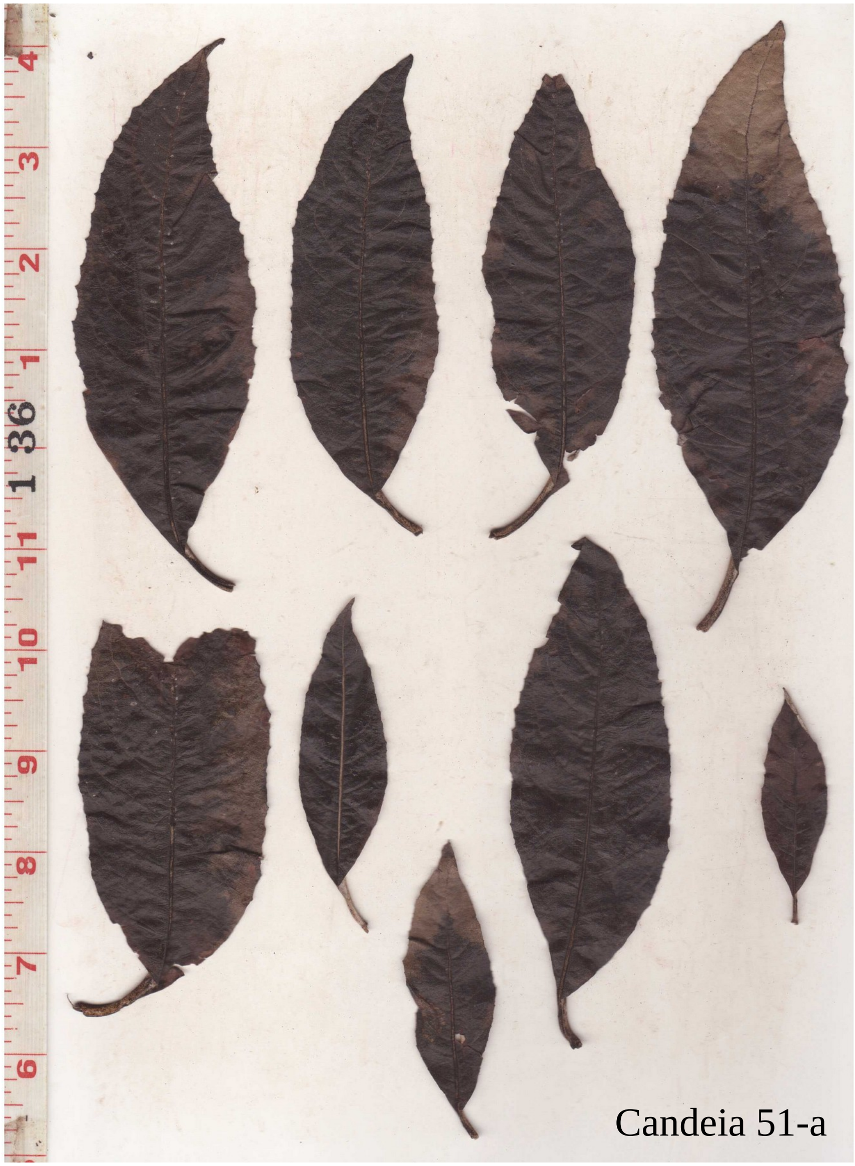

Candeia 51-a

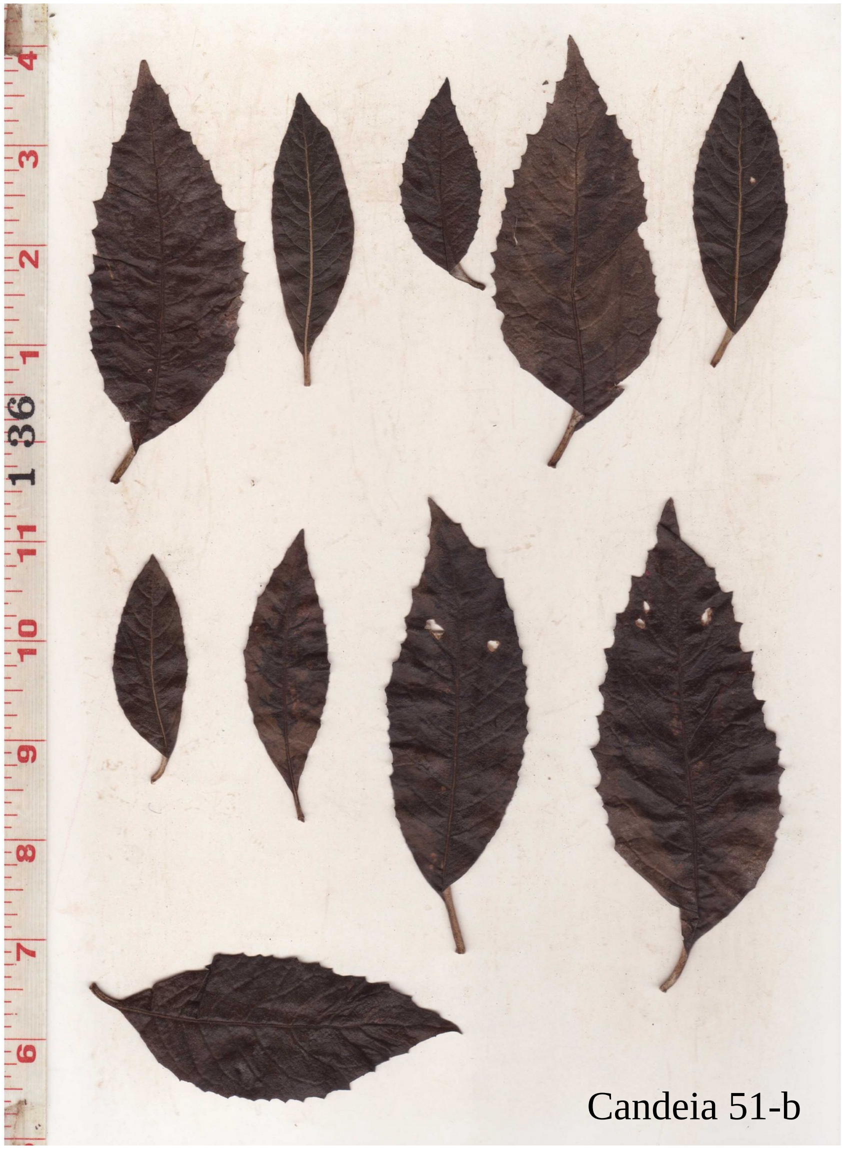
Candeia 51-b

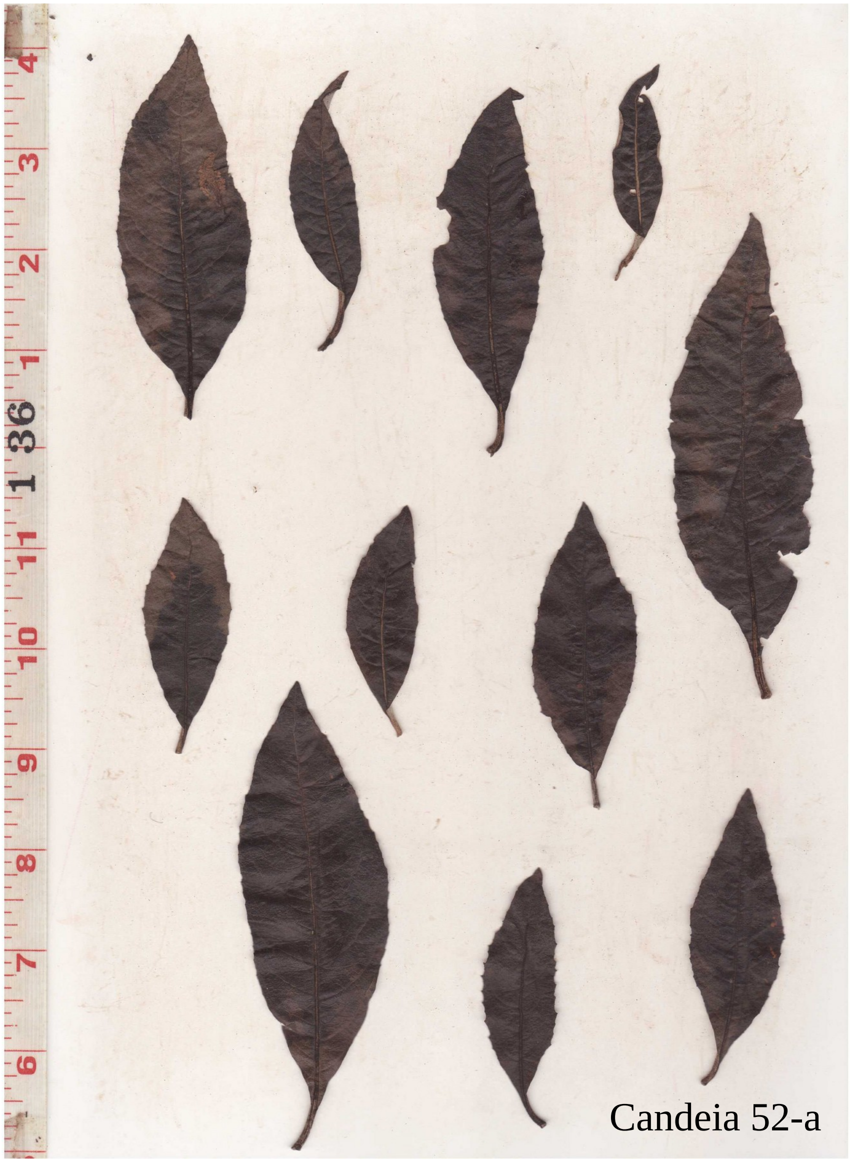
Candeia 52-a

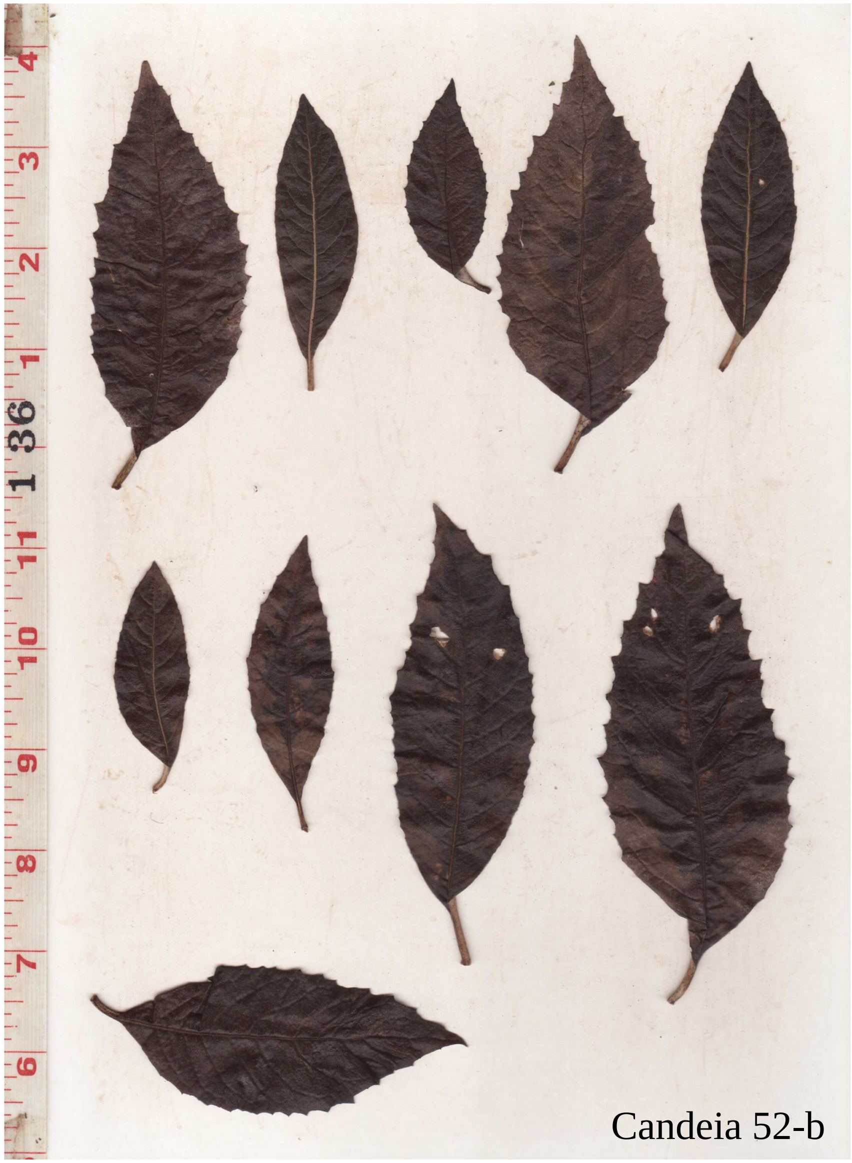

Candeia 52-b

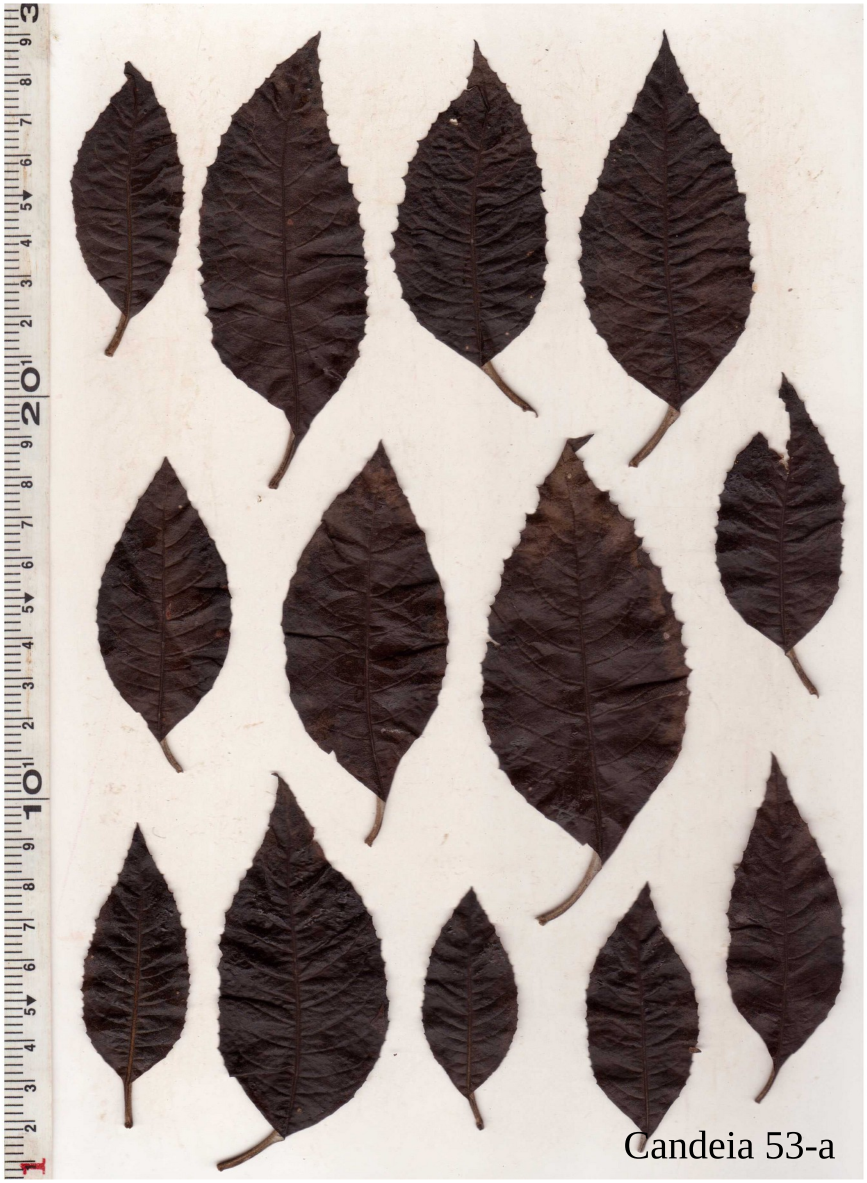

Candeia 53-a

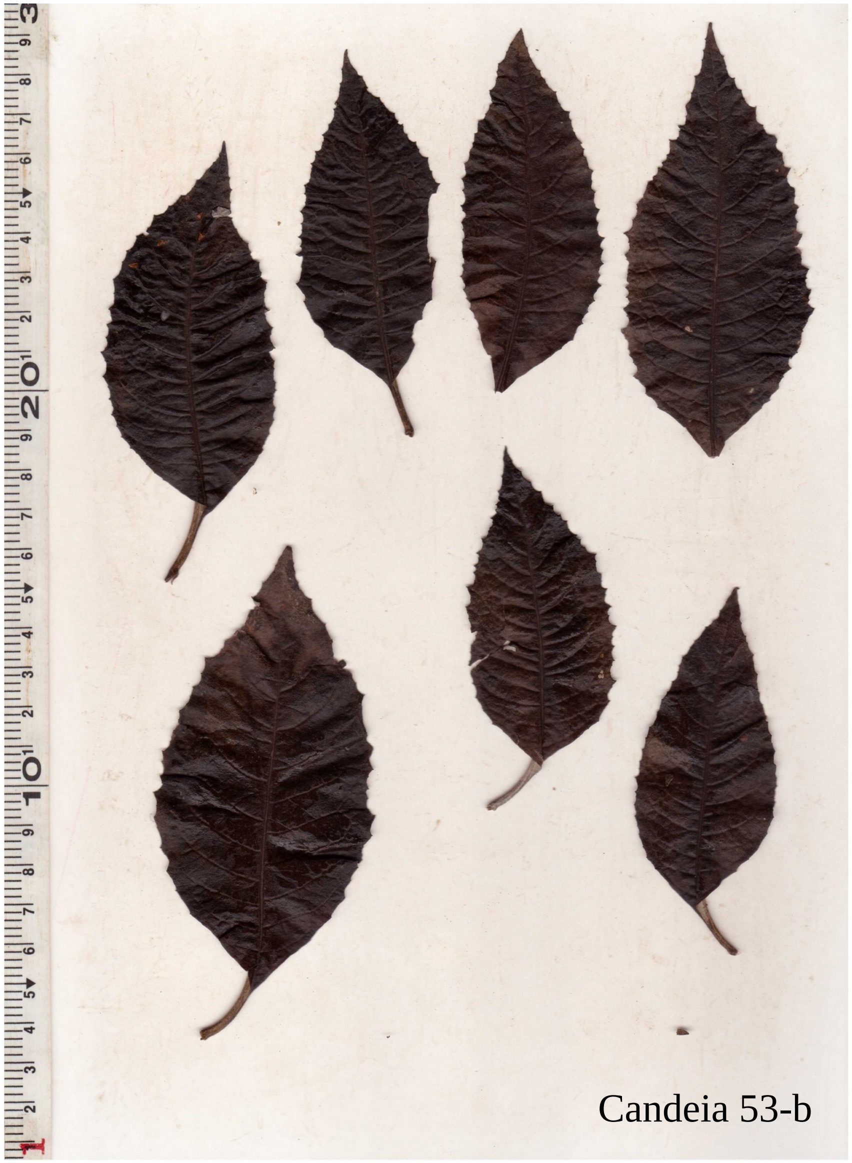
Candeia 53-b

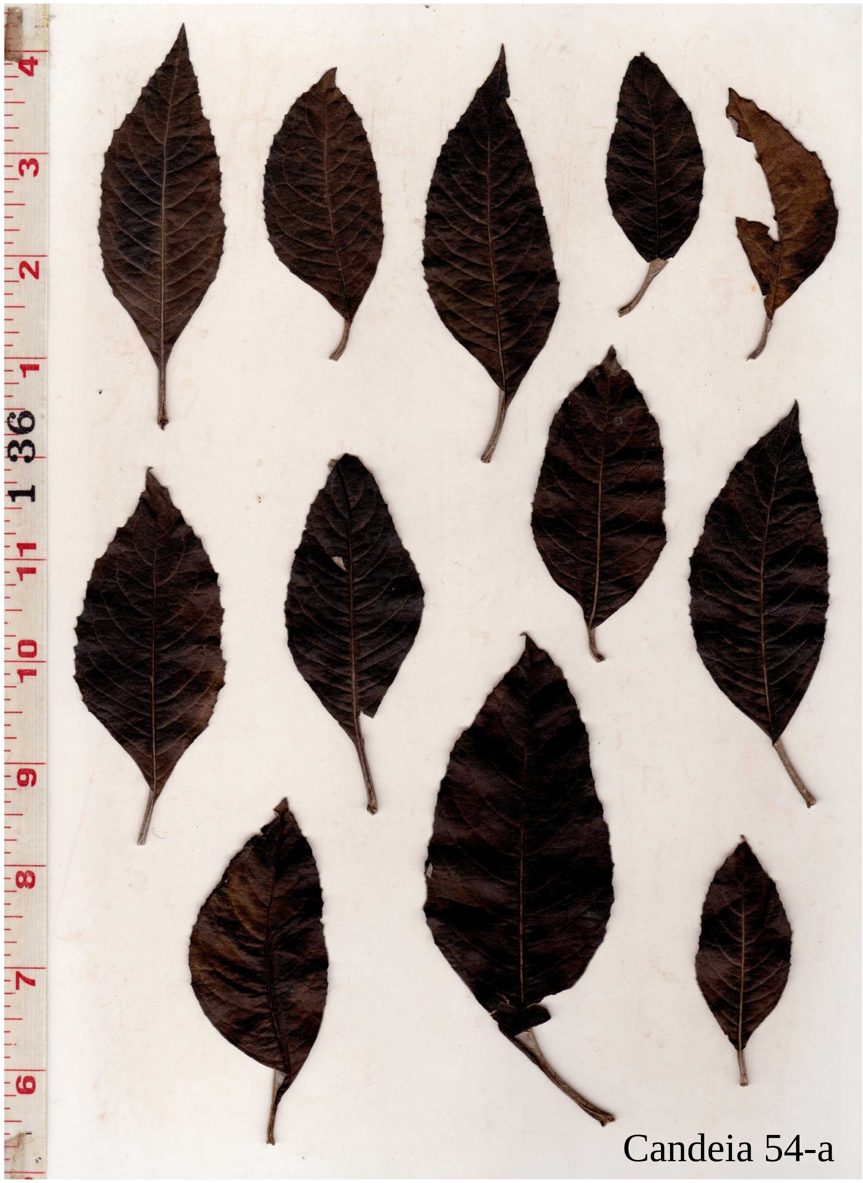
Candeia 54-a

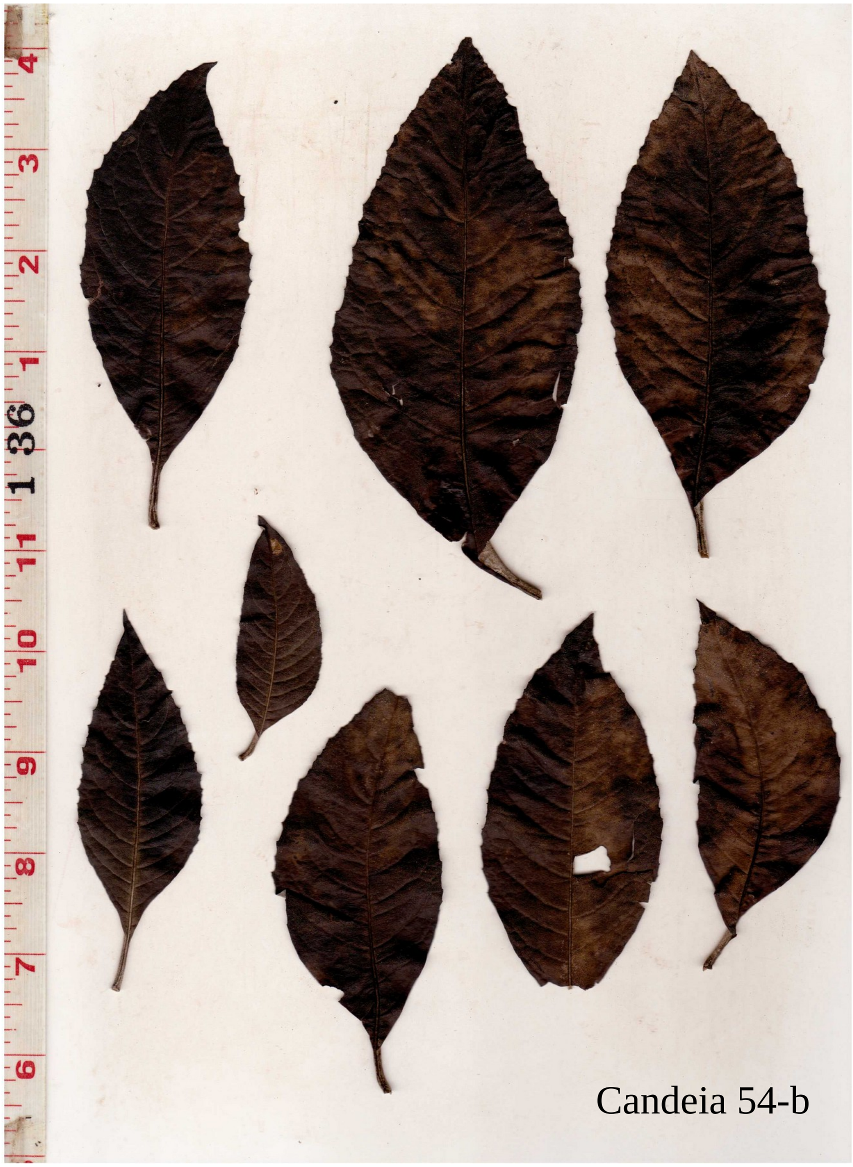

Candeia 54-b

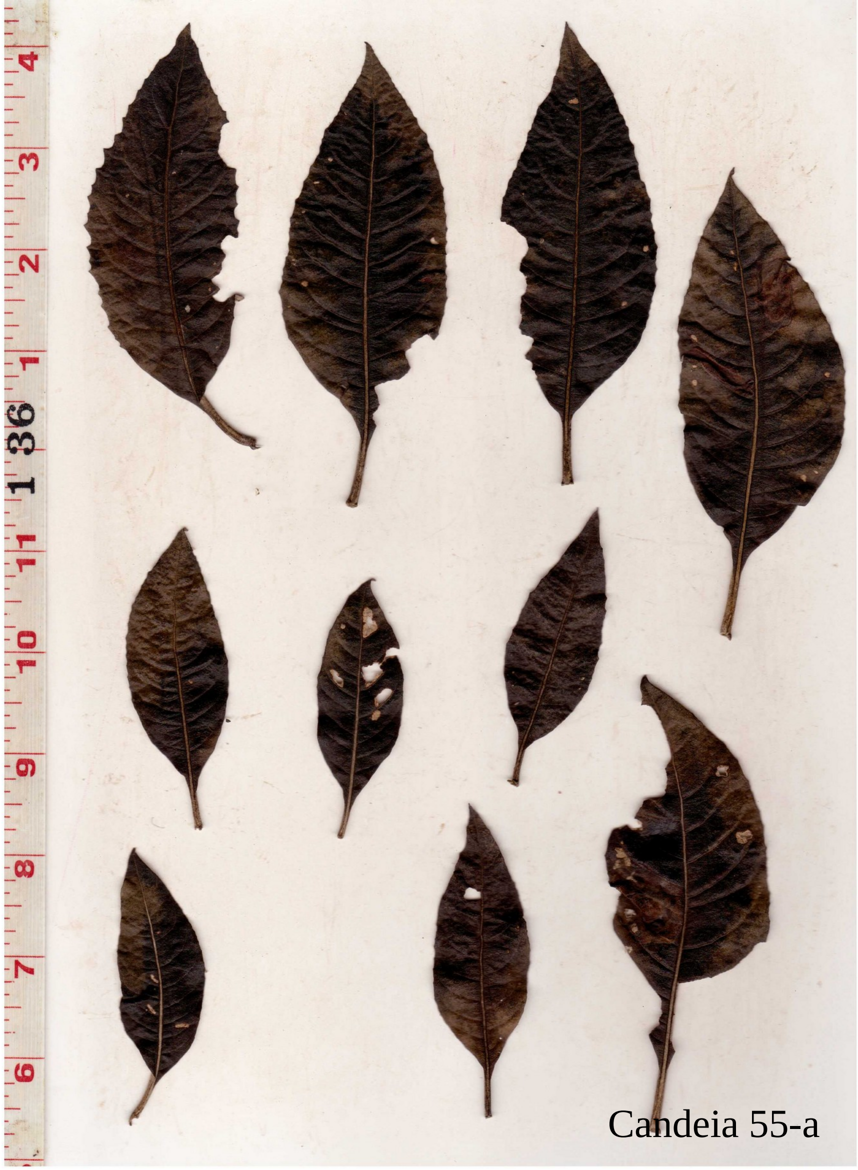

Candeia 55-a

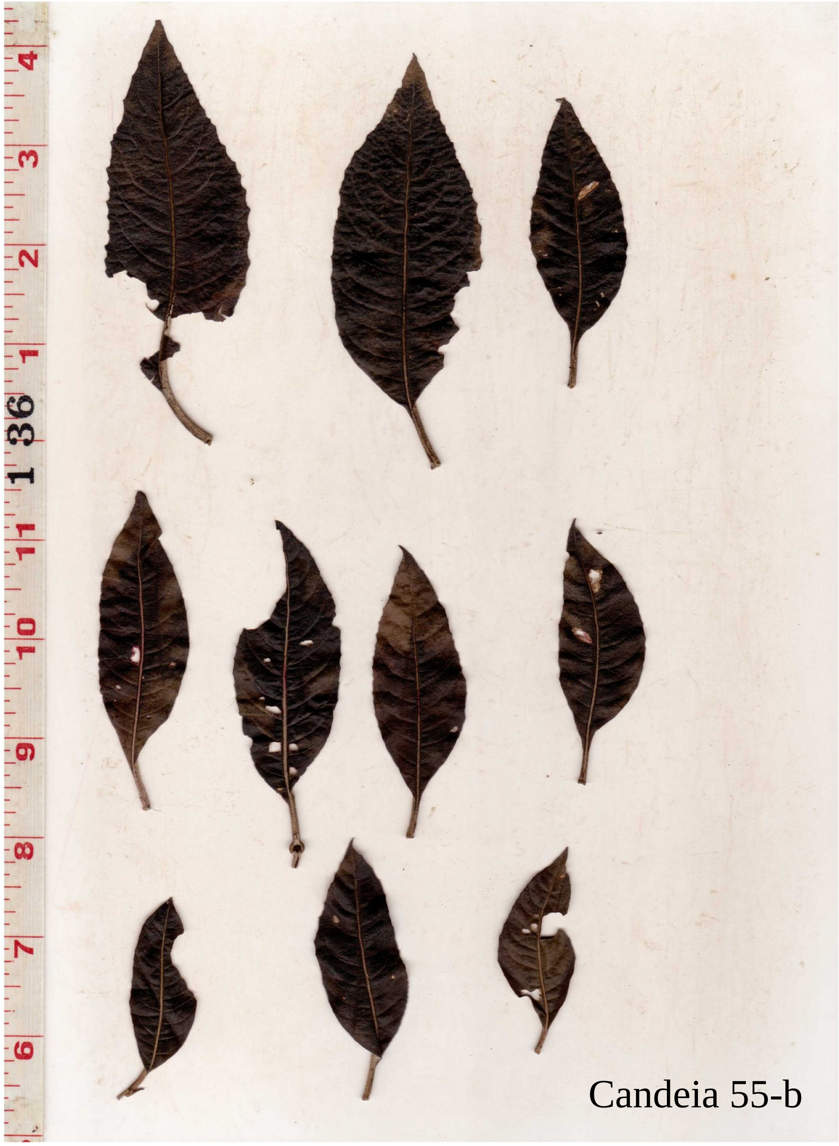
Candeia 55-b

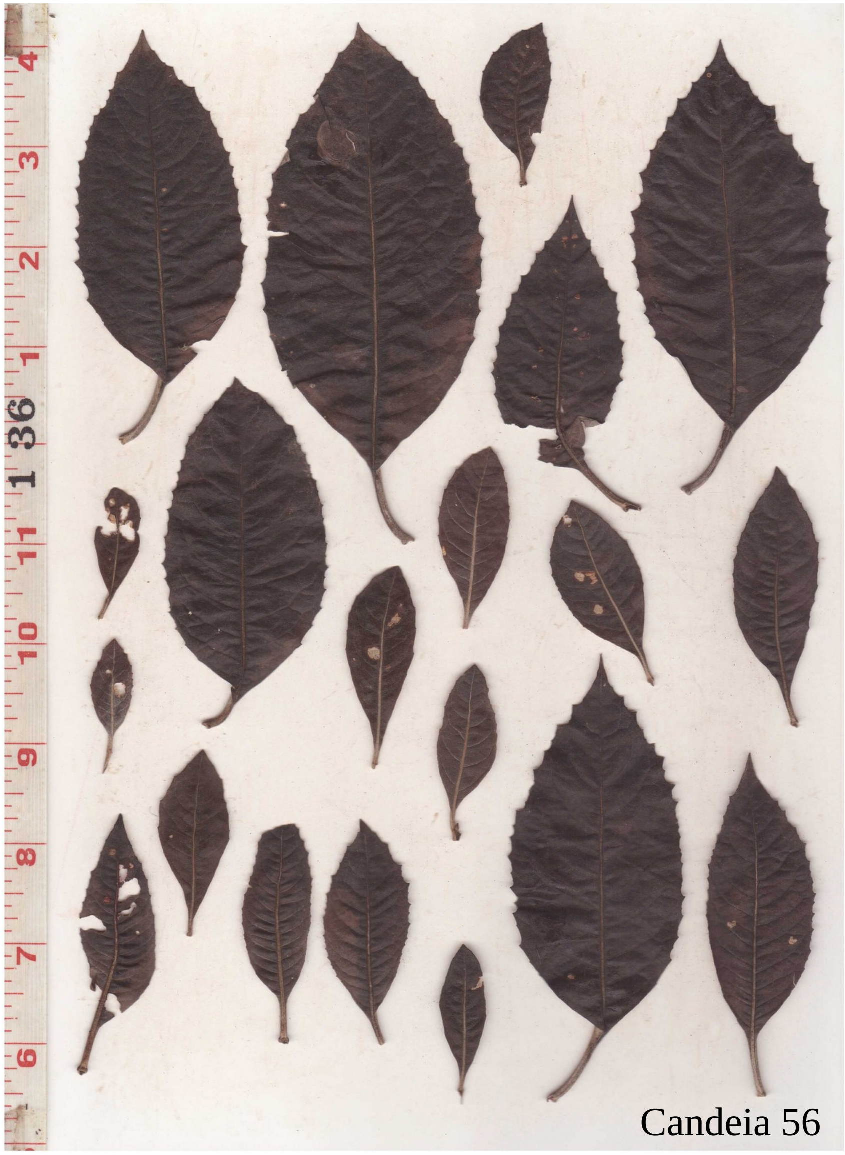
Candeia 56

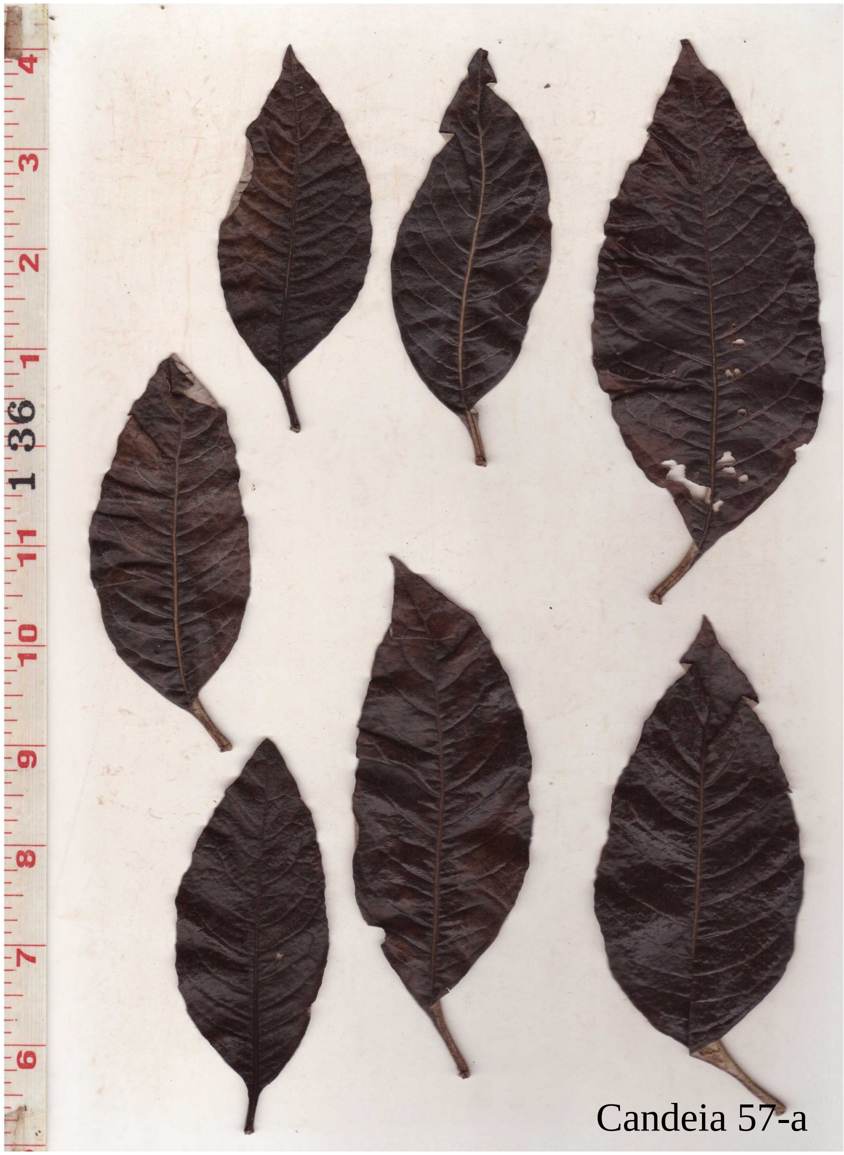

Candeia 57-a

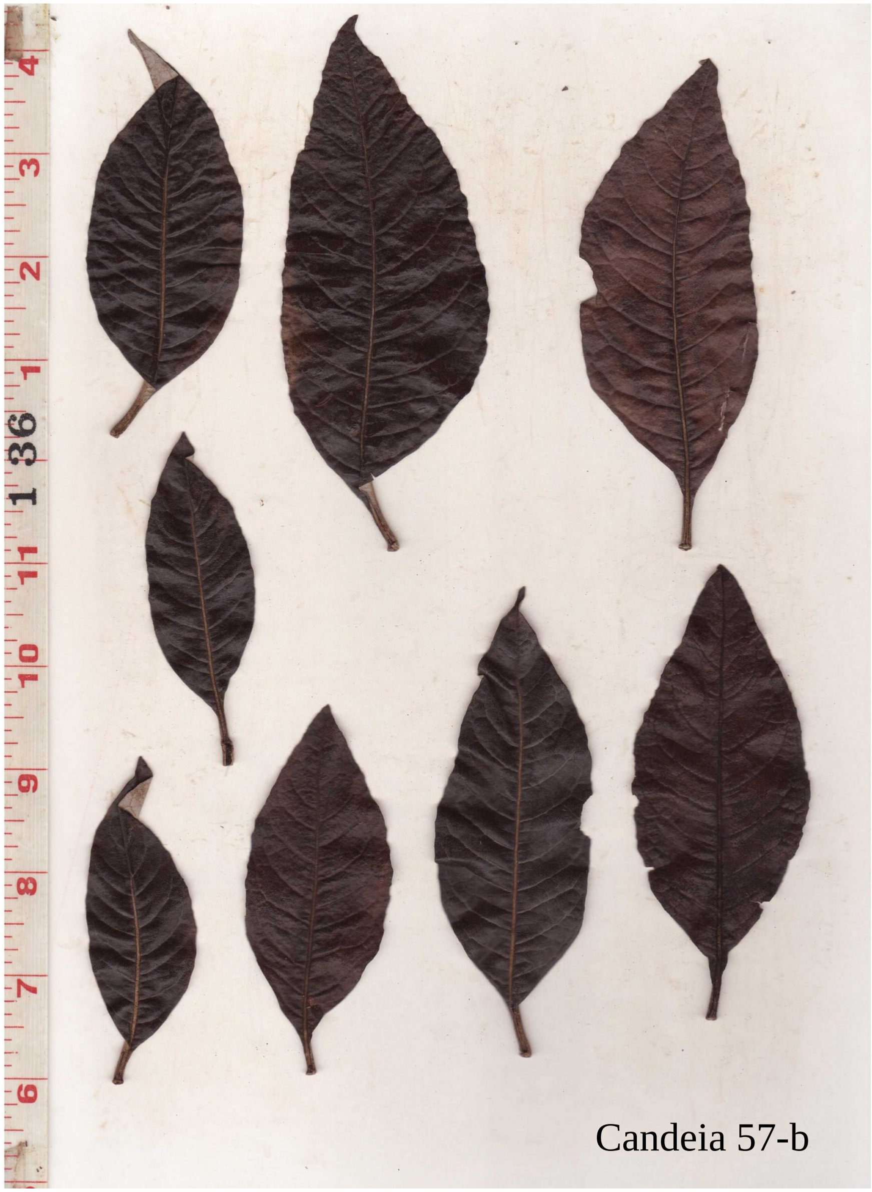

Candeia 57-b

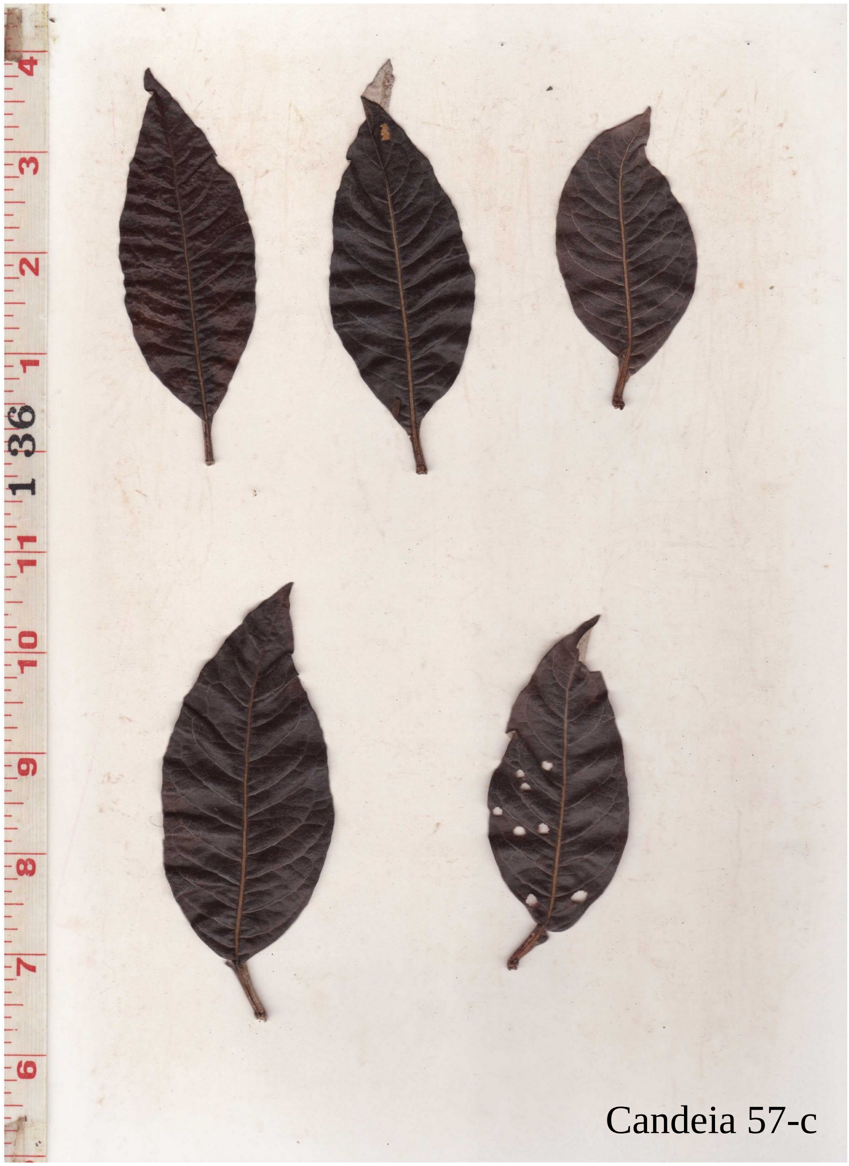

Candeia 57-c

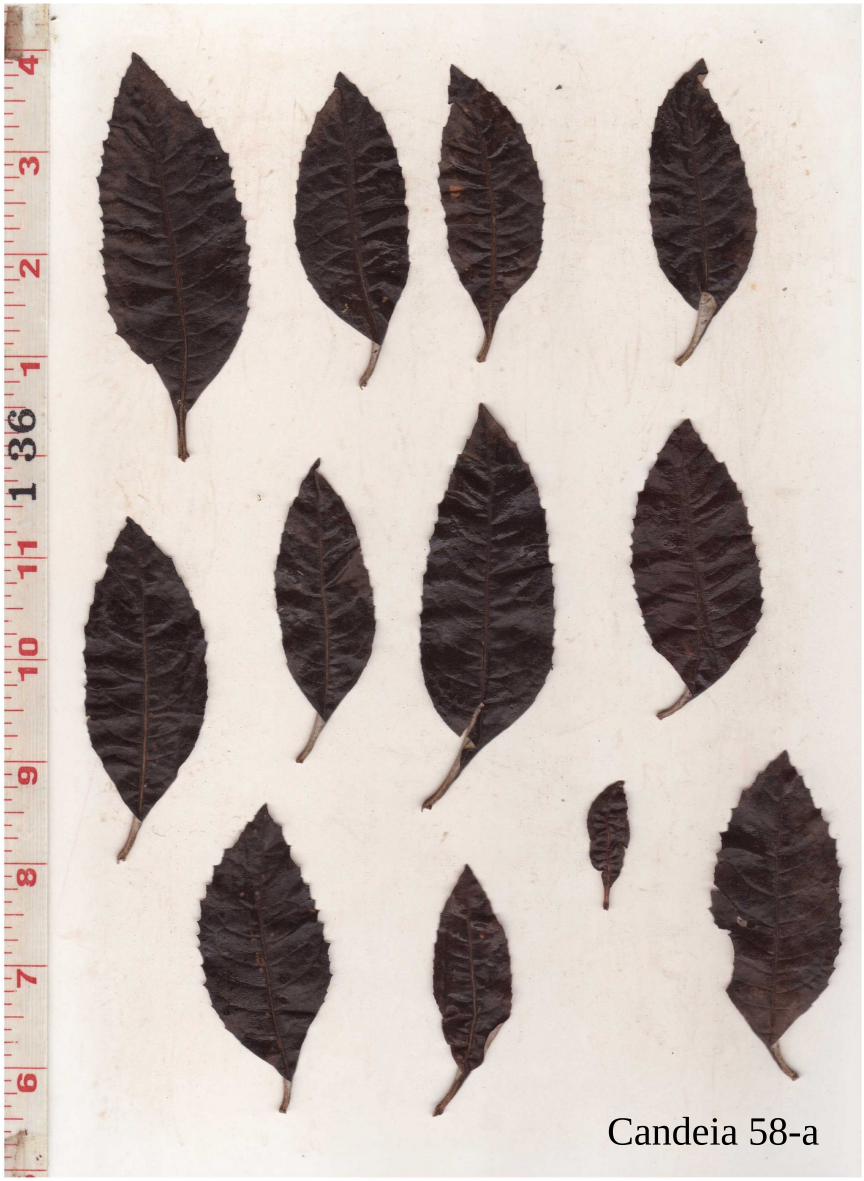

Candeia 58-a

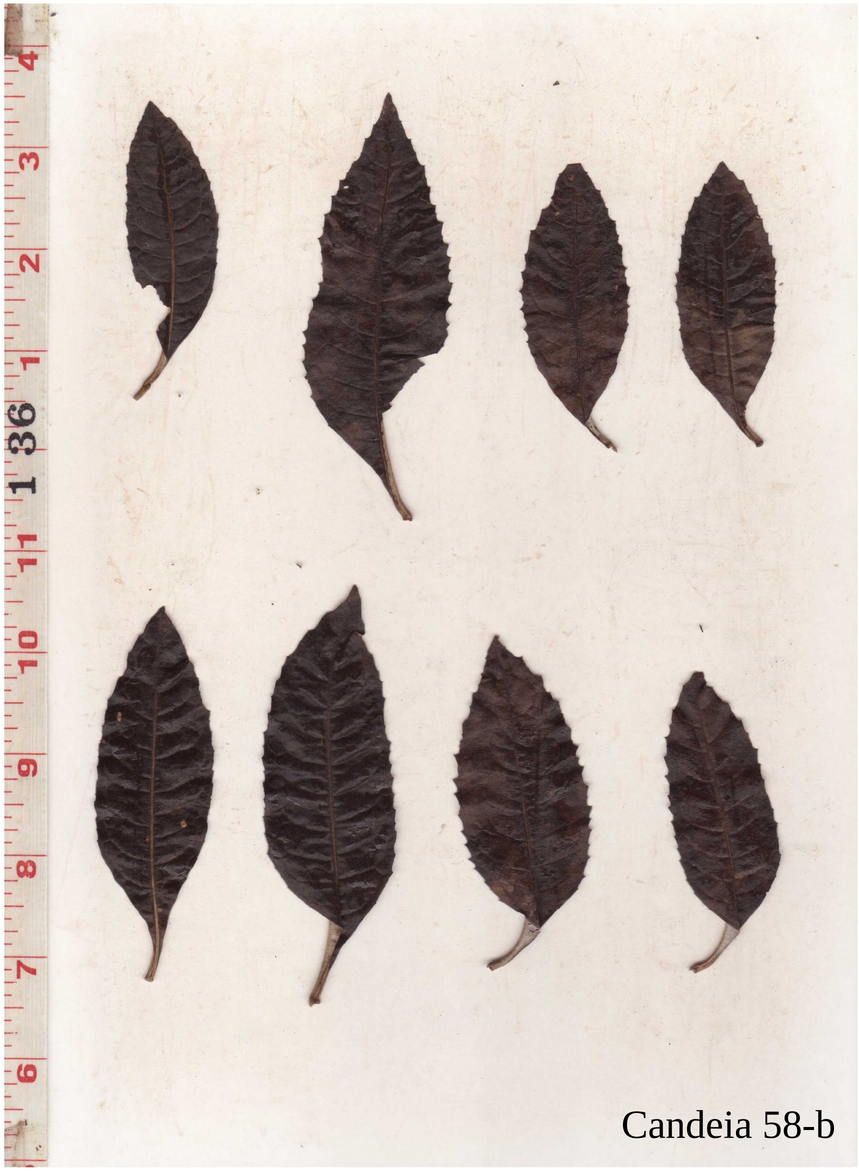
Candeia 58-b

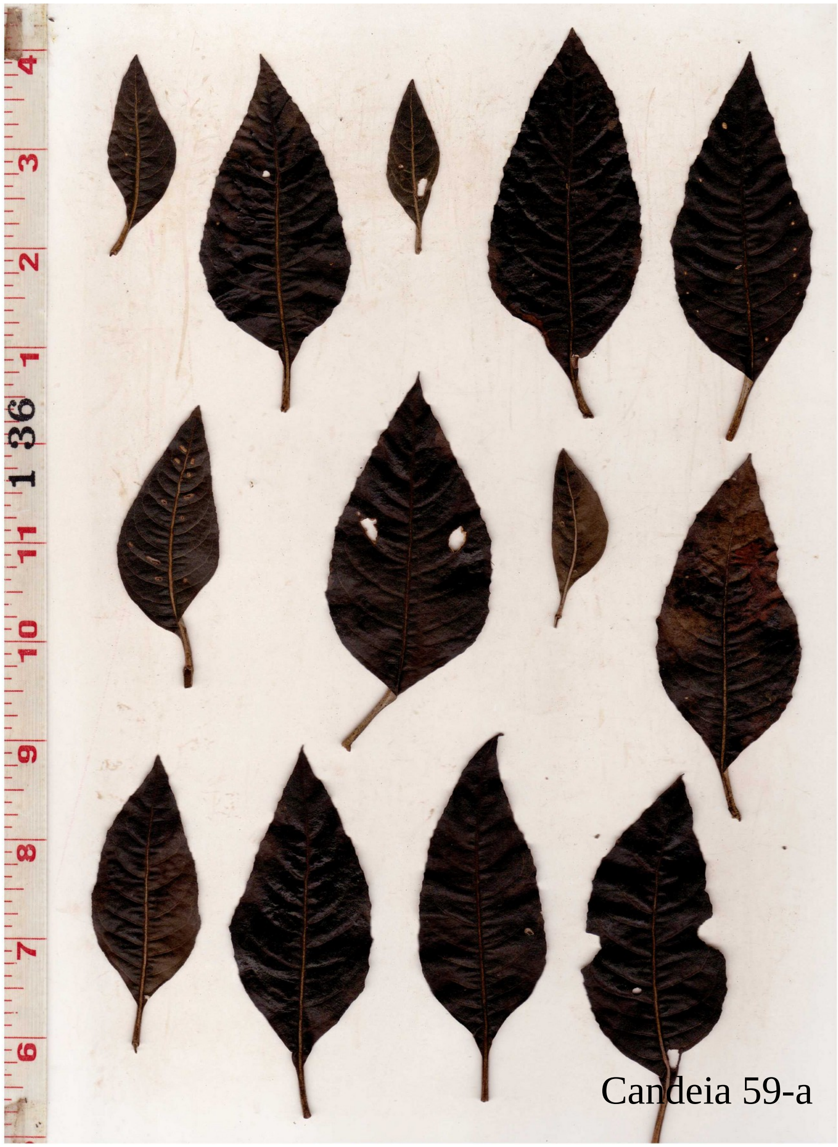
Candeia 59-a

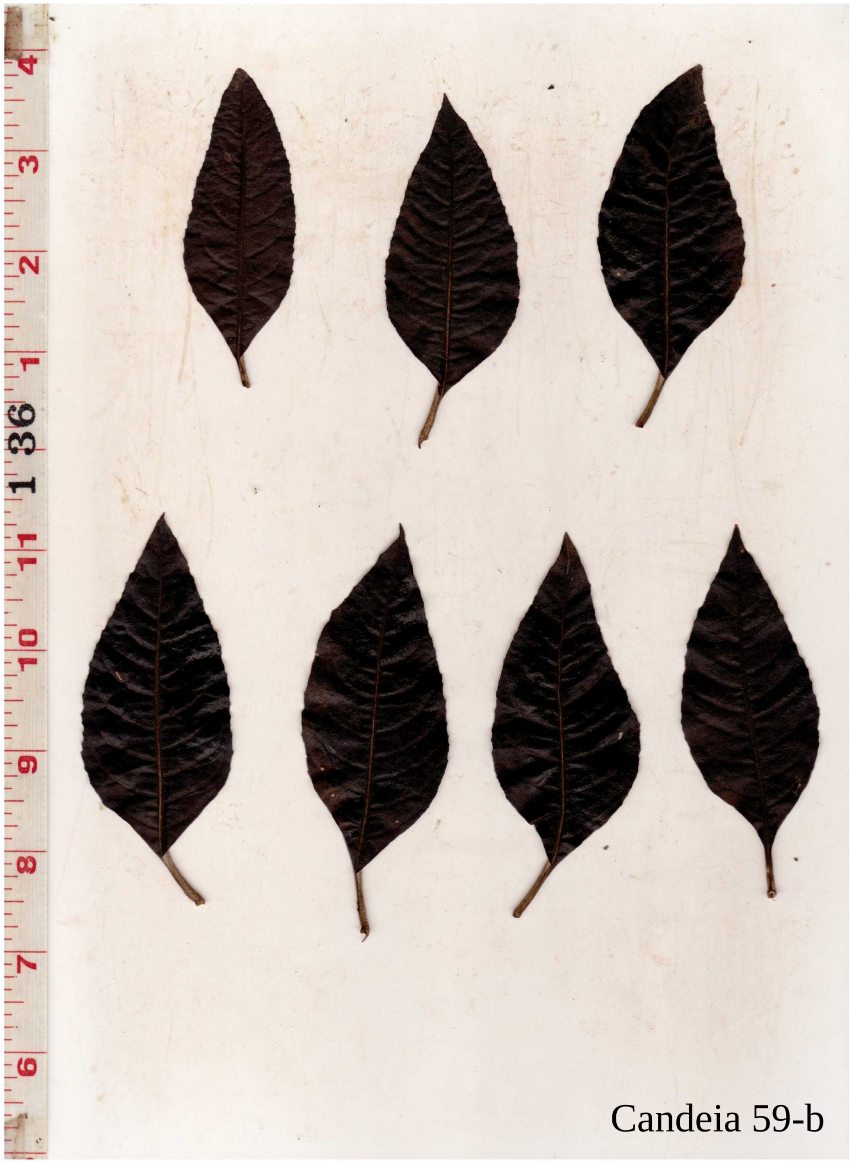

Candeia 59-b

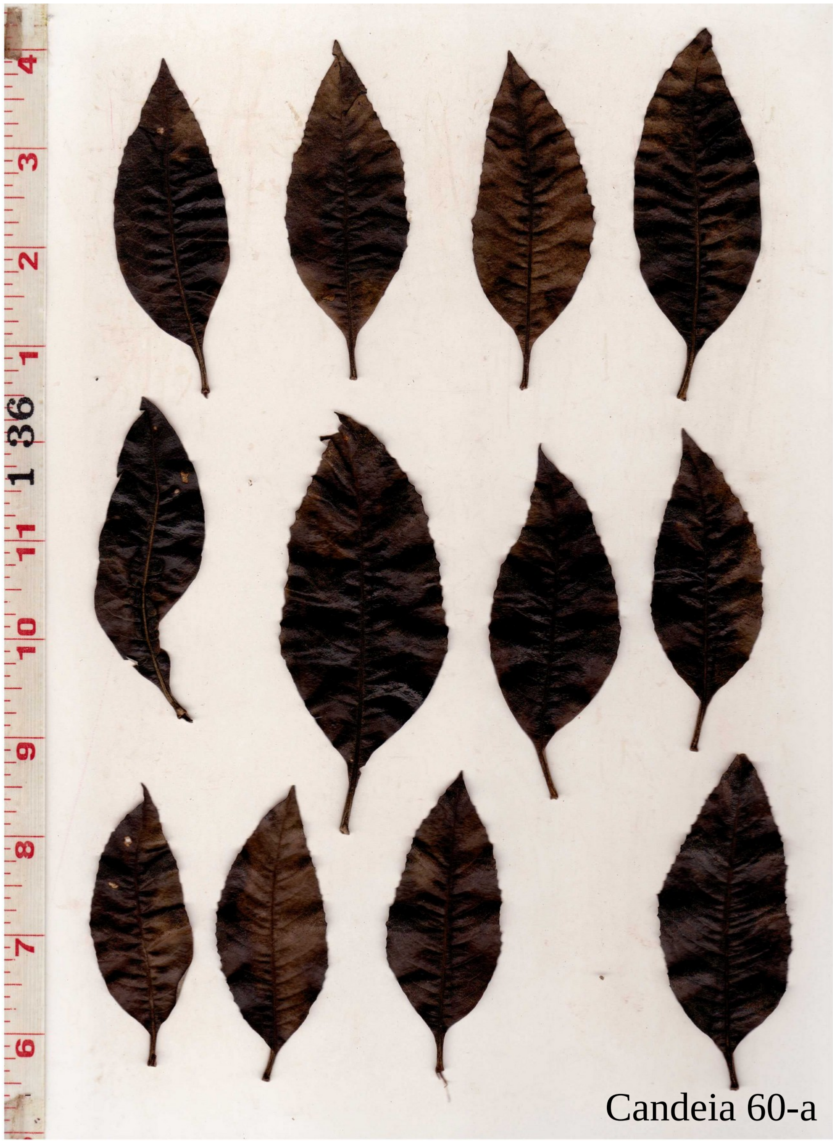

Candeia 60-a

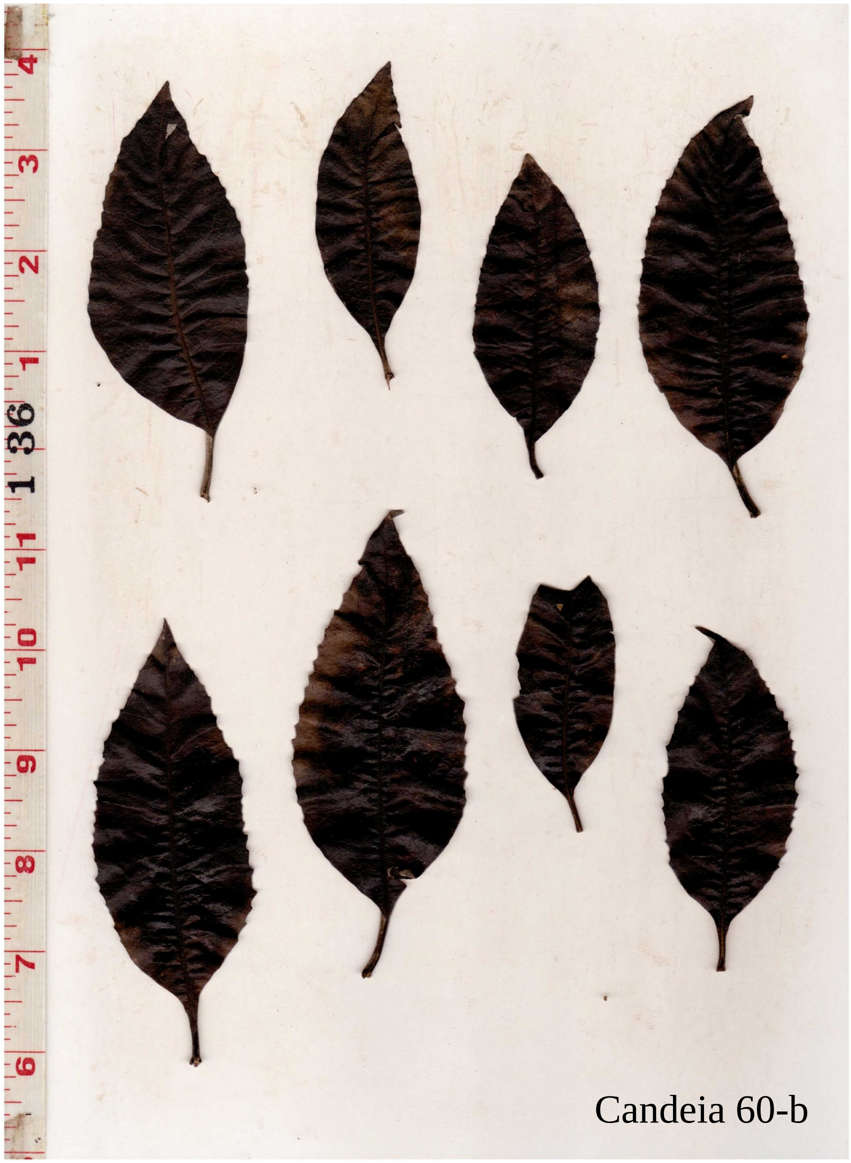

Candeia 60-b

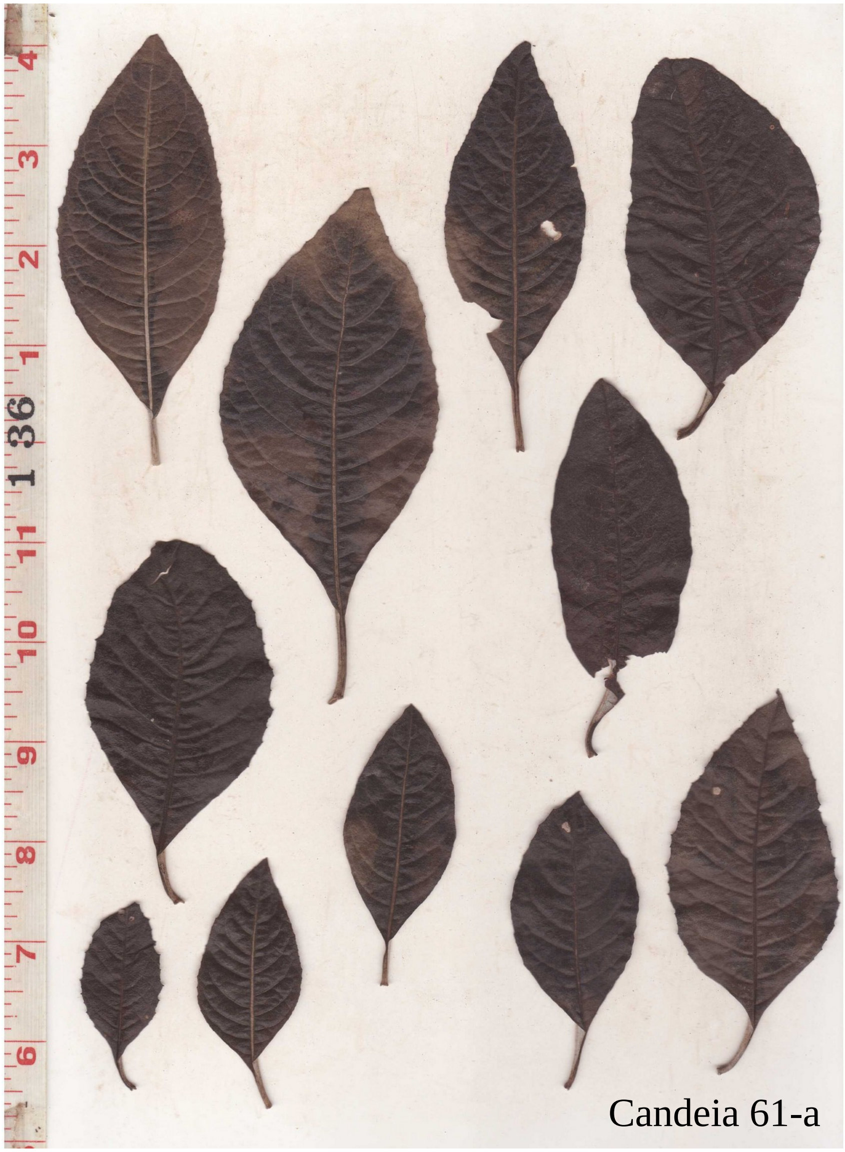

Candeia 61-a

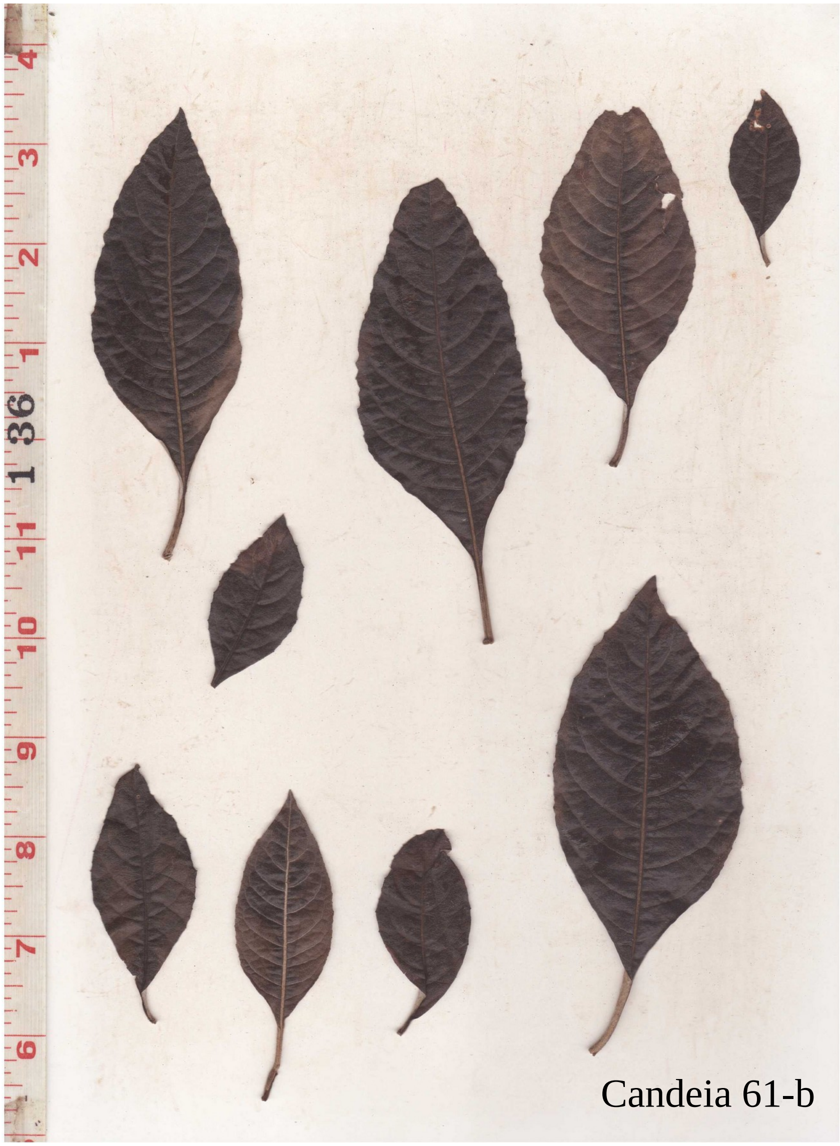

Candeia 61-b

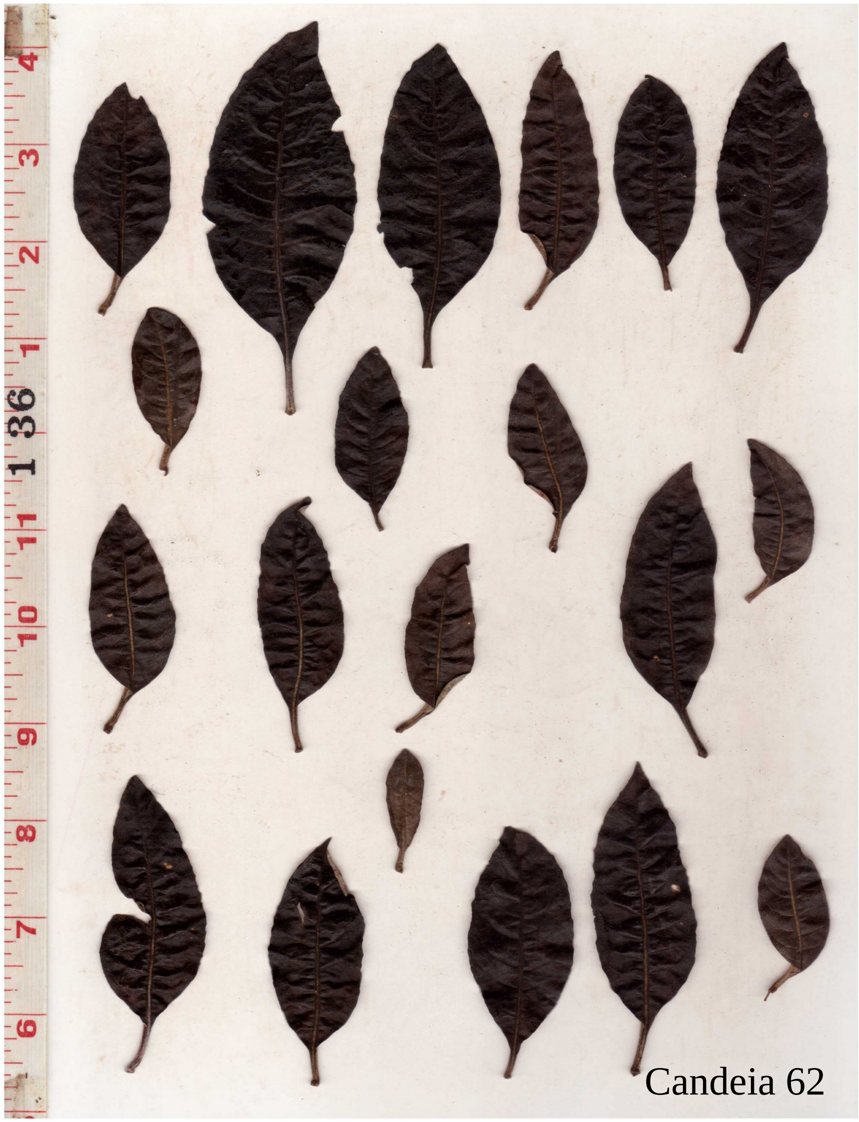

Candeia 62

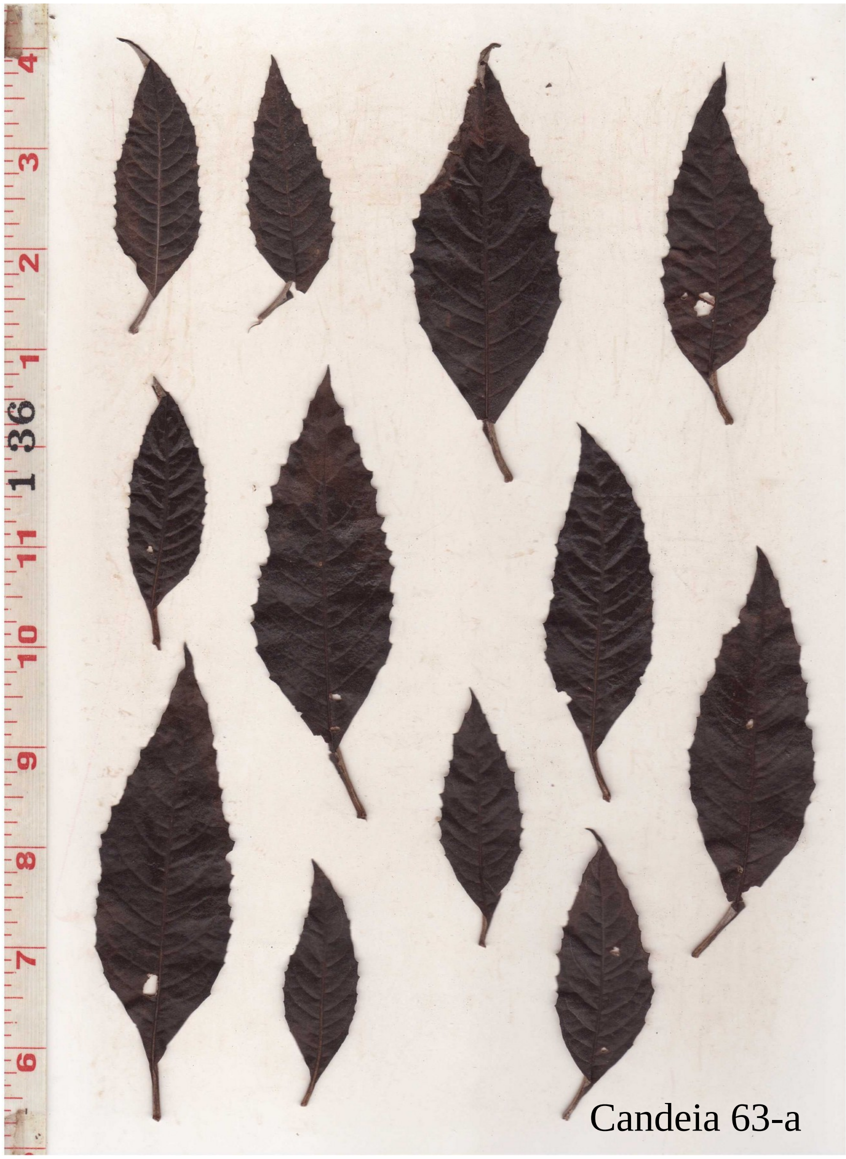

Candeia 63-a

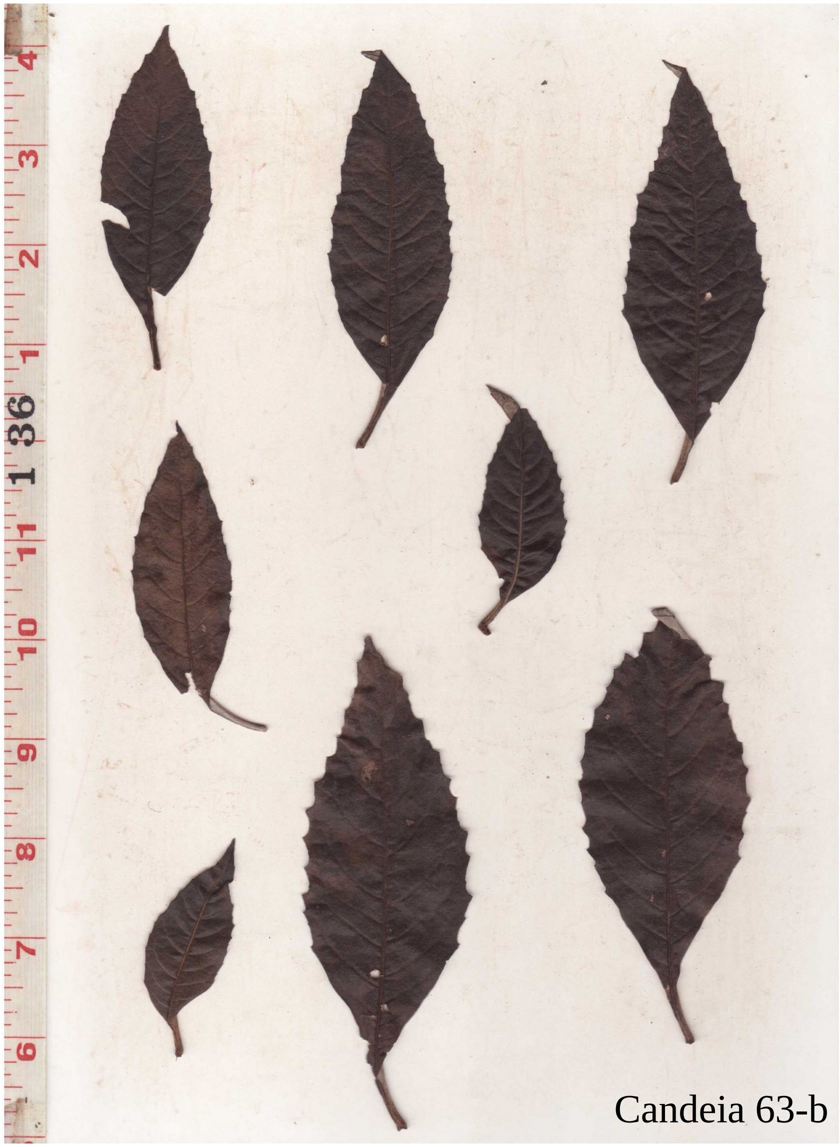

Candeia 63-b

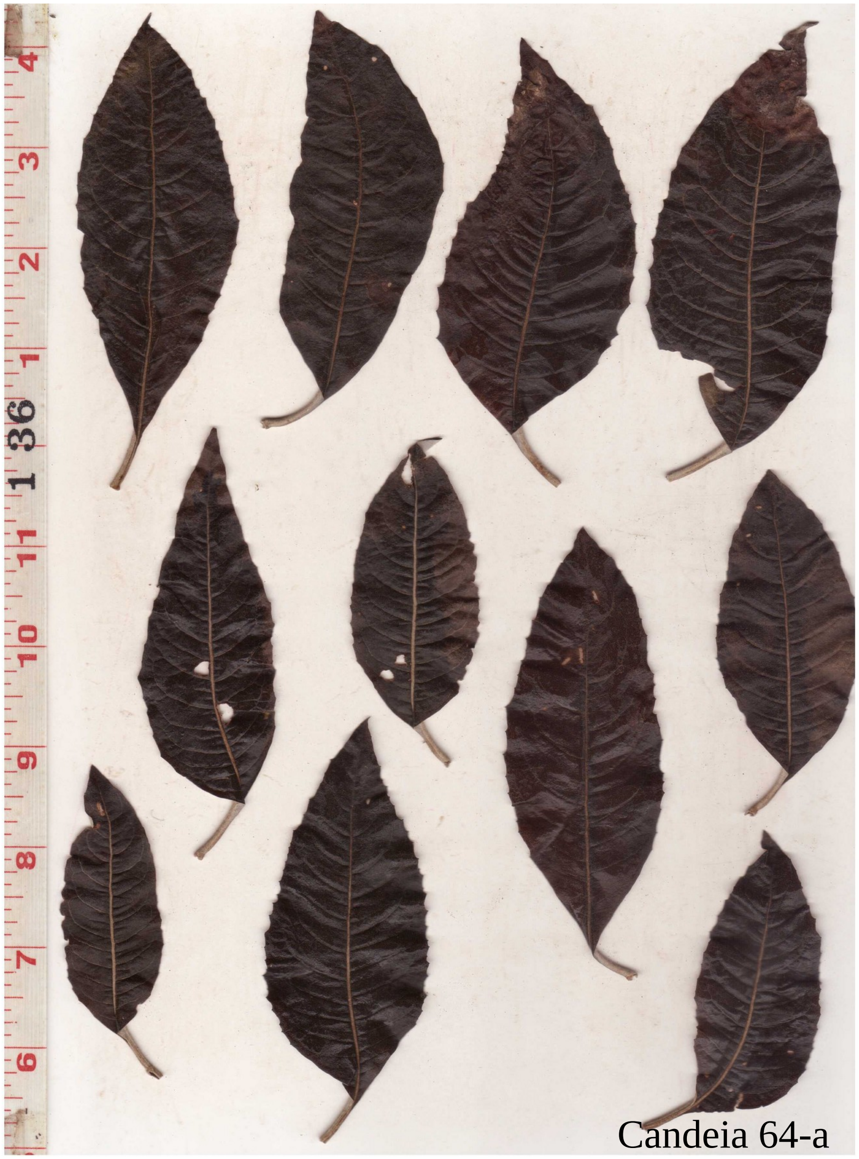

Candeia 64-a

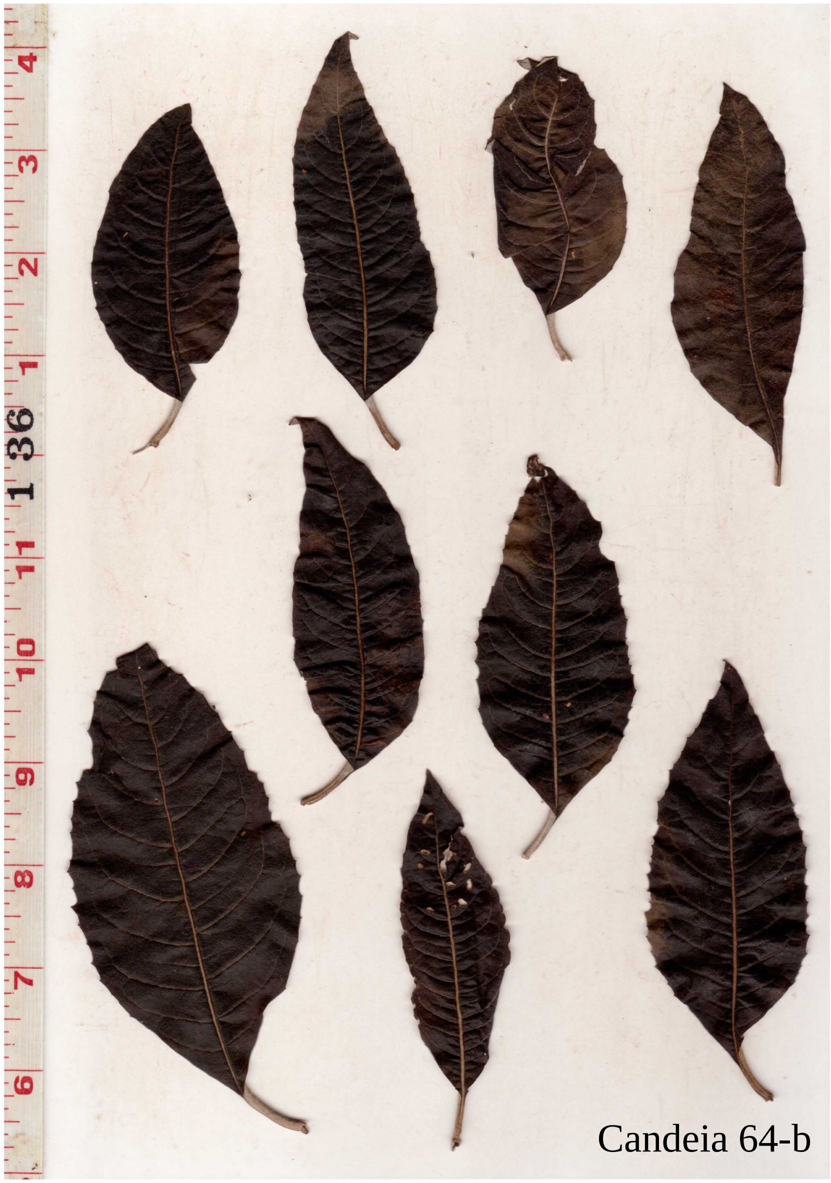

Candeia 64-b

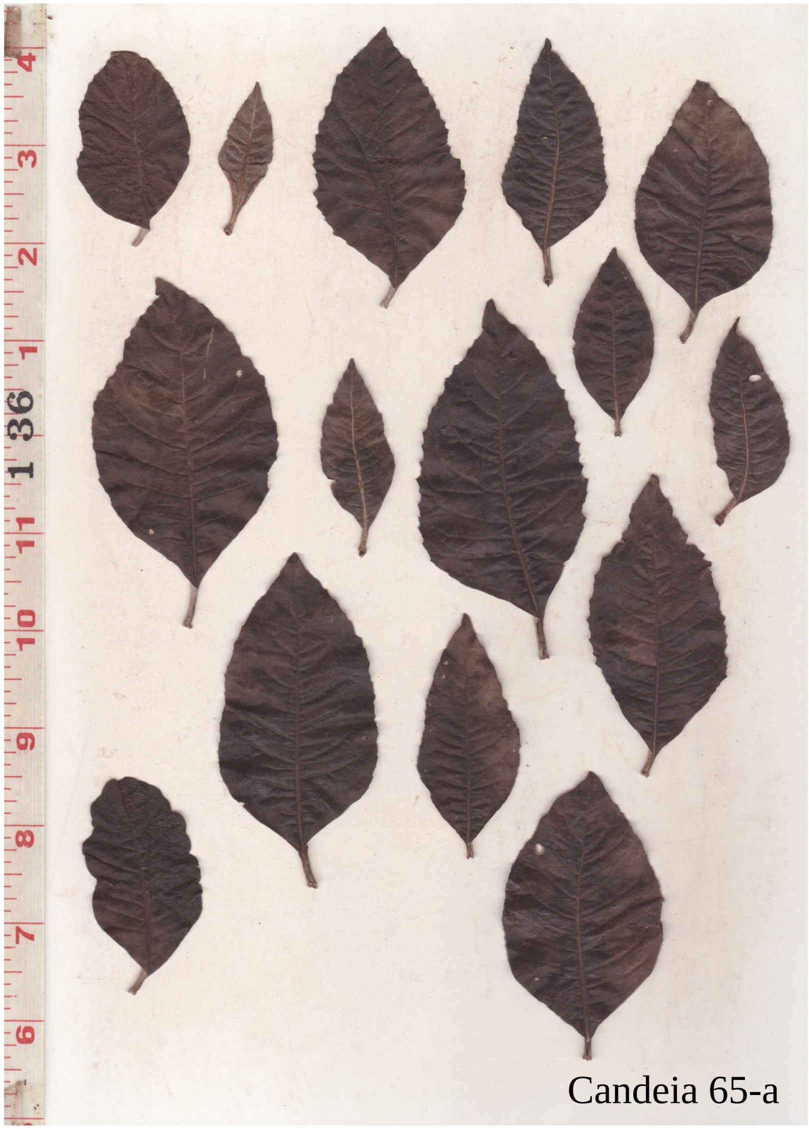

Candeia 65-a

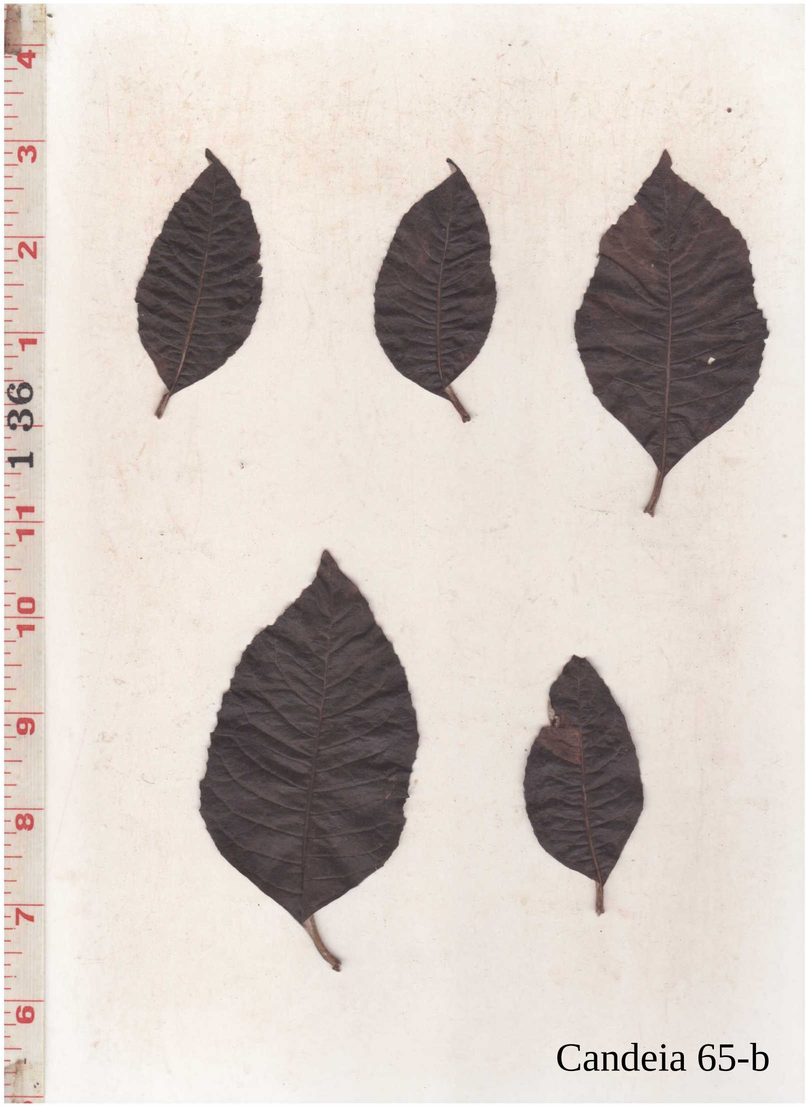

Candeia 65-b

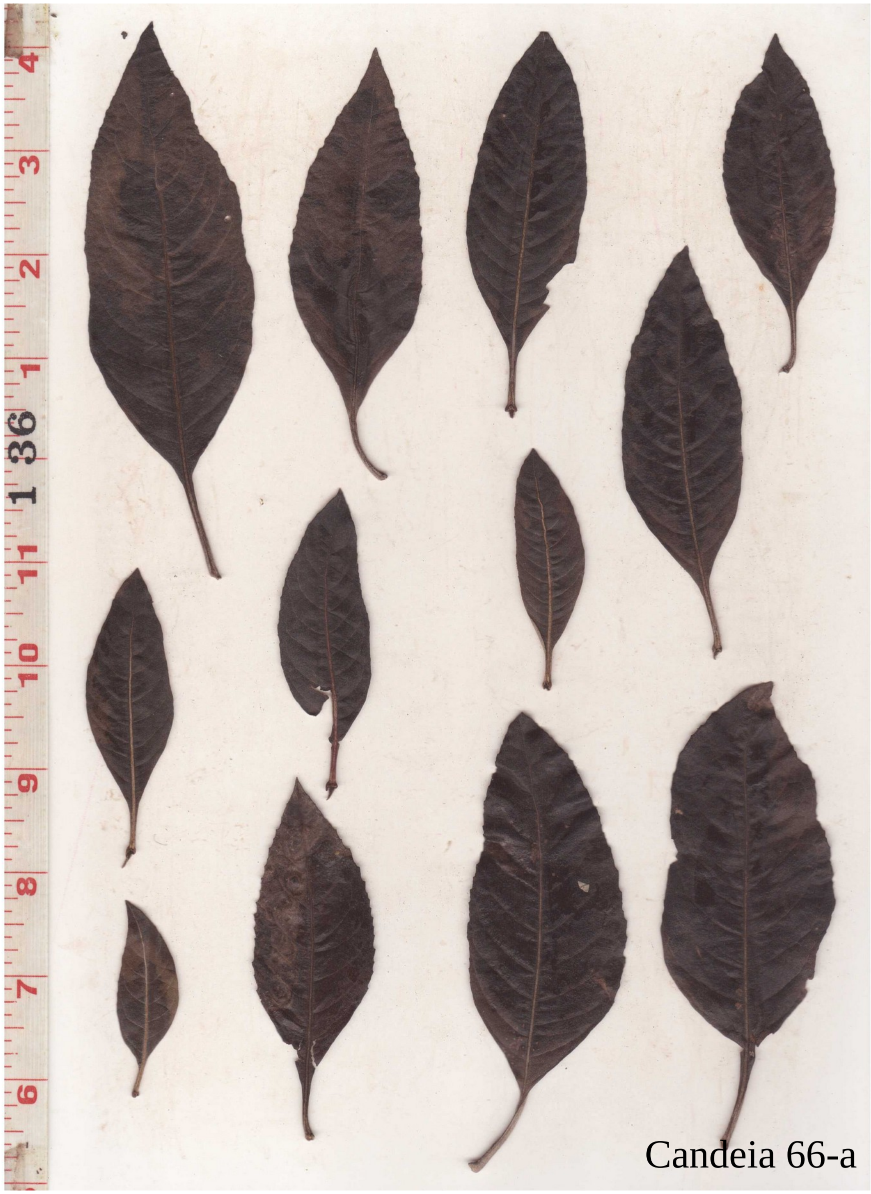

Candeia 66-a

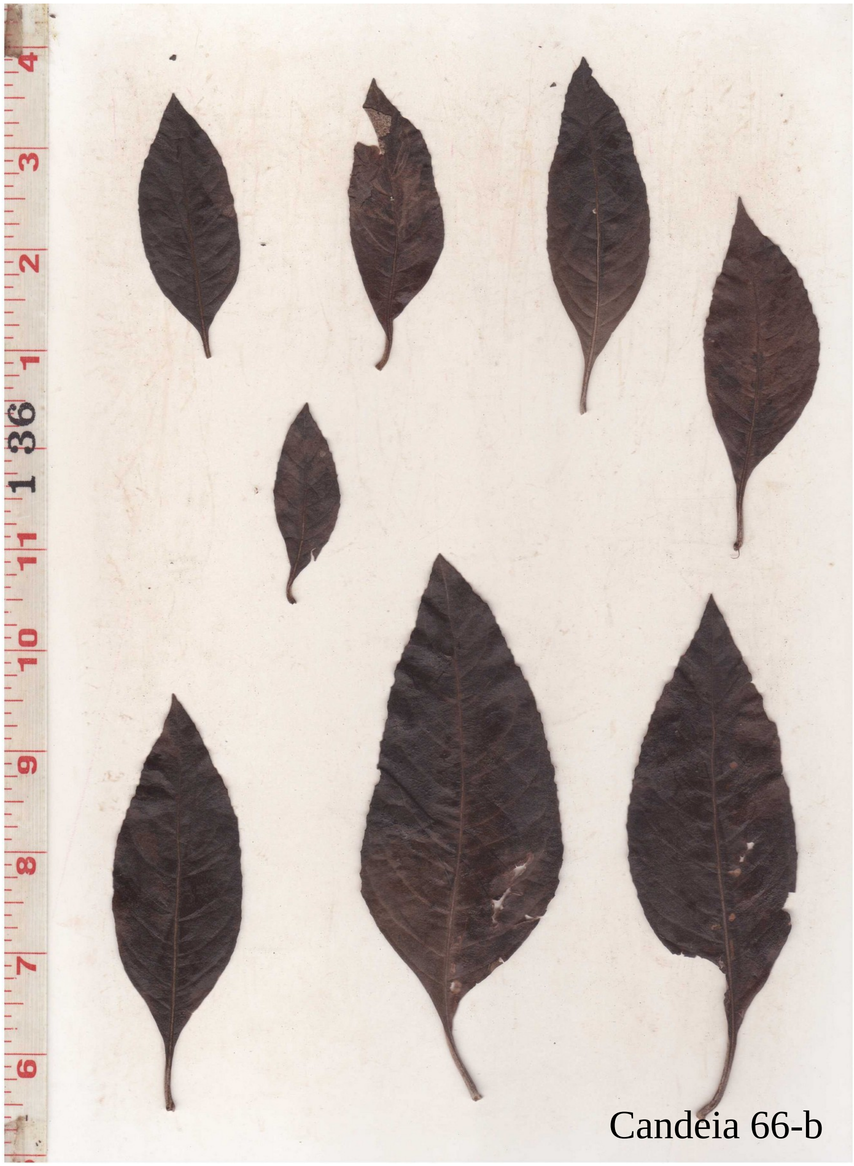

Candeia 66-b

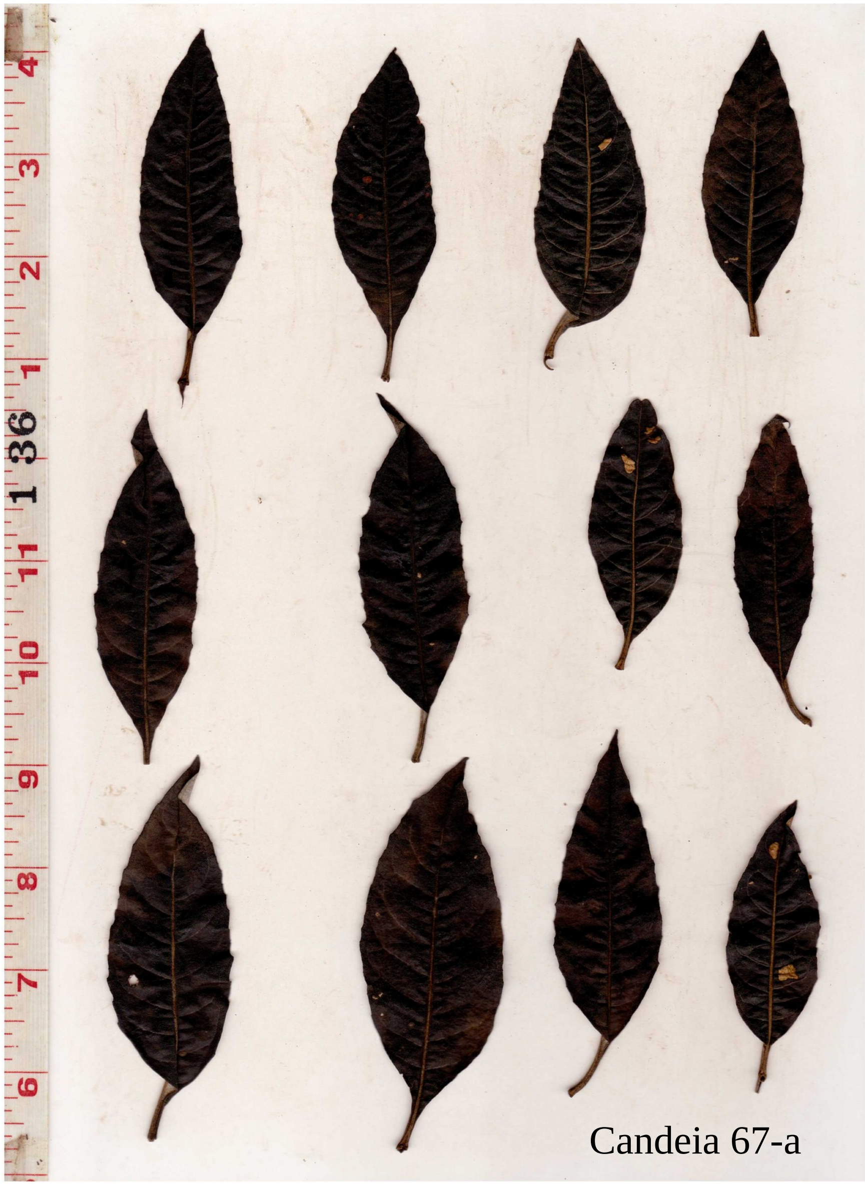

Candeia 67-a

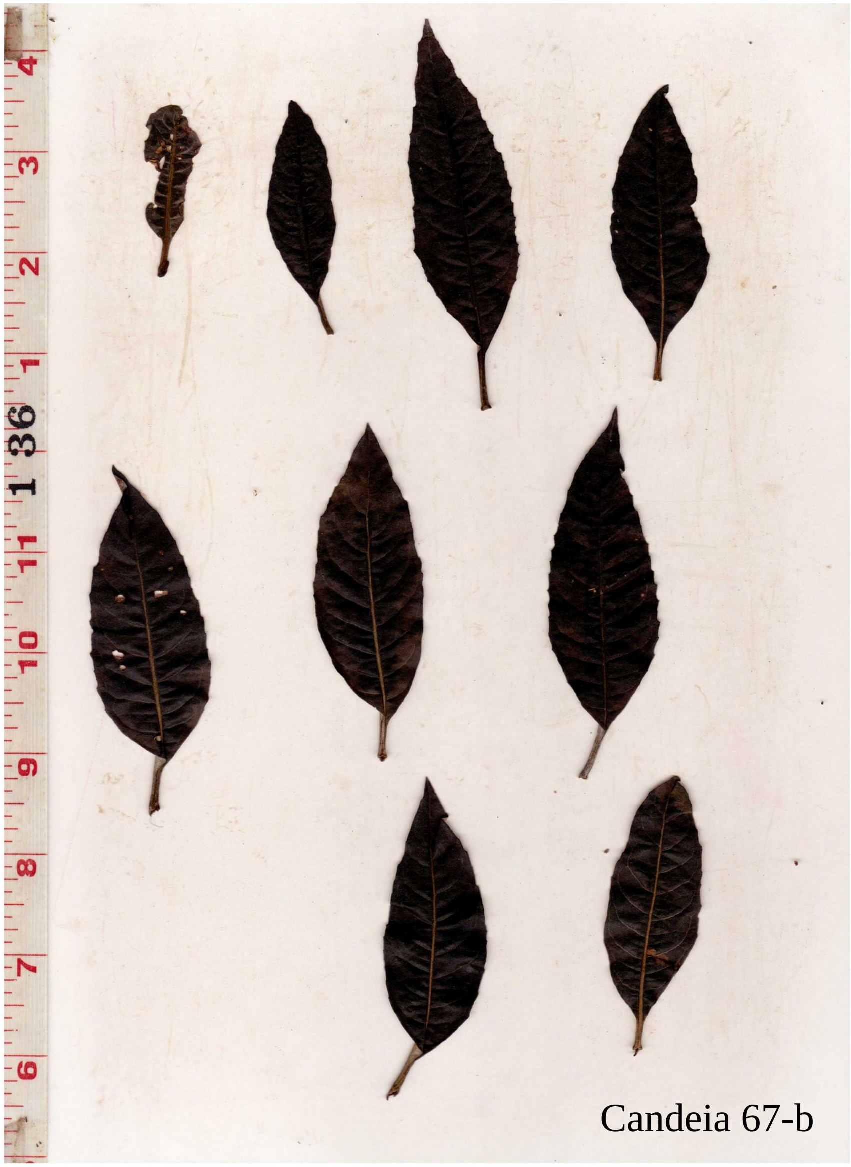

Candeia 67-b

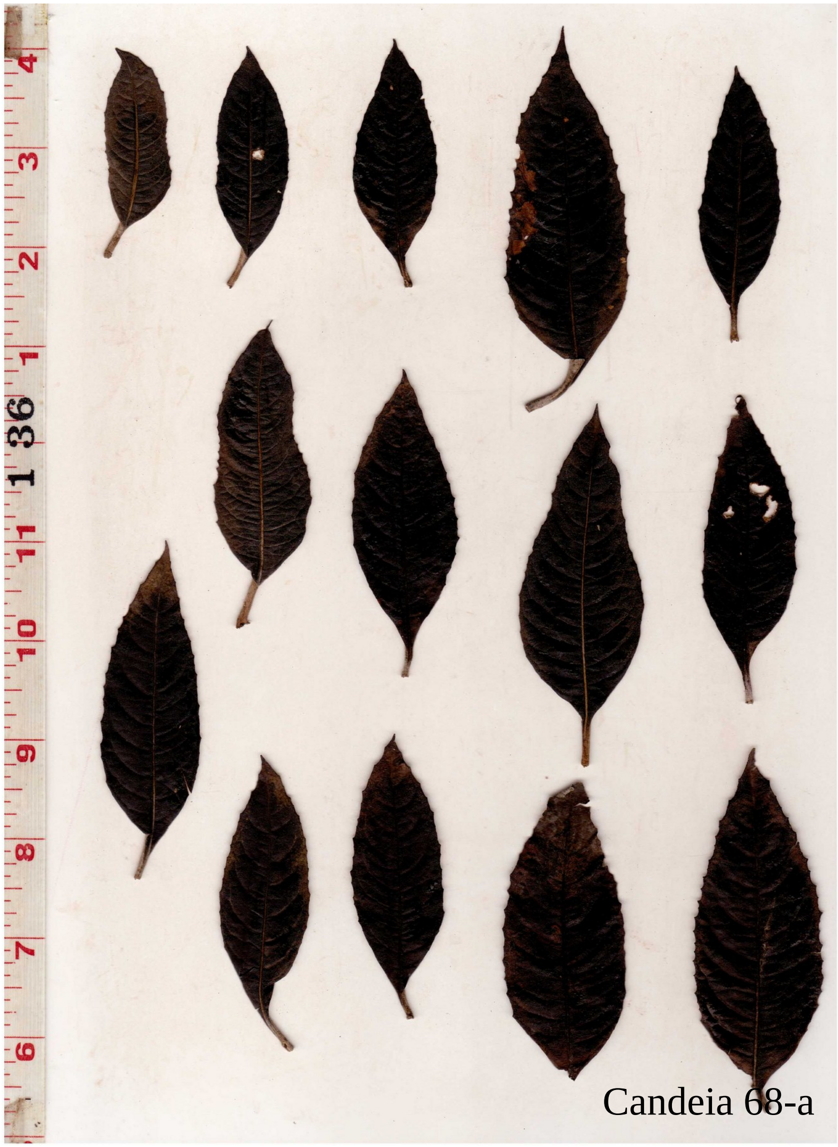

Candeia 68-a

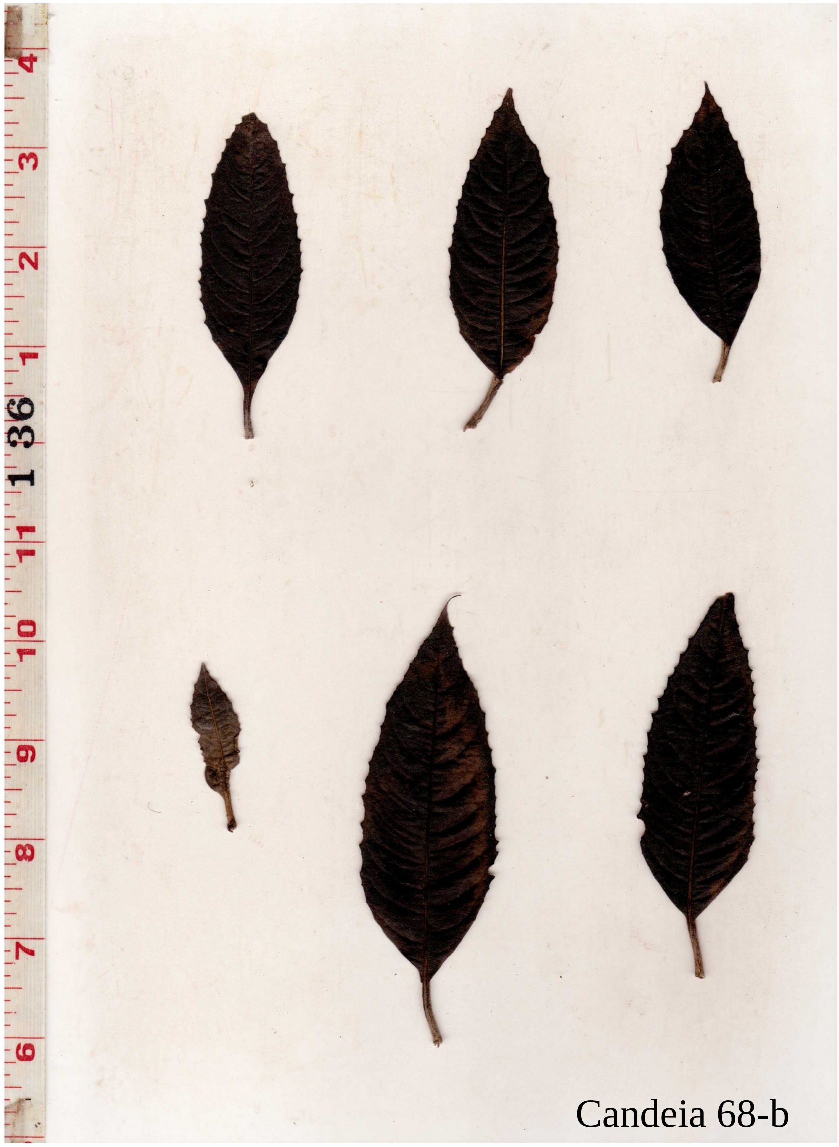

Candeia 68-b
